# Global associations of adolescent health with inclusive and sustainable well-being, 2010–2035

**DOI:** 10.7189/jogh.16.04188

**Published:** 2026-07-03

**Authors:** Tianyu Huang, Yunfei Liu, Hao Cheng, Shan Cai, Jiajia Dang, Jiaxin Li, Ruolan Yang, Juan Yang, Yi Song, Sarah Baird, Susan M Sawyer

**Affiliations:** 1 School of Public Health, Institute of Child and Adolescent Health, Peking University, Beijing, China; 2 School of Science, Minzu University of China, Beijing, China; 3 Xiongan New Area Centre for Public Health Service, Hebei, China; 4 Department of Global Health, Milken Institute School of Public Health, George Washington University, Washington, D.C., USA; 5 Department of Paediatrics, Faculty of Medicine, Dentistry and Health Sciences, The University of Melbourne, Parkville, Australia; 6 Murdoch Children’s Research Institute, Parkville, Victoria, Australia; 7 Centre for Adolescent Health, Royal Children’s Hospital, Parkville, Australia

## Abstract

**Background:**

Exploring how multidimensional social factors are linked with adolescent health could inform policies to achieve equitable and sustainable well-being. We aimed to examine the associations between adolescent health and three dimensions of social development globally.

**Methods:**

We conducted a global ecological analysis across 193 countries using data from the Global Burden of Disease 2021. We analysed age-standardised DALY rates (ASDR) for adolescents aged 10–24 years (2010–2035) in relation to well-being (human development index (HDI)), inclusion (gender inequality index (GII)), and sustainability (adjusted net savings (ANS)). We performed the main analyses using data from 2010 to 2021. We generated forecasts to 2035, presented as scenario analyses, using autoregressive integrated moving average models, and log-linear regressions were used to estimate associations and temporal trends.

**Results:**

Global adolescent ASDR declined from 14 280.1 (95% uncertainty interval (UI) = 14 264.3, 14 296.0) in 2010 to 13 260.5 (95% UI = 13 245.2, 13 275.8) in 2021, and is forecasted to be 11 187.7 (95% UI = 11 173.8, 11 201.6) per 100 000 by 2035. HDI showed attenuating negative associations with all-cause ASDR (rate ratio (RR) = 0.79; 95% confidence interval (CI) = 0.74, 0.84 in 2010 *vs.* RR = 0.84; 95% CI = 0.79, 0.89 in 2021), with further attenuation in the scenario analysis to 2035 (RR = 0.92; 95% CI = 0.88, 0.96). GII demonstrated strengthening positive associations (RR = 1.09; 95% CI = 1.02, 1.15 in 2010 *vs.* RR = 1.11; 95% CI = 1.06, 1.17 in 2021), with further strengthening in the scenario analysis (RR = 1.15; 95% CI = 1.11, 1.20). ANS showed no significant associations in 2010 or 2021, but showed emerging associations with the adolescent health burden of non-communicable diseases (NCDs) in NCD-predominant countries.

**Conclusions:**

While gains in well-being were linked to reduced adolescent health burden, this relationship appeared to attenuate in our main analysis between 2010 and 2021 and the scenario analysis for 2035. Inclusion shows increasingly strong associations with the adolescent health burden across diseases, and sustainability is emerging in developed regions. Findings indicate the benefits of equity-focused, intersectoral policies that promote inclusive development and intergenerational justice.

Adolescents remain vulnerable in a world of rapid globalisation and digital transitions. The second Lancet Commission on adolescent health and well-being indicates that by 2030, at least 50% of the world’s adolescents will live in multi-burden countries where they face multiple, overlapping health burdens, with adolescents in all countries increasingly affected by emerging challenges such as overweight and obesity, mental disorders, and suicide [1]. Despite the significance of this critical developmental stage, adolescents are underrepresented in most national strategies, which threatens the achievement of many Sustainable Development Goals (SDGs) [2–8].

Adolescent health is profoundly influenced by social factors at the personal, family, community, and national levels, among which structural determinants (*e.g.* social systems and the wider development landscape) are considered to have the most significant and far-reaching effects [9,10]. The biological and socialisation processes that underpin healthy adolescent development are powerfully shaped by external environments. Supportive societies empower and protect adolescents, while adverse contexts and inequality impose substantial constraints on adolescents’ opportunities and potential [2,9,11–13]. Notwithstanding studies examining the social determinants of adolescent health [11,14], critical gaps persist. Most studies focus on individual-level determinants; few have explored how country-level development across multiple dimensions affects adolescent health, particularly beyond economic development. Moreover, while existing research highlights improvements in some areas like HIV, a limited intersectoral perspective and lack of global data coverage have resulted in widespread neglect of newer health concerns, such as violence and mental health [15].

A recent review in Lancet Planetary Health proposed a multidimensional framework on well-being, inclusion, and sustainability to better capture the relationships between structural social determinants and health, emphasising long-term and intergenerational equity [16]. This approach is especially relevant for adolescents, whose health today drives future societal health and well-being [17]. Using this multidimensional framework of well-being (human development index (HDI)), inclusion (gender inequality index (GII)), and sustainability (adjusted net savings (ANS)), we quantified the associations of these three dimensions with the adolescent health burden across 193 countries in 2010, 2021, and with projections to 2035. We analysed all-cause disability-adjusted life years (DALYs) from the Global Burden of Disease (GBD) 2021 database (including DALY estimates from 2010 to 2021 and forecasts in 2035), with development indicators sourced from the United Nations Development Programme (UNDP) (HDI and GII) and the World Bank (ANS) [18,19]. Specifically, we aimed to: determine the global, regional and national trends of disease burden among adolescents aged 10–24 years [20], determine the relative contributions of HDI, GII, and ANS to the adolescent disease burden, and examine temporal trends in these associations across the period of global social transformations that is marked by interconnected economic and cultural change, rapid technological advancement, and epidemiological transitions. We aimed to identify which dimensions are becoming increasingly critical for adolescent health policymaking, ultimately promoting inclusive and sustainable well-being for the next generation. We followed the GRABDROP guidelines (Table S1 of the **Online Supplementary Document**) [21].

## METHODS

### Overview

We applied a global ecological design. We used GBD 2021 data for past estimates from 1990 to 2021 and GBD forecasting data for 2035. We accessed the HDI, GII, and ANS values from 1990 to 2021 from online databases, and projected the 2035 values using the autoregressive integrated moving average (ARIMA) model. We focused on 193 countries and territories based on data availability and quality (Table S2 in the **Online Supplementary Document**). We performed descriptive and log-linear regression analyses to examine the associations between each dimension and adolescent DALYs between 2010 and 2021, and further conducted scenario analyses through 2035.

### DALYs

The DALY is a composite metric of years of life lost and years lived with disability, enabling a comprehensive assessment of fatal and non-fatal health loss across diseases and capturing broader impacts on multimorbidity and functional health status [22]. We obtained annual DALYs from 2010 to 2021 per 100 000 population were obtained from the GBD 2021 Results Tool (Institute for Health Metrics and Evaluation, University of Washington, Seattle, Washington, USA) at the country level, while we obtained the predicted DALYs in 2035 from GBD foresight. GBD 2021 produced the estimates of DALYs for 371 diseases and injuries by sex, age, and year in 204 countries and territories [23]. The details of GBD 2021 have been published elsewhere [23]. In brief, the 371 diseases and injuries were organised within a four-level cause hierarchy, with level one comprising four broad aggregate categories (communicable, maternal, neonatal, and nutritional diseases (CMNNs); non-communicable diseases (NCDs); injuries; and other COVID-19 pandemic-related outcomes). These level-1 categories are further classified into 22 level-2 categories. Given data availability and consistency, we included only the first three level-1 categories (CMNNs, NCDs, and injuries). We used meta-regression-Bayesian, regularised, trimmed, and Disease Modelling Meta-Regression, version 2.1 (Institute for Health Metrics and Evaluation, University of Washington, Seattle, Washington, USA), as well as Cause of Death Ensemble model (Institute for Health Metrics and Evaluation, University of Washington, Seattle, Washington, USA) to estimate the DALYs by location, age, sex, year, and cause between 1990 and 2021. We generated the 2035 estimates were generated using an ARIMA model with decaying drift and an exogenous variable, the socioeconomic development index (SDI) [24].

We considered age-standardised all-cause and cause-specific (level 2) DALY rates (per 100 000 population) among those aged 10–24 years in both sexes as the primary outcome. To minimise the influence of differences in age structure across countries and to improve cross-country comparability of adolescent health burden, we age-standardised all-cause and level-two-cause-specific DALY rates in 2010, 2021, and 2035 for both sexes and all locations to generate the age-standardised DALY rates (ASDRs) using the world population structure 2021 as reported by GBD [25].

### Well-being, inclusion, and sustainability

The HDI, representing well-being, is a commonly used measure of population development created by the UNDP based on three key dimensions – a long and healthy life, knowledge, and decent living, which were calculated using life expectancy at birth, mean years and expected years of schooling, and gross domestic product (GDP) *per capita*, respectively [18]. The GII, representing inclusion, is a well-acknowledged indicator reflecting population-level gender inequities also created by the United Nations (UN), which includes three important dimensions – reproductive health (represented by maternal mortality and adolescent birth rate), empowerment (represented by the proportion of men and women with at least some secondary education and the proportion of men and women occupying parliamentary seats), and economic status (labour market participation among men and women) [19]. The detailed calculation processes for the HDI and GII are presented in the Human Development Report 2023/2024 Technical Notes published by UNDP. The data centre of the UNDP Human Development Reports provides the HDI and GII, along with their components, for 195 countries from 1990 to 2022. The ANS, representing sustainability, is a widely used measure of national economic sustainability developed by the World Bank, defined as net investment in produced capital plus public education expenditure minus natural resource depletion and damage from carbon dioxide and particulate emissions [26]. The World Bank’s database provides the ANS and its components, measured by annual percentage growth or in constant 2015 USD. We used the former measurement in this study.

To generate the forecast estimates of the HDI, GII, and ANS in 2035, we applied an ARIMA model for every component of these indicators to gain their future estimates, which were then calculated according to the technical directions from the UNDP and World Bank to produce the forecast HDI, GII, and ANS in 2035.

### Country groups

We divided countries into three groups based on the excess adolescent disease burden representing different stages of the epidemiological transition, according to the definitions from the 2016 and 2025 Lancet Commissions on Adolescent Health and Well-being [1,17]. The groupings in this study are based on the DALY rate for adolescents aged 10–24 years in 2021 and are applied consistently across analyses. We defined countries as multi-burden if adolescents aged 10–24 years had a burden of CMNN diseases ≥2500 DALYs per 100 000, injury excess if adolescents were estimated to have ≥2500 DALYs per 100 000 due to injury and <2500 DALYs due to CMNN, and NCD predominant if both injuries and CMNN each contributed <2500 DALYs per 100 000 to the disease burden (Table S2 of the **Online Supplementary Document**). To delve further into the association between the adolescent health burden and its different dimensions, we also divided countries into Global North and Global South groups based on the list provided by the UN Finance Centre for South-South Cooperation.

### Statistical analysis

We estimated annual percentage change (APC) by fitting a log-linear regression model to the ASDRs or their male-to-female ratios over the period 2010–2021. We employed log-linear regression models to quantify cross-sectional associations between sustainable development dimensions (HDI, GII, and ANS) and ASDRs (all-cause and level-2 cause-specific) at two time points (2010 and 2021), and further conducted scenario analyses to 2035. Before analysis, we standardised all predictors (Z-scores). The model was specified as ln (ASDR) = *β*_0_ + *β*_1_HDI + *β*_2_GII + *β*_3_ANS + ϵ.

We exponentiated the coefficients to obtain rate ratios (RRs) representing the multiplicative change in ASDR per one-standard-deviation increase in each predictor. Robust standard errors accounted for heteroskedasticity. We calculated variance inflation factors (VIFs) to assess multicollinearity among indicators. Further, we conducted subgroup analysis in the three country groups and the Global North/South groups. To capture the evolving associations of the three dimensions with adolescent health burdens, temporal trends in these associations were assessed by fitting log-linear regression models of RRs on calendar year (2010–2021). We derived the APC, and the corresponding *P*-values from these models to evaluate the statistical significance of trends over time.

We conducted sensitivity analyses to assess the robustness of the findings. First, we performed age-stratified analyses (10–14, 15–19, and 20–24 years) to examine consistency across age groups. Second, we re-estimated models with regional fixed effects to account for unobserved spatial heterogeneity. Third, we conducted lagged analyses (one to three years) and restricted cubic spline analysis to evaluate potential delayed and nonlinear associations between HDI, GII, ANS, and ASDR. Finally, we performed sex-stratified analyses and tested interaction terms between sex and each indicator to assess potential effect modification. We conducted all analyses in *R*, version 4.4.2 (R Core Team, Vienna, Austria), and *P* < 0.05 was considered statistically significant.

## RESULTS

### Global trends in all-cause DALYs from 2010 to 2035

**Figure 1:**
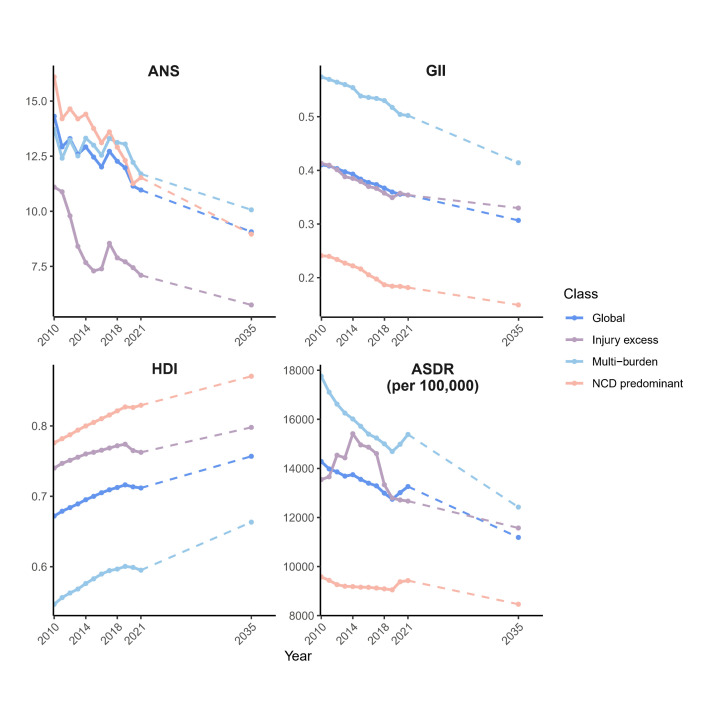
Trends in all-cause age-standardised DALY rate, HDI, GII, and ANS in 193 countries, 2010–2035.

The global all-cause ASDR in adolescents aged 10–24 years exhibited a downward trend from 2010 (ASDR = 14 280.1; 95% uncertainty interval (UI) = 14 264.3, 14 296.0 per 100 000) to 2021 (ASDR = 13 260.5; 95% UI = 13 245.2, 13 275.8 per 100 000), with an APC of –0.85% (95% confidence interval (CI) = –1.12, –0.58), and is forecast to decline (ASDR = 11 187.7; 95% UI = 11 173.8, 11 201.6 per 100 000) in 2035. Across the three country groups, the all-cause ASDR corresponded with global trends. Multi-burden countries continued to have the highest adolescent health burden of the three groups. The ASDR decreased from 17 755.7 (95% UI = 17 739.6, 17 771.8) per 100 000 population in 2010 to 15 384.3 (95% UI = 15 370.0, 15 398.6) per 100 000 population in 2021 (APC = –1.47; 95% CI = –1.93, –1.01), and is forecast to decline to 12 423.2 (95% CI = 12 410.5, 12 435.84) per 100 000 in 2035. The ASDR in NCD predominant countries remained relatively stable as the lowest among the three groups, with a slight decrease from 2010 (ASDR = 9575.5; 95% UI = 9561.6, 9589.4 per 100 000) to 2021 (ASDR = 9427.8; 95% UI = 9412.7, 9442.9 per 100 000), indicating a non-significant change (APC = –0.16; 95% CI = –0.49, 0.16), that is forecast to reach 8464.2 (95% UI = 8450.0, 8478.4) per 100 000 in 2035. (**Figure 1**; Table S2 in the **Online Supplementary Document**)

### Global trends in well-being, inclusion, and sustainability from 2010 to 2035

The HDI progressed globally from 2010 (HDI = 0.67) to 2021 (HDI = 0.71), and is forecast to continue increasing to 2035 (HDI = 0.76). Similar trends were found in multi-burden (2010 HDI = 0.55, 2021 HDI = 0.59, and 2035 HDI = 0.66), injury excess (2010 HDI = 0.74, 2021 HDI = 0.76, and 2035 HDI = 0.80), and NCD predominant countries (2010 HDI = 0.78, 2021 HDI = 0.83, and 2035 HDI = 0.87). The GII declined globally from 2010 (GII = 0.41) to 2021 (GII = 0.35) and is forecast to continue declining through 2035 (GII = 0.31), reflecting an improvement in the inclusion dimension. Similar trends were found in multi-burden countries (2010 GII = 0.57, 2021 GII = 0.50, and 2035 GII = 0.41, the highest group) and NCD-predominant countries (2010 GII = 0.24, 2021 GII = 0.18, and 2035 GII = 0.15, the lowest group). In contrast to these two indicators, the ANS exhibited greater volatility, with ANS in injury-excess countries significantly lower than in the other two country groups. Globally, the ANS showed a downward trend from 2010 (ANS = 14.31) to 2021 (ANS = 10.96) and is forecast to continue declining through 2035 (ANS = 9.07). The decline was most pronounced in NCD predominant countries (2010 ANS = 16.09, 2021 ANS = 11.53, and 2035 ANS = 8.96), while the decrease in multi-burden countries was relatively modest (2010 ANS = 13.70, 2021 ANS = 11.70, and 2035 ANS = 10.07). (**Figure 1**; Table S3 in the **Online Supplementary Document**)

### Regional and national patterns of DALYs and average change from 2010 to 2035

**Figure 2:**
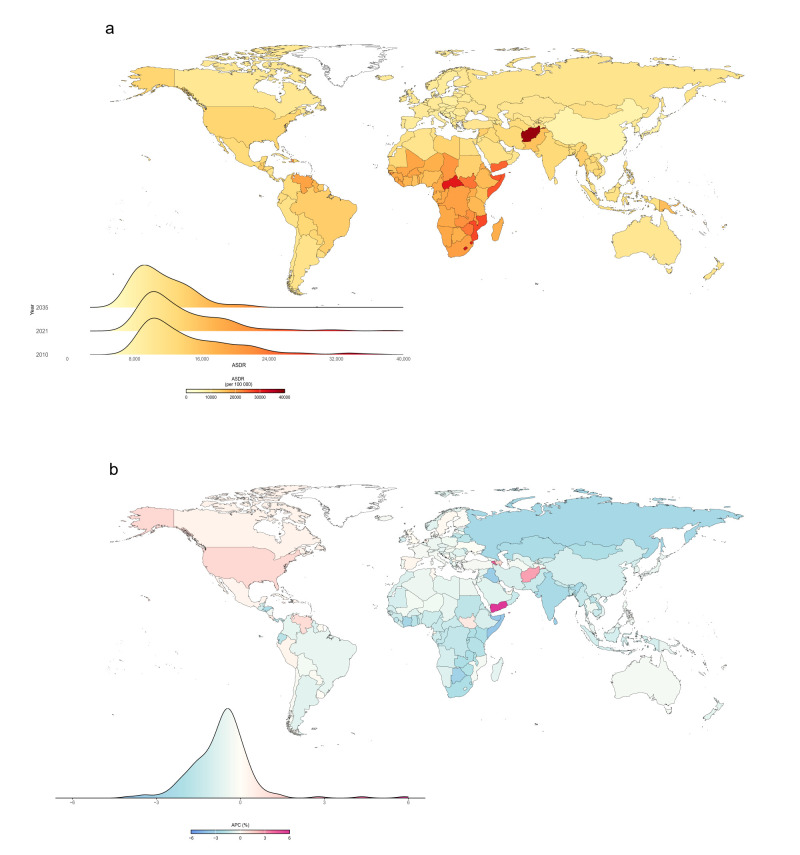
Regional patterns of all-cause ASDR in 2021 (**Panel A**) and APC in 2010–2021 (**Panel B**) for 193 countries.

In 2021, the highest all-cause DALY burden among adolescents was concentrated in Sub-Saharan Africa and certain low-income regions; 14 countries (7% and all from the multi-burden group) exhibited ASDRs exceeding 20 000 per 100 000. The highest ASDRs were documented in Afghanistan (ASDR = 38 224.7; 95% UI = 38 187.0, 38 262.3), Lesotho (ASDR = 32 679.9; 95% UI = 32 536.8, 32 823.6), and the Central African Republic (ASDR = 31 303.6; 95% UI = 31 221.2, 31 386.3). Those countries also experienced substantial gender disparity, with the male-to-female ASDR ratio of 1.87 in Afghanistan, 1.13 in Lesotho, and 1.40 in the Central African Republic. In contrast, the lowest burden was observed in East Asia and Europe, with 51 countries (26% and 49 NCD-predominant countries and two injury-excess countries) reporting ASDRs below 10 000 per 100 000. Three NCD predominant countries, namely China (ASDR = 7515.8; 95% UI = 7512.3, 7519.4), Singapore (ASDR = 7716.8; 95% UI = 7653.2, 7780.7), and the Republic of Korea (ASDR = 7778.6; 95% UI = 7759.0, 7798.2) demonstrated the most favourable outcomes. (**Figure 2**; Figure S1 in the **Online Supplementary Document**)

From 2010 to 2021, 109 of 193 (56%) countries exhibited a significant decline in the ASDR, mainly among multi-burden countries, with the sharpest drops observed in Somalia (APC = –4.0; 95% CI = –7.5, –0.5) %), Sri Lanka (APC = –3.4; 95% CI = –6.0, –0.9), and Botswana (APC = –3.0; 95% CI = –3.8, –2.3). In contrast, only eight of 193 (4%) countries experienced significant increases, half of which were from the NCD predominant group, namely the USA (APC = 1.3; 95% CI = 0.7, 1.8), Mauritius (APC = 0.9; 95% CI = 0.8, 1.0), Jamaica (APC = 0.7; 95% CI = 0.0, 1.5), and Canada (APC = 0.3; 95% CI = 0.1, 0.6). (**Figure 2**; Figure S1 in the **Online Supplementary Document**)

### Association between all-cause DALYs and HDI, GII, and ANS from 2010 to 2035

**Figure 3:**
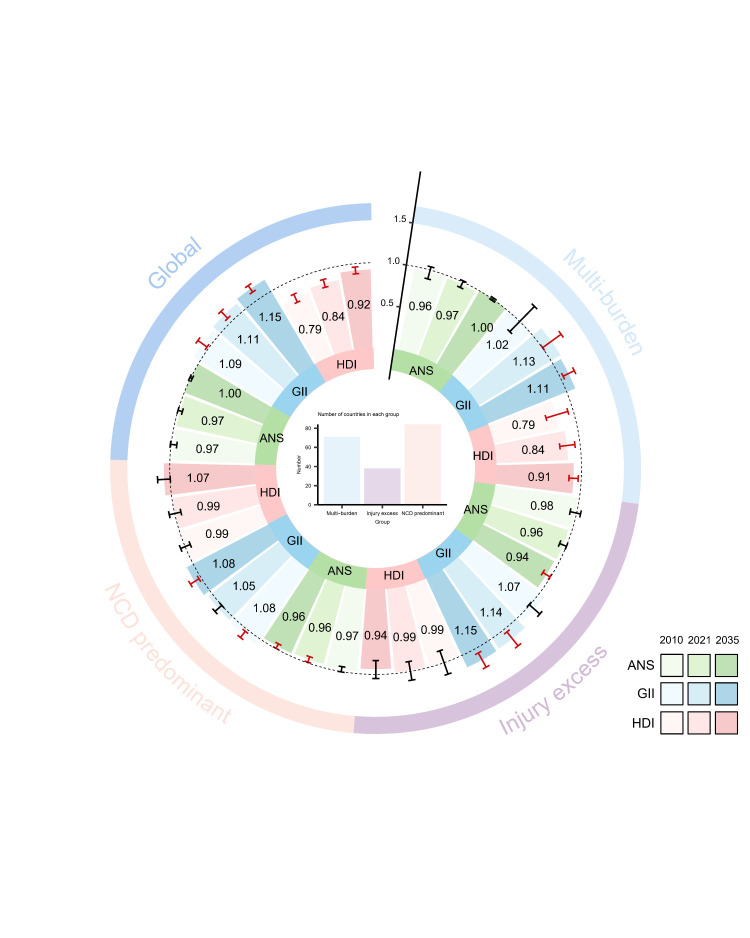
Association of all-cause ASDR with HDI, GII, and ANS in different country groups in 2010, 2021,2035.

Across all models, VIF values for each indicator were <5, indicating no significant multicollinearity among the HDI, GII, and ANS. Overall, the HDI exhibited significant, weakening negative associations, whereas the GII showed significant, strengthening positive associations with all-cause ASDR among adolescents globally. From 2010 to 2021, the association between HDI and all-cause ASDR appeared to attenuate (RR = 0.79; 95% CI = 0.74, 0.84 in 2010 *vs.* RR = 0.84; 95% CI = 0.79, 0.89 in 2021), and the projected estimate for 2035 (RR = 0.92; 95% CI = 0.88, 0.96) suggested a further attenuation in the scenario analysis. The association between GII and all-cause ASDR was slightly higher in 2021 (RR = 1.11; 95% CI = 1.06, 1.17) than in 2010 (RR = 1.09; 1.02, 1.15), and the projected estimate for 2035 (RR = 1.15; 95% CI = 1.11, 1.20) suggested strengthening in scenario analysis. No significant association was observed between ANS and ASDR in 2010 or 2021, and the projected estimate for 2035 likewise suggested no clear association in scenario analysis. (**Figure 3**; Table S4 in the **Online Supplementary Document**)

In both multi-burden and injury excess countries, the association between GII and ASDR appeared to strengthen between 2010 (RR = 1.02; 95% CI = 0.83, 1.25 and RR = 1.07; 95% CI = 0.99, 1.16) and 2021 (RR = 1.13; 95% CI = 1.00, 1.27 and RR = 1.14; 95% CI = 1.04, 1.26), which was also validated in the scenario analysis (RR = 1.11; 95% CI = 1.04, 1.18 and RR = 1.15; 95% CI = 1.06, 1.25) (**Figure 3**; Table S4 and S9 in the **Online Supplementary Document**). Global North and Global South countries exhibited distinct patterns. In Global North countries, the HDI was not significantly associated with all-cause ASDR in most periods, whereas GII exhibited a progressively stronger positive association from 2010 (RR = 1.15; 95% CI = 1.07, 1.22) to 2021 (RR = 1.19; 95% CI = 1.10, 1.28). In the 2035 scenario analysis, the association between GII and ASDR remained significant in Global North countries (RR = 1.16; 95% CI = 1.08, 1.24). The ANS was not significantly associated with ASDR in Global North countries in either 2010 or 2021, nor in the 2035 scenario analysis. In contrast, Global South countries showed a consistent negative association between HDI and all-cause ASDR across all years, with the magnitude remaining relatively stable over time (RR = 0.78; 95% CI = 0.72, 0.85 in 2010 and RR = 0.82; 95% CI = 0.76, 0.87 in 2021). Meanwhile, the association between GII and all-cause ASDR remained relatively stable in Global South countries (RR = 1.10; 95% CI = 1.01, 1.20 in 2010 and RR = 1.12; 95% CI = 1.05, 1.20 in 2021). The ANS showed a significant association with ASDR in 2021 (RR = 0.95; 95% CI = 0.93, 0.99), but no consistent trend in the association was found. In the 2035 scenario analysis, these trends were also validated in Global South countries (for HDI RR = 0.91; 95% CI = 0.87, 0.96, for GII RR = 1.14; 95% CI = 1.09, 1.20, and for ANS RR = 0.99; 95% CI = 0.98, 1.01) (Figure S2 and Table S4 in the **Online Supplementary Document****)**

Sensitivity analyses yielded results consistent with the main findings. Age-stratified analyses and models adjusted for regional fixed effects showed associations similar to those in the primary analyses (Tables S6 and S7 in the **Online Supplementary Document**). The lagged analyses showed that ANS was negatively associated with the adolescent health burden in 2010 and 2021, with a significant association in 2010 at lag of one year (RR = 0.95; 95% CI = 0.91, 0.99) and in 2021 across all lag specifications. In scenario analyses for 2035, significant associations between ANS and ASDR were observed at a lag of three years (RR = 0.96; 95% CI = 0.93, 0.98) (Table S8 in the **Online Supplementary Document**). A significant nonlinear relationship was consistently observed for HDI in 2010, 2021, and 2035 (*P* < 0.01), and for ANS in 2021 (*P* = 0.002), whereas no evidence of nonlinearity was found for GII. In sex-stratified analyses, the associations were comparable between males and females, and no statistically significant interaction effects were observed (Figure S3 and Table S10 in the **Online Supplementary Document**).

### Associations between DALYs caused by different level 2 causes and HDI, GII, and ANS

In analyses of 2010 and 2021, with scenario projections for 2035, the associations of HDI, GII, and ANS with ASDR in 10–24-year-olds showed distinct patterns across different level 1 and level 2 causes. The HDI showed a declining negative association with CMNNs, but a positive association with specific NCDs. The GII showed associations across disease categories and the ANS maintained significant associations with limited level 2 causes. (Figure S4 and Table S5 in the **Online Supplementary Document**)

For CMNNs, the association between HDI and ASDRs attenuated over time, with RRs increasing from 0.40 (95%CI = 0.36, 0.45) in 2010 to 0.47 (95% CI = 0.42, 0.54) in 2021, and further to 0.60 (95% CI = 0.54, 0.66) in 2035 in the scenario analysis. In contrast, the association between GII and ASDRs remained stable between 2010 (RR = 1.17; 95% CI = 1.05, 1.30) and 2021 (RR = 1.19; 95% CI = 1.06, 1.32), but became stronger in 2035 (RR = 1.35; 95% CI = 1.22, 1.48) in the scenario analysis. For NCDs, stable associations between HDI, GII, and ANS and ASDR were observed from 2010 to 2021. For injuries, a stable association between GII and ASDR was found between 2010 (RR = 1.27; 95% CI = 1.12, 1.44) and 2021 (RR = 1.36; 95% CI = 1.21, 1.53). (Figure S4 and Table S5 in the **Online Supplementary Document**).

## DISCUSSION

The future lives of adolescents are closely and deeply linked to inclusive and sustainable well-being. To our knowledge, this ecological study is the first to integrate data from the GBD database, the UNDP, and the World Bank to reveal the evolving associations of health burdens in 10–24-year-olds with three indicators representing well-being (HDI), inclusion (GII), and sustainability (ANS) from 2010 to 2021, with forecasts to 2035 in the scenario analysis. We found divergent trends in adolescent health across different aspects of national development. The most substantial reductions in disease burden were observed in multi-burden countries, whereas several high-income regions experienced stagnating or even increasing burdens over time. Interestingly, countries with higher adolescent health burdens also tended to exhibit greater male-to-female disparities in ASDR. The association between the HDI and health burden was attenuated with national development, whereas the GII demonstrated progressively stronger associations over time and with the spectrum of national development. Cause-specific analyses showed that the HDI was primarily associated with CMNNs, the GII developed broadening associations with CMNNs, NCDs, and injuries, while the ANS showed emerging associations with ASDR in injury-excess countries and NCD-predominant countries. These findings indicate that, as the epidemiological transition of adolescent health burden occurs with socioeconomic development around the globe, greater attention should be directed toward fostering inclusion to ensure that the health needs of all adolescents are recognised and addressed in research and policy. Strengthening inclusion may help reduce inequities and support more equitable and sustainable health strategies in the years ahead.

### Changes in adolescent DALYs across different regions

Across the spectrum of national development, adolescent health burdens follow divergent trends. While some countries have seen gains alongside socioeconomic progress, others, particularly conflict-affected regions, face persistently high burdens. Emerging NCD threats and the impact of COVID-19 further compound these challenges, underscoring the need to examine disparities, including those linked to gender inequalities [1]. Our sex-disaggregated analysis echoed this concern, revealing elevated male-to-female ASDR ratios in high-GII countries across the spectrum of national development, from multi-burden countries like Yemen to high-HDI countries like Brazil. These findings highlight the potential value of multidimensional frameworks, including gender equity and broader determinants than economic indicators, when addressing the adolescent health burden. Indeed, recent research on women’s health and well-being has explicitly articulated similar concerns, proposing a life-course, multidimensional metric framework that not only evaluates progress and challenges in women’s welfare but also helps identify exemplar countries whose trajectories can inform policy elsewhere [27].

### Associations between DALYs and beyond GDP indicators

The inclusion dimension, as represented by the GII, showed a strong relationship with the adolescent disease burden, highlighting an important yet underdeveloped frontier in adolescent health research. From the life course perspective, health differences between men and women begin in adolescence, when pubertal changes and gender socialisation gain prominence and largely coincide with the development of gender gaps [28–30]. Our findings additionally highlight that gender inequality has had an increasingly pronounced association with adolescent health over time, particularly in multi-burden and injury-excess countries. The associations between inclusion and the adolescent health burden were stable for NCDs and injuries during 2010 and 2021. These associations are not yet sufficiently recognised within discussions of gender and health, but they are increasingly relevant in low-, middle-, and high-income countries [29]. Emerging evidence has linked gender norms with the potential risk of mental illness, chronic disease, and violence, reinforcing the need to address gender inequality within diverse health domains [28,31–34]. Inclusive policies that affirm gender diversity and promote safe, equitable environments have been linked to reduced psychological distress among adolescents [35,36]. This suggests that addressing gender inequalities during adolescence is not only an ethical imperative but also a strategic investment in long-term health [37]. Despite this growing recognition, conceptual clarity, definitions, and measurement tools for inclusion remain underdeveloped and poorly standardised [38]. The lack of robust, comparable indicators for equity and inclusion hampers the ability to assess progress and design targeted interventions. Establishing stronger measures of equity and inclusion, including those most relevant for adolescents, could help resolve definitional ambiguities, enable systematic evaluation, and guide policy actions to create safer, more equitable environments for healthier, more empowered generations.

The well-being dimension, represented by the HDI, showed significant negative associations with all-cause DALYs among adolescents globally, with the association most pronounced in multi-burden and Global South countries. This pattern is consistent with the observed nonlinear relationship, in which the inverse association between HDI and ASDR was stronger at lower HDI levels and tended to plateau as HDI increased. This trend also corresponded with the cause-specific analysis, which indicated that the HDI was primarily associated with CMNN diseases – a major contributor to the adolescent disease burden in multi-burden countries. High-HDI countries tend to ensure comprehensive coverage of essential health services, sanitation, food security, and human resources for health, conditions associated with lower burdens of CMNN diseases [10,39,40]. The most typical example is observed in neglected tropical diseases and malaria, which exhibited the strongest correlation with the HDI. These diseases predominantly cluster in regions characterised by extreme poverty and low socioeconomic status, where health care and access to clean water remain severely deficient. This disease burden further exacerbates poverty through lost productivity and high medical costs [41,42]. However, for some diseases, such as mental disorders and substance use disorders, higher HDI is correlated with a higher disease burden, which warrants further attention [43–45]. In the main analyses for 2010 and 2021, the association between HDI and all-cause ASDR appeared to attenuate as the epidemiological transition progressed, a pattern also observed in the scenario analyses for 2035. Although these scenario analyses may be affected by potential circularity within the GBD forecasting framework due to the overlap between HDI and SDI, this pattern nevertheless highlights the need to expand existing frameworks to incorporate a broader range of indicators that influence adolescent health.

Our findings revealed only limited associations between the ANS and adolescent DALYs in the earlier years, with significant correlations emerging in more recent years, especially in injury-excess and NCD-predominant countries. In addition, the nonlinear analysis suggested a significant nonlinear association between ANS and ASDR in 2021, indicating that this relationship may vary across different levels of ANS. This pattern suggests that while the ANS captures long-term investments in economic and environmental capital, its association with adolescent health may be more crucial in the future, particularly in contexts where immediate determinants such as health care access, education quality, and social protection play a more dominant role than in the past. Moreover, a growing body of evidence has underscored the profound health risks posed by unsustainable environmental practices such as fossil fuel dependency and the intensification of climate change [46,47]. These factors include increased exposure to extreme weather events, air pollution, food insecurity, and forced displacement, all of which pose disproportionate risks to vulnerable populations, including adolescents [32,47–49]. Without integrated policies that align sustainability with health equity and adolescent-centred investment, the long-term well-being of future generations remains precarious.

Much adolescent health research and related policies have focused on single indicators and have overlooked broader determinants critical to healthy adolescent development. Adolescents grow up within complex social systems where biological development is shaped not only by material well-being but also by environmental exposures, social norms, and access to inclusive opportunities. The Beyond GDP framework offers a comprehensive perspective on these interrelated influences [16]. While indicators of national well-being, such as the HDI, are increasingly utilised, there remains a critical gap in metrics that capture inclusion and sustainability, particularly regarding their relevance to adolescent-specific needs [16]. Building on our findings that suggest the diminishing role of the HDI over time and the growing influence of the GII and the ANS, this gap might hamper our ability to align adolescent health agendas with the broader aims of the SDGs, particularly SDG 3 (health and well-being), SDG 4 (quality education), SDG 5 (gender equality), SDG 10 (reduced inequalities), and SDG 13 (climate action) [50,51]. Now embodying the largest generation of young people in history, adolescents should be at the centre of development strategies that are equitable, sustainable, and inclusive. We therefore call for the adoption of multidimensional monitoring systems that move beyond GDP, enabling evidence-based policy and intersectoral actions to ensure adolescents thrive in safe, empowering, and future-oriented environments.

### Limitations

First, due to constraints in data availability, each dimension of the Beyond GDP framework was represented by a single indicator. Future research should incorporate a broader set of indicators to better reflect the complexity of adolescent health determinants. Second, our inclusion dimension did not account for disparities related to ethnic minorities, sexual and gender minorities, or other marginalised groups who may experience unique health challenges that are inadequately represented by national-level gender inequality measures. Third, the sustainability domain did not include indicators directly reflecting environmental stressors such as exposure to extreme weather events or climate-related displacement, which are increasingly relevant to adolescent well-being in a changing climate. Fourth, uncertainty arising from both outcome projections (GBD foresight) and predictor forecasts (HDI, GII, and ANS) were not formally propagated, which may have led to an underestimation of uncertainty in projected estimates. Fifth, potential conceptual overlap and endogeneity should be considered when interpreting the associations. The GII, used as a proxy for inclusion, includes health-related components and may therefore overlap with health outcomes. Similarly, there is conceptual overlap between the HDI and the SDI used in GBD projections, which may partly confound the respective associations of these measures with the adolescent health burden. These factors may influence the observed relationships; findings should therefore be interpreted as ecological associations rather than causal effects. However, our analysis found that the association between DALYs and HDI weakens substantially by 2035, suggesting that each index may capture different aspects of development over time. Future research could address this limitation by identifying additional indicators for each dimension or by developing an integrated measure that reduces redundancy while better reflecting the multifaceted determinants of adolescent health. Moreover, because the classification relies on predefined thresholds, countries close to the cut-off values may shift categories over time as disease patterns change. Also, although we accounted for regional clustering using fixed effects, residual confounding from unmeasured country-level factors (*e.g.* governance, conflict, or health system characteristics) cannot be fully excluded.

## CONCLUSIONS

This global ecological study underscores the critical importance of adopting a multidimensional framework encompassing well-being, inclusion, and sustainability to understand and address the adolescent health burden across 193 countries from 2010 to 2035. Based on our main analysis between 2010 and 2021 and a scenario analysis for 2035, progress in well-being (as reflected in the HDI) was associated with declining CMNN burdens in multi-burden countries. Inclusion (measured by the GII) showed a growing association with adolescent health across NCD-predominant and injury-excess countries. Furthermore, sustainability (measured by the ANS) appears to be a relevant factor in relatively developed groups, suggesting its potential to play a critical role in further alleviating the adolescent burden in the coming years. These findings highlight the urgent need for intersectoral, equity-driven policies that prioritise inclusive development and intergenerational equality, particularly in regions undergoing rapid social and epidemiological transitions [52]. For inclusive and sustainable well-being in the future, researchers should incorporate broader, intersectional indicators that account for structural inequalities and environmental threats to more effectively guide adolescent health investments and policy responses amid global uncertainty and climate change.

## Additional material


Online Supplementary Document

## References

[1] Baird S Choonara S Azzopardi PS Banati P Bessant J Biermann O A call to action: the second Lancet Commission on adolescent health and wellbeing. Lancet. 2025;405:1945–2022. 10.1016/S0140-6736(25)00503-3 40409329

[2] Baird S Das S Luckenbill S Oakley E Banati P Accelerating Well-being for Adolescents Through Transformative Public Policy: A Framework for Action. J Adolesc Health. 2024;75:S37–46. 10.1016/j.jadohealth.2024.03.013 39293876 PMC11825381

[3] Blum RW Astone NM Decker MR Mouli VC A conceptual framework for early adolescence: a platform for research. Int J Adolesc Med Health. 2014;26:321–31. 10.1515/ijamh-2013-0327 24486726 PMC4476282

[4] Pehlivanturk Kizilkan M O’Sullivan M Sabet F Rivkin LE Safitri Velies D Delgado-Zapata RM Advancing Adolescent Health: Empowering Future Leaders and Bridging Gaps in Research and Policy. J Adolesc Health. 2024;75:S3–5. 10.1016/j.jadohealth.2024.07.005 39293875

[5] Sherr L Cluver L Desmond C Toska E Aber L Dhaliwal M A new vehicle to accelerate the UN Sustainable Development Goals. Lancet Glob Health. 2020;8:e637–8. 10.1016/S2214-109X(20)30103-0 32353307 PMC7185933

[6] George A Jacobs T Ved R Jacobs T Rasanathan K Zaidi SA Adolescent health in the Sustainable Development Goal era: are we aligned for multisectoral action? BMJ Glob Health. 2021;6:e004448. 10.1136/bmjgh-2020-004448 33727279 PMC7970238

[7] Mohan A Kostelecky SM Sivakumar A Khalil M Clark H Improving adolescent wellbeing is an urgent global priority. BMJ. 2022;379:o2551. 10.1136/bmj.o2551 36302551 PMC9600263

[8] GBD 2017 Child and Adolescent Health Collaborators Reiner RC Olsen HE Ikeda CT Echko MM Ballestreros KE Diseases, Injuries, and Risk Factors in Child and Adolescent Health, 1990 to 2017: Findings From the Global Burden of Diseases, Injuries, and Risk Factors 2017 Study. JAMA Pediatr. 2019;173:e190337. 10.1001/jamapediatrics.2019.0337 31034019 PMC6547084

[9] Anderson NW Eisenberg D Halfon N Markowitz A Moore KA Zimmerman FJ Trends in Measures of Child and Adolescent Well-being in the US From 2000 to 2019. JAMA Netw Open. 2022;5:e2238582. 10.1001/jamanetworkopen.2022.38582 36287563 PMC9606848

[10] Xiao Y Mann JJ Chow JC-C Brown TT Snowden LR Yip PS-F Patterns of Social Determinants of Health and Child Mental Health, Cognition, and Physical Health. JAMA Pediatr. 2023;177:1294–305. 10.1001/jamapediatrics.2023.4218 37843837 PMC10580157

[11] Viner RM Ozer EM Denny S Marmot M Resnick M Fatusi A Adolescence and the social determinants of health. Lancet. 2012;379:1641–52. 10.1016/S0140-6736(12)60149-4 22538179

[12] Liu Y Zhong P Dang J Shi D Cai S Chen Z Trends of Cause-Specific Mortality and Association with Economic Status, Education Level, as Well as Health Investment among Adolescents Aged 10 to 24 Years in China, 2004–2019. Future. 2023;1:61–75. 10.3390/future1030008

[13] Hair NL Hanson JL Wolfe BL Pollak SD Association of Child Poverty, Brain Development, and Academic Achievement. JAMA Pediatr. 2015;169:822–9. 10.1001/jamapediatrics.2015.1475 26192216 PMC4687959

[14] Vallejo-Torres L Gonzalez Lopez-Valcarcel B Socioeconomic and contextual determinants of the burden of disease attributable to metabolic risks in childhood. Front Public Health. 2022;10:1003737. 10.3389/fpubh.2022.1003737 36424975 PMC9681493

[15] Beckwith S Chandra-Mouli V Blum RW Trends in Adolescent Health: Successes and Challenges From 2010 to the Present. J Adolesc Health. 2024;75:S9–19. 10.1016/j.jadohealth.2024.04.015 39293880

[16] Jansen A Wang R Behrens P Hoekstra R Beyond GDP: a review and conceptual framework for measuring sustainable and inclusive wellbeing. Lancet Planet Health. 2024;8:e695–705. 10.1016/S2542-5196(24)00147-5 39243785

[17] Patton GC Sawyer SM Santelli JS Ross DA Afifi R Allen NB Our future: a Lancet commission on adolescent health and wellbeing. Lancet. 2016;387:2423–78. 10.1016/S0140-6736(16)00579-1 27174304 PMC5832967

[18] Human Development Reports. Human Development Index. 2025. Available: https://hdr.undp.org/data-center/human-development-index. Accessed: 15 March 2025.

[19] Human Development Reports. Gender Inequality Index. 2025. Available: https://hdr.undp.org/data-center/thematic-composite-indices/gender-inequality-index#/indicies/GII. Accessed: 15 March 2025.

[20] Sawyer SM Azzopardi PS Wickremarathne D Patton GC The age of adolescence. Lancet Child Adolesc Health. 2018;2:223–8. 10.1016/S2352-4642(18)30022-1 30169257

[21] Rudan I Song P Adeloye D Campbell H Journal of global health’s guidelines for reporting analyses of big data repositories open to the public (GRABDROP): preventing “paper mills”, duplicate publications, misuse of statistical inference, and inappropriate use of artificial intelligence. J Glob Health. 2025;15:01004. 10.7189/jogh.15.01004 40587200 PMC12208284

[22] GBD 2015 Disease And Injury Incidence And Prevalence Collaborators Global, regional, and national incidence, prevalence, and years lived with disability for 310 diseases and injuries, 1990-2015: a systematic analysis for the global burden of disease study 2015. Lancet. 2016;388:1545–602. 10.1016/S0140-6736(16)31678-6 27733282 PMC5055577

[23] GBD 2021 Diseases and Injuries Collaborators Global incidence, prevalence, years lived with disability (YLDs), disability-adjusted life-years (DALYs), and healthy life expectancy (HALE) for 371 diseases and injuries in 204 countries and territories and 811 subnational locations, 1990-2021: a systematic analysis for the Global Burden of Disease Study 2021. Lancet. 2024;403:2133–61. 10.1016/S0140-6736(24)00757-8 38642570 PMC11122111

[24] GBD 2021 Forecasting Collaborators Burden of disease scenarios for 204 countries and territories, 2022-2050: a forecasting analysis for the Global Burden of Disease Study 2021. Lancet. 2024;403:2204–56. 10.1016/S0140-6736(24)00685-8 38762325 PMC11121021

[25] GBD 2019 Adolescent Mortality Collaborators Global, regional, and national mortality among young people aged 10-24 years, 1950-2019: a systematic analysis for the Global Burden of Disease Study 2019. Lancet. 2021;398:1593–618. 10.1016/S0140-6736(21)01546-4 34755628 PMC8576274

[26] Pezzey JCV Adjusted net savings needs further adjusting: Reassessing human and resource factors in sustainability measurement. J Environ Econ Manage. 2024;127:102984. 10.1016/j.jeem.2024.102984

[27] Martopullo I Neves PA Baird S Liang M Keats EC Cherkas A Understanding progress and challenges in women’s health and wellbeing in exemplar countries: a time-series study identifying positive outliers. Lancet Glob Health. 2024;12:e2012–23. 10.1016/S2214-109X(24)00364-4 39577974

[28] Patwardhan V Gil GF Arrieta A Cagney J DeGraw E Herbert ME Differences across the lifespan between females and males in the top 20 causes of disease burden globally: a systematic analysis of the Global Burden of Disease Study 2021. Lancet Public Health. 2024;9:e282–94. 10.1016/S2468-2667(24)00053-7 38702093 PMC11080072

[29] Hawkes S Sy EA Barker G Baum FE Buse K Chang AY Achieving gender justice for global health equity: the Lancet Commission on gender and global health. Lancet. 2025;405:1373–438. 10.1016/S0140-6736(25)00488-X 40209736

[30] Blum RW Boyden J Erulkar A Kabiru C Wilopo S Achieving Gender Equality Requires Placing Adolescents at the Center. J Adolesc Health. 2019;64:691–3. 10.1016/j.jadohealth.2019.02.002 31122503 PMC6531412

[31] Fleming PJ Lee JGL Dworkin SL “Real men don’t”: constructions of masculinity and inadvertent harm in public health interventions. Am J Public Health. 2014;104:1029–35. 10.2105/AJPH.2013.301820 24825202 PMC4062033

[32] Baird TL Buse K Food security, gender, and sexual and reproductive health and rights: a fragile golden thread. BMJ. 2022;379:o2599. 10.1136/bmj.o2599 36307134

[33] Xerxa Y White T Busa S Trasande L Hillegers MHJ Jaddoe VW Gender Diversity and Brain Morphology Among Adolescents. JAMA Netw Open. 2023;6:e2313139. 10.1001/jamanetworkopen.2023.13139 37171820 PMC10182431

[34] Cullen P Peden AE Francis KL Cini KI Azzopardi P Möller H Interpersonal Violence and Gender Inequality in Adolescents: A Systematic Analysis of Global Burden of Disease Data From 1990 to 2019. J Adolesc Health. 2024;74:232–45. 10.1016/j.jadohealth.2023.08.044 37988041

[35] Poteat VP Richburg A Day J Finch EK Calzo JP Marx RA LGBTQ+ advocacy and inclusive school policies are associated with self-worth among youth in gender-sexuality alliances over the school year. Dev Psychol. 2025;61:2106–18. 10.1037/dev0002024 40658586 PMC12577476

[36] Ioverno S Inclusive national educational policies as protective factors for LGBTI youth adjustment: an European cross-national study. J Adolesc Health. 2023;72:845–51. 10.1016/j.jadohealth.2023.01.013 36872119

[37] Khalil M Verite CK Choonara S Invest in youth led efforts for gender equality and pandemic preparedness. BMJ. 2023;383:2861. 10.1136/bmj.p2861 38052457 PMC10696548

[38] Chang AY Hawkes S Buse K Zarulli V Is ‘gender equality in health’ the right goal? Exploring issues of definition and measurement. BMJ Glob Health. 2025;10:e017900. 10.1136/bmjgh-2024-017900 40204464 PMC12056647

[39] Yan W Qin C Tao L Guo X Liu Q Du M Association between inequalities in human resources for health and all cause and cause specific mortality in 172 countries and territories, 1990-2019: observational study. BMJ. 2023;381:e073043. 10.1136/bmj-2022-073043 37164365 PMC10170610

[40] Tan JY San BJ Yeo YH Chan KH Shaaban HS Ezekwudo DE Social Vulnerability and Sickle Cell Disease Mortality in the US. JAMA Netw Open. 2024;7:e2440599. 10.1001/jamanetworkopen.2024.40599 39348116 PMC11443353

[41] Conteh L Engels T Molyneux DH Socioeconomic aspects of neglected tropical diseases. Lancet. 2010;375:239–47. 10.1016/S0140-6736(09)61422-7 20109925

[42] Emadi M Delavari S Bayati M Global socioeconomic inequality in the burden of communicable and non-communicable diseases and injuries: an analysis on global burden of disease study 2019. BMC Public Health. 2021;21:1771. 10.1186/s12889-021-11793-7 34583668 PMC8480106

[43] Cai S Wang H Zhang Y-H Zhao T-M Yuan X Deng H-W Could physical activity promote indicators of physical and psychological health among children and adolescents? An umbrella review of meta-analyses of randomized controlled trials. World J Pediatr. 2025;21:159–73. 10.1007/s12519-024-00874-3 39847308

[44] Kieling C Buchweitz C Caye A Silvani J Ameis SH Brunoni AR Worldwide Prevalence and Disability From Mental Disorders Across Childhood and Adolescence. JAMA Psychiatry. 2024;81:347–56. 10.1001/jamapsychiatry.2023.5051 38294785 PMC10831630

[45] Xie J Wang M Long Z Ning H Li J Cao Y Global burden of type 2 diabetes in adolescents and young adults, 1990-2019: systematic analysis of the Global Burden of Disease Study 2019. BMJ. 2022;379:e072385. 10.1136/bmj-2022-072385 36740855 PMC9727920

[46] Romanello M Di Napoli C Drummond P Green C Kennard H Lampard P The 2022 report of the Lancet Countdown on health and climate change: health at the mercy of fossil fuels. Lancet. 2022;400:1619–54. 10.1016/S0140-6736(22)01540-9 36306815 PMC7616806

[47] Anikeeva O Hansen A Varghese B Borg M Zhang Y Xiang J The impact of increasing temperatures due to climate change on infectious diseases. BMJ. 2024;387:e079343. 10.1136/bmj-2024-079343 39366706

[48] Lelieveld J Haines A Burnett R Tonne C Klingmüller K Münzel T Air pollution deaths attributable to fossil fuels: observational and modelling study. BMJ. 2023;383:e077784. 10.1136/bmj-2023-077784 38030155 PMC10686100

[49] Mugabe VA Gudo ES Inlamea OF Kitron U Ribeiro GS Natural disasters, population displacement and health emergencies: multiple public health threats in Mozambique. BMJ Glob Health. 2021;6:e006778. 10.1136/bmjgh-2021-006778 34489329 PMC8422305

[50] Wallengren E Guthold R Newby H Moller A-B Marsh AD Fagan L Relevance of the Sustainable Development Goals (SDGs) to Adolescent Health Measurement: A Systematic Mapping of the SDG Framework and Global Adolescent Health Indicators. J Adolesc Health. 2024;74:S47–55. 10.1016/j.jadohealth.2024.01.004 38762262

[51] GBD 2017 SDG Collaborators Measuring progress from 1990 to 2017 and projecting attainment to 2030 of the health-related Sustainable Development Goals for 195 countries and territories: a systematic analysis for the Global Burden of Disease Study 2017. Lancet. 2018;392:2091–138. 10.1016/S0140-6736(18)32281-5 30496107 PMC6227911

[52] Banati P Jones N Moreau C Mmari K Kågesten A Austrian K Intersectionality, gender norms, and young adolescents in context: a review of longitudinal multicountry research programmes to shape future action. Lancet Child Adolesc Health. 2024;8:522–31. 10.1016/S2352-4642(24)00079-8 38897717

