# Supplementary material for: Global associations of adolescent health with inclusive and sustainable well-being, 2010–2035
Source: J Glob Health. 2026 Jul 3;16:04188. doi: 10.7189/jogh.16.04188 (PMC13329882; doi:10.7189/jogh.16.04188)

Supplementary Materials

Table S1 Adherence to JoGH's GRABDROP guidelines items.

**JoGH guideline items**
1. Please list all papers published by each co-author in previous 3 years that were based on secondary analysis of a big data repository
[1] Armocida B, Sawyer SM, Monasta L, et al. Enhancing Global Burden of Disease Estimates With Collaborative Data Insights: A Case Study of Type 1 Diabetes in Finland. J Adolesc Health. 2026;78(4):662-668.
[2] He G, Liu Y, Bagga A, et al. Trends and socioeconomic inequality of the burden of congenital abnormalities of the kidney and urinary tract among children and adolescents. Nephrol Dial Transplant. 2025;40(3):484-494.
[3] Liu Y, Dong Y, Yan X, et al. Global trends and regional differences in non-transport unintentional injuries mortality among children and adolescents, 1990 to 2019: results from the Global Burden of Disease 2019 study. Chin Med J (Engl). 2022;135(17):2056-2065. Published 2022 Sep 5.
[4] Liu Y, Luo D, Zhong P, et al. Burden and risk factors of premature drowning mortality in 204 countries and territories, 1980-2021. Sci Rep. 2025;15(1):21036. Published 2025 Jul 1.
[5] Islam SM, Maddison R, Uddin R, Ball K, Livingstone KM, Khan A , Salmon J on behalf of the GBD Australia collaborator network. The burden and trend of diseases and their risk factors in Australia, 1990–2019: a systematic analysis for the Global Burden of Disease Study 2019. Lancet Public Health 2023;8(8):e585-99.
[6] Wang H, Song Y, Ma J, Ma S, Shen L, Huang Y, Thangaraju P, Basharat Z, Hu Y, Peden AE, Sawyer SM, Zhang H, Zou Z. Non-communicable diseases burden among adolescents and young adults aged 10–24 years in the South-East Asia and Western Pacific regions, 1990–2019: a systematic analysis of the Global Burden of Disease Study 2019. Lancet Child and Adolescent Health 2023;7(9): 621-35.
[7] Armocida B, Monasta L, Sawyer SM, Bustreo F, Onder G, Castelpietra G, Pricci F, Minardi V, Giacomozzi C, Abbafati C, Stafford LK, Pasovic M, Hay SI, Ong KL, Perel P, GBD 2019 Europe Adolescent Diabetes, Beran D. The burden of type 1 and type 2 diabetes among adolescents and young adults in 24 Western European countries, 1990-2019: results from the Global Burden of Disease Study 2019. International Journal of Public Health 2024 Feb 14th. https://doi.org/10.3389/ijph.2023.1606491
[8] Chen TJ, Dong B, Dong Y, Li J, MaY, Liu D, Zhang Y, Xing Y, Zheng Y, Luo X, Tao F, Ding Y, Hu P, Zou Z, Pan B, Tang P, Luo D, Liu Y, Li L, Li GN, Tian X, Huang X, Song Y, Ma J, Sawyer SM. Matching actions to needs: shifting policy responses to the changing health needs of Chinese children and adolescents. The Lancet 2024:403(10438), 1808 – 1820.
[9] Chen L, Wang L, Xing Y, Xie J, Su B, Geng M, Ren X, Zhang Y, Liu J, Ma T, Chen M, Miller J, Dong Y, Song Y, Ma J,

Sawyer S. Persistence and Variation of the Indirect Effects of COVID-19 Restrictions on the Spectrum of Notifiable Infectious Diseases in China: Analysis of National Surveillance Among Children and Adolescents From 2018 to 2021. JMIR Public Health Surveill 2024;10:e47626 (DOI: 10.2196/47626)

[10] Ng M, Gakidou E, Lo J, […GBD 2021 BMI Collaborators…], Murray, C.J.L., Sawyer, S., Vollset, SE. Global, regional and national prevalence of adult overweight and obesity, 1990-2021, with forecasts to 2050: a forecasting study for Global Burden of Disease 2021. The Lancet 2025;405(10481):813-838.

[11] Kerr, JA., […GBD 2021 BMI Collaborators…], Gakidou E*, Sawyer SM*, Azzopardi P*. (joint senior author). Global, regional and national prevalence of child and adolescent overweight and obesity, 1990-2021, with forecasts to 2050: a forecasting study for Global Burden of Disease Study 2021. The Lancet 2025 (DOI: 10.1016/S0140-6736(25)00397-6)

[12] Baird S, Choonara S, Azzopardi PS, Banati P, Bessant J, Biermann O, Capon A, Claeson M, Collins PY, De Wet-Billings N, Dogra S, Dong Y, Francis KL, Gebrekristos LT, Groves AK, Hay SI, Imbago-Jácome D, Jenkins AP, Kabiru CW, Kennedy EC, Li L, Lu C, Ma J, McGovern T, Mensa-Kwao A, Mojola SA, Nagata JM, Olumide AO, Omigbodun O, O'Sullivan M, Prost A, Requejo JH, Shawar YR, Shiffman J, Silverman A, Song Y, Swartz S, Tamambang R, Urdal H, Ward JL, Patton GC*, Sawyer SM*, Ezeh A*, Viner RM* (joint senior author). Lancet Commission on Adolescent Health and Wellbeing. The Lancet 2025

[13] Liu Y, Luo D, Zhong P, Dang J, Shi D, Cai S, Chen Z, Ma J, Zou Z, Song Y, Sawyer SM. Burden and risk factors of premature drowning mortality in 204 countries and territories, 1990-2021. Scientific Reports 2025;15(1):21036 (https://doi.org/10.1038/s41598-025-05418-x).

[14] Armocida B, Sawyer SM, Monasta L, Ong KL, Pasovic M, Xu Y, Tuomilehto J, Beran D. Enhancing Global Burden of Disease estimates with collaborative data insights: a case study of type 1 diabetes in Finland. J Adol Health 2026: Feb 6.

[15] Liu Y, Cai S, Yang R, Lin J, Dang J, Huang T, Li J, Zhu K, Chen Z, Zhang Y, Song Y, Sawyer SM. Global and regional prevalence, burden, and risk factors for MASLD in children and adolescents aged 5 to 24 years: a systematic review, meta-analysis, and modeling study. BMC Medicine 2026 (in press)

2. Please explain the key elements of your study design and the use of the available datasets that make your study an original scientific contribution

This study represents an original scientific contribution in several key aspects. First, while the GBD database has been widely used, our study uniquely integrates multiple Beyond GDP indicators including the Human Development Index (HDI), Gender Inequality Index (GII), and Adjusted Net Savings (ANS), to examine their associations with adolescent health burden across countries. Second, we focused specifically on individuals aged 10–24 years, a population that is often underrepresented in cross-national comparative analyses of health burden. Third, we applied a comprehensive analytical framework, including log-linear regression, non-linear

modelling, lagged analyses, and stratified analyses (by age group, sex, and region), to systematically evaluate robustness and potential heterogeneity. Finally, we incorporated scenario-based projections (2035) to explore potential future patterns, which further extends beyond conventional cross-sectional analyses using GBD data. Together, these elements distinguish our study from prior work and provide novel insights into the relationship between socioeconomic development and adolescent health outcomes.

3. Please list all publications that addressed similar research questions in the same dataset and indicate where you cited them in your paper

A previous study explored the association between socioeconomic and contextual determinants of the burden of disease attributable to metabolic risks in childhood using GBD 2019 data in 121 countries. We have cited this study in the Introduction section.

Vallejo-Torres L, Gonzalez Lopez-Valcarcel B. Socioeconomic and contextual determinants of the burden of disease attributable to metabolic risks in childhood. Front Public Health. 2022;10:1003737. Published 2022 Nov 8. doi:10.3389/fpubh.2022.1003737

4. Please explain how you addressed multiple testing through an appropriately rigorous statistical threshold and indicate this in the methods section

We acknowledge the issue of multiple testing given the number of models and subgroup analyses performed. We adopted several strategies to ensure robustness. First, effect estimates were interpreted based on both statistical significance and consistency across models, time points, and sensitivity analyses. Second, 95% confidence intervals were reported for all estimates to reflect the precision of associations. Third, key findings were evaluated for robustness across multiple analytical approaches, including age-stratified, sex-stratified, lagged, and region-adjusted models. These approaches reduce the likelihood that our conclusions are driven by spurious findings due to multiple comparisons. This rationale has been clarified in the Methods section.

5. Please declare to what extent have AI chatbots been used in developing your paper and to which parts of the paper did they contribute

The study design, data analysis, interpretation of results, and all scientific content were developed entirely by the authors. The ChatGPT was used for checking the grammar of the manuscript.

Table S2 All-cause age-standardized DALY rates of adolescents aged 10–24 years in 193 countries and territories in 1990, 2010, 2021, and 2035

Super region	Country/territory	Class	ASDR(95%UI)											
			1990			2010			2021			2035		
			Male	Female	Both	Male	Female	Both	Male	Female	Both	Male	Female	Both
Central Europe, Eastern Europe, and Central Asia	Albania	NCD predominant	12548.79(12450.36, 12647.82)	10101.20(10011.92, 10191.08)	11334.37(11267.84, 11401.20)	9938.50(9841.77,10035.98)	9036.90(8941.90,9132.69)	9503.65(9435.73,9571.95)	8654.01(8545.53,8763.61)	8920.29(8804.22,9037.60)	8780.37(8701.03,8860.30)	7292.41(7178.52,7407.71)	7671.21(7549.22,7794.73)	7473.86(7390.49,7557.97)
	Armenia	NCD predominant	11543.17(11443.19, 11643.80)	9819.37(9725.88,9913.54)	10687.32(10618.82, 10756.15)	10049.17(9949.39,10149.79)	8404.18(8310.75,8498.51)	9232.29(9163.87,9301.14)	9595.85(9481.82,9710.92)	8527.97(8414.59,8642.51)	9086.39(9005.82,9167.52)	8022.97(7914.41,8132.74)	7368.93(7258.39,7480.83)	7714.18(7636.60,7792.41)
	Azerbaijan	NCD predominant	13760.55(13690.44, 13830.93)	10852.52(10789.68, 10915.64)	12308.27(12261.20, 12355.48)	10746.94(10691.82, 10802.30)	9641.82(9588.38,9695.51)	10202.19(10163.77, 10240.73)	10433.49(10374.81, 10492.42)	9918.01(9857.60,9978.70)	10189.32(10147.20, 10231.56)	8577.10(8524.94,8629.52)	8430.00(8374.76,8485.54)	8508.48(8470.53,8546.56)
	Belarus	NCD predominant	14516.19(14445.59, 14587.05)	10132.32(10073.09, 10191.82)	12316.19(12270.13, 12362.38)	12343.15(12274.44, 12412.19)	8953.91(8892.21,9015.97)	10695.95(10649.60, 10742.47)	10045.10(9972.22,10118.39)	9418.37(9345.71,9491.47)	9741.36(9689.84,9793.08)	7812.95(7751.99,7874.31)	7564.78(7502.29,7627.70)	7692.11(7648.45,7735.98)
	Bosnia and Herzegovina	NCD predominant	11274.60(11190.05, 11359.65)	9247.91(9168.46,9327.90)	10301.82(10243.59, 10360.30)	9001.29(8908.81,9094.53)	8143.42(8052.75,8234.90)	8584.83(8519.96,8650.10)	8661.80(8553.47,8771.20)	8801.60(8689.43,8914.91)	8729.57(8651.57,8808.11)	7909.18(7788.14,8031.74)	7676.89(7553.70,7801.72)	7794.17(7707.75,7881.38)
	Bulgaria	NCD predominant	13719.47(13644.82, 13794.44)	10609.49(10542.43, 10676.88)	12197.02(12146.70, 12247.50)	11114.43(11031.61, 11197.77)	9596.06(9516.46,9676.23)	10375.89(10318.34, 10433.72)	11331.79(11238.51, 11425.64)	10142.95(10052.01, 10234.50)	10755.72(10690.46, 10821.27)	9167.08(9080.35,9254.46)	8145.78(8061.49,8230.78)	8669.63(8609.05,8730.55)

Super region	Country/territory	Class	ASDR(95%UI)											
			1990			2010			2021			2035		
			Male	Female	Both	Male	Female	Both	Male	Female	Both	Male	Female	Both
	Croatia	NCD predominant	11668.90(11577.45, 11760.91)	9184.65(9101.66,9268.22)	10448.72(10386.82, 10510.91)	9188.96(9095.74,9282.93)	8224.81(8134.30,8316.11)	8716.98(8651.92,8782.44)	8150.24(8055.19,8246.17)	8257.15(8158.75,8356.47)	8201.80(8133.37,8270.67)	7047.34(6946.68,7149.16)	7037.09(6933.55,7141.86)	7041.65(6969.40,7114.49)
	Czechia	NCD predominant	11594.52(11533.39, 11655.90)	9372.24(9316.23,9428.52)	10507.34(10465.78, 10549.03)	9380.53(9317.26,9444.16)	8442.10(8380.06,8504.52)	8922.89(8878.53,8967.43)	8555.42(8490.91,8620.31)	8611.00(8544.63,8677.77)	8582.53(8536.24,8629.00)	7818.27(7759.58,7877.30)	7416.74(7358.01,7475.83)	7622.13(7580.59,7663.85)
	Estonia	NCD predominant	17149.10(16952.76, 17347.18)	10890.78(10729.18, 11054.22)	14126.65(13998.49, 14255.70)	10668.32(10491.33, 10847.91)	8578.42(8411.42,8748.25)	9655.14(9533.04,9778.59)	9571.77(9382.94,9763.46)	9663.09(9468.45,9860.76)	9616.58(9480.84,9753.78)	7628.89(7468.37,7792.16)	8066.95(7896.88,8239.93)	7841.54(7724.58,7959.92)
	Georgia	NCD predominant	13229.33(13141.15, 13317.97)	9687.88(9611.62,9764.61)	11470.79(11412.42, 11529.39)	11980.63(11879.14, 12082.81)	8973.55(8882.42,9065.45)	10495.68(10427.35, 10564.38)	11609.78(11493.33, 11727.12)	9250.22(9139.81,9361.66)	10503.70(10422.98, 10584.90)	9831.31(9730.92,9932.54)	7756.61(7663.34,7850.80)	8834.90(8766.08,8904.16)
	Hungary	NCD predominant	12544.11(12479.73, 12608.74)	10143.76(10084.58, 10203.20)	11372.31(11328.47, 11416.28)	8782.37(8721.36,8843.72)	8495.85(8434.19,8557.88)	8642.48(8599.09,8686.05)	8674.56(8608.40,8741.12)	8869.89(8800.93,8939.26)	8769.67(8721.90,8817.65)	7288.17(7228.10,7348.64)	7521.93(7459.10,7585.17)	7401.91(7358.46,7445.57)
	Kazakhstan	NCD predominant	16384.83(16331.35, 16438.44)	12102.83(12056.11, 12149.70)	14281.55(14245.94, 14317.22)	14849.68(14799.26, 14900.24)	10990.97(10946.81, 11035.29)	12915.21(12881.70, 12948.79)	10992.54(10946.54, 11038.68)	9759.24(9715.46,9803.18)	10381.79(10350.02, 10413.65)	8978.88(8943.86,9014.00)	8234.96(8200.57,8269.46)	8615.06(8590.50,8639.67)
	Kyrgyzstan	NCD pred	15413.51(15317.38,	12309.75(12223.30,	13871.61(13806.89,	12786.41(12713.65,	10623.51(10556.21,	11712.52(11662.91,	10210.24(10143.77,	9867.22(9800	10040.67(9993.69,1	8278.74(8225	8357.01(8302	8316.31(8278.18,8

Super region	Country/territory	Class	ASDR(95%UI)												
			1990			2010			2021			2035			
			Male	Female	Both	Male	Female	Both	Male	Female	Both	Male	Female	Both	
		ominant	15510.11)	12396.66)	13936.56)	12859.49)	10691.16)	11762.29)	10277.05)	.88,9933.91)	0087.82)	.53,8332.21)	.37,8411.92)	354.58)	
	Latvia	NCD predominant	17488.72(17334.69,17643.82)	10772.72(10648.44,10898.12)	14217.27(14117.68,14317.42)	10627.44(10486.90,10769.66)	8665.77(8532.99,8800.36)	9667.53(9570.63,9765.31)	9600.17(9436.55,9765.94)	9072.66(8909.81,9237.78)	9345.35(9229.70,9462.11)	7538.51(7394.49,7684.84)	7531.47(7381.33,7684.11)	7532.96(7428.91,7638.22)	
	Lithuania	NCD predominant	15034.91(14918.79,15151.75)	10225.00(10126.79,10323.95)	12683.80(12607.41,12760.55)	12735.48(12614.61,12857.32)	8776.89(8672.58,8882.22)	10804.48(10724.29,10885.17)	10068.14(9934.87,10202.80)	9165.38(9034.30,9297.93)	9630.05(9536.41,9724.41)	8229.24(8099.38,8360.78)	7597.59(7469.10,7727.86)	7918.62(7827.15,8010.96)	
	Mongolia	Injury excess	15404.53(15274.65,15535.27)	13733.35(13610.01,13857.54)	14576.34(14486.69,14666.42)	15555.90(15436.40,15676.15)	11616.42(11511.88,11721.72)	13608.47(13528.91,13688.41)	12660.35(12546.69,12774.81)	10503.76(10398.78,10609.57)	11595.90(11518.40,11673.80)	11228.54(11145.36,11312.20)	9078.05(9001.36,9155.24)	10178.70(10121.97,10235.67)	
	Montenegro	NCD predominant	9651.51(9440.81,9865.78)	8814.81(8606.21,9027.24)	9252.13(9103.43,9402.67)	8997.27(8776.19,9222.65)	8360.09(8139.99,8584.81)	8688.83(8532.45,8847.42)	9138.53(8901.65,9380.28)	9348.09(9098.07,9603.42)	9237.85(9065.56,9412.66)	7833.00(7598.39,8073.23)	7629.16(7388.39,7876.01)	7734.86(7566.44,7906.20)	
	North Macedonia	NCD predominant	10317.70(10194.97,10441.54)	9629.97(9508.75,9752.36)	9981.46(9895.09,10068.40)	8734.54(8618.15,8852.20)	8193.95(8077.00,8312.27)	8473.55(8390.93,8556.82)	9304.14(9168.64,9441.22)	9435.14(9294.03,9577.95)	9367.14(9269.30,9465.80)	7386.46(7253.62,7521.24)	7447.78(7309.97,7587.68)	7416.15(7320.39,7512.93)	
	Poland	NCD predominant	12741.82(12707.90,12775.81)	9091.30(9062.17,9120.50)	10954.79(10932.36,10977.26)	9850.09(9818.90,9881.36)	8021.70(7992.58,8050.91)	8953.35(8931.98,8974.77)	9225.25(9190.87,9259.73)	8592.55(8558.49,8626.70)	8916.12(8891.90,8940.38)	7890.13(7857.90,7922.46)	7458.77(7426.40,7491.25)	7679.71(7656.86,7702.62)	

Super region	Country/territory	Class	ASDR(95%UI)											
			1990			2010			2021			2035		
			Male	Female	Both	Male	Female	Both	Male	Female	Both	Male	Female	Both
	Republic of Moldova	NCD predominant	15464.62(15355.79, 15574.05)	11073.70(10982.10, 11165.88)	13249.51(13178.48, 13320.84)	12009.47(11906.36, 12113.35)	9371.26(9276.41,9466.93)	10720.33(10650.08, 10790.98)	9784.03(9669.06,9900.05)	9264.28(9149.14,9380.53)	9530.62(9449.17,9612.61)	8018.40(7894.60,8143.81)	7691.63(7566.70,7818.26)	7855.29(7767.29,7944.12)
	Romania	NCD predominant	13184.47(13143.46, 13225.58)	10617.75(10580.35, 10655.25)	11920.25(11892.45, 11948.10)	10610.04(10564.82, 10655.41)	9216.63(9173.08,9260.34)	9933.37(9901.93,9964.90)	9905.26(9856.51,9954.19)	9155.21(9106.98,9203.63)	9541.09(9506.77,9575.51)	8357.39(8309.74,8405.25)	7797.09(7749.79,7844.62)	8084.00(8050.40,8117.71)
	Russian Federation	Injury excess	17024.27(17004.07, 17044.48)	10971.60(10955.08, 10988.13)	14055.85(14042.75, 14068.96)	15877.75(15857.61, 15897.92)	10787.16(10769.70, 10804.65)	13375.01(13361.64, 13388.38)	11419.02(11399.70, 11438.37)	10105.81(10087.27, 10124.37)	10776.50(10763.10, 10789.92)	9428.42(9412.02,9444.84)	8507.17(8490.94,8523.42)	8978.57(8967.03,8990.13)
	Serbia	NCD predominant	11627.94(11564.89, 11691.25)	9740.03(9680.97,9799.37)	10707.74(10664.44, 10751.17)	8357.19(8298.57,8416.14)	8015.20(7955.94,8074.81)	8190.13(8148.43,8232.00)	7577.03(7518.81,7635.59)	8117.61(8054.22,8181.39)	7835.58(7792.64,7878.70)	6567.64(6502.60,6633.23)	6993.40(6923.33,7064.07)	6771.31(6723.58,6819.32)
	Slovakia	NCD predominant	11536.48(11453.14, 11620.29)	9363.91(9287.65,9440.65)	10469.93(10413.31, 10526.78)	9332.47(9252.03,9413.49)	8335.31(8257.00,8414.24)	8844.02(8787.81,8900.53)	8818.39(8729.97,8907.49)	8825.25(8734.40,8916.82)	8821.66(8758.25,8885.42)	7599.12(7518.21,7680.70)	7612.77(7529.58,7696.68)	7605.66(7547.62,7664.05)
	Slovenia	NCD predominant	12300.44(12156.76, 12445.41)	9273.94(9147.04,9402.18)	10801.25(10705.21, 10897.96)	8964.77(8824.90,9106.47)	8025.16(7887.57,8164.71)	8511.56(8413.23,8610.84)	7962.83(7821.36,8106.26)	8177.03(8029.46,8326.68)	8067.01(7964.74,8170.29)	6985.12(6860.72,7111.32)	7062.62(6933.18,7193.97)	7022.87(6933.06,7113.62)
	Tajikistan	NCD pred	12683.06(12605.93,	12523.13(12446.48,	12611.41(12556.98,	11056.42(11000.41,	10314.18(10259.37,	10691.67(10652.46,	10795.68(10741.69,	10615.75(10560.72,	10708.47(10669.91,	9191.45(9148	8970.10(8926	9084.11(9053.28,9

Super region	Country/territory	Class	ASDR(95%UI)											
			1990			2010			2021			2035		
			Male	Female	Both	Male	Female	Both	Male	Female	Both	Male	Female	Both
		ominant	12760.55)	12600.14)	12666.02)	11112.65)	10369.21)	10731.00)	10849.89)	10671.01)	10747.14)	.27,9234.79)	.13,9014.23)	115.01)
	Turkmenistan	NCD predominant	13901.49(13804.78,13998.73)	12585.17(12492.46,12678.41)	13245.65(13178.63,13312.94)	13392.43(13309.80,13475.46)	12364.08(12280.03,12448.56)	12909.70(12850.70,12968.91)	13261.46(13176.36,13346.98)	12244.30(12158.45,12330.60)	12779.62(12719.10,12840.36)	11417.47(11342.77,11492.54)	10467.84(10394.55,10541.53)	10954.53(10902.14,11007.11)
	Ukraine	Injury excess	14863.50(14831.34,14895.72)	10502.32(10474.93,10529.75)	12705.15(12683.99,12726.34)	11584.85(11553.70,11616.08)	9115.02(9086.02,9144.09)	10378.73(10357.40,10400.09)	11884.45(11847.39,11921.60)	9729.50(9695.10,9764.00)	10835.75(10810.40,10861.15)	10431.13(10393.77,10468.60)	8447.47(8411.96,8483.11)	9465.99(9440.16,9491.87)
	Uzbekistan	NCD predominant	12983.94(12944.23,13023.75)	12439.83(12400.92,12478.83)	12713.32(12685.52,12741.18)	11148.56(11117.78,11179.42)	11255.51(11224.24,11286.84)	11202.35(11180.40,11224.32)	10377.04(10345.88,10408.27)	10976.68(10943.87,11009.57)	10670.21(10647.60,10692.86)	9045.32(9019.80,9070.90)	9764.71(9737.19,9792.29)	9391.58(9372.85,9410.34)
	Andorra	NCD predominant	10564.49(9788.41,11389.36)	10159.19(9358.77,11013.04)	10374.69(9814.09,10960.66)	9197.26(8473.22,9967.16)	9716.59(8938.66,10545.44)	9443.86(8910.65,10001.05)	9046.63(8348.14,9789.64)	10240.11(9467.39,11060.37)	9622.23(9099.95,10167.40)	7996.62(7194.56,8868.82)	9026.65(8154.47,9972.72)	8500.88(7904.95,9132.52)
High-income	Argentina	Injury excess	12578.30(12544.59,12612.08)	11069.24(11037.76,11100.78)	11820.07(11797.01,11843.16)	12816.60(12786.34,12846.91)	10675.93(10648.11,10703.81)	11749.54(11728.98,11770.13)	11644.97(11616.40,11673.58)	10809.00(10781.16,10836.90)	11228.86(11208.91,11248.84)	10817.02(10788.13,10845.99)	9707.41(9679.22,9735.67)	10272.02(10251.82,10292.26)
	Australia	NCD predominant	13451.42(13401.45,13501.53)	11781.48(11733.79,11829.32)	12627.32(12592.76,12661.96)	10303.01(10261.05,10345.11)	10913.45(10869.29,10957.74)	10601.55(10571.11,10632.06)	9879.16(9839.81,9918.62)	10616.59(10574.70,10658.60)	10237.97(10209.27,10266.74)	8993.72(8959.37,9028.17)	9916.90(9879.74,9954.17)	9441.85(9416.59,9467.16)

Super region	Country/territory	Class	ASDR(95%UI)											
			1990			2010			2021			2035		
			Male	Female	Both	Male	Female	Both	Male	Female	Both	Male	Female	Both
	Austria	NCD predominant	12379.90(12305.61, 12454.56)	10376.77(10306.31, 10447.64)	11399.25(11347.97, 11450.73)	10002.38(9931.50,10073.66)	9815.93(9743.88,9888.42)	9909.18(9858.64,9959.94)	9003.06(8935.22,9071.32)	9899.70(9825.41,9974.43)	9434.43(9384.24,9484.84)	7895.15(7832.40,7958.29)	8835.98(8767.43,8904.95)	8351.19(8304.80,8397.78)
	Belgium	NCD predominant	12134.73(12068.41, 12201.33)	10685.56(10621.60, 10749.82)	11425.26(11379.13, 11471.54)	9756.72(9695.35,9818.39)	10151.12(10087.53, 10215.03)	9949.31(9905.13,9993.64)	8858.34(8800.34,8916.63)	10162.81(10099.42, 10226.49)	9497.71(9454.80,9540.77)	7810.18(7757.12,7863.53)	9016.44(8958.02,9075.18)	8399.14(8359.74,8438.69)
	Brunei Darussalam	NCD predominant	15864.28(15475.82, 16260.15)	10496.93(10169.62, 10832.20)	13278.20(13022.00, 13538.22)	9111.34(8867.10,9360.77)	9045.42(8793.11,9303.29)	9082.21(8906.32,9260.77)	8022.29(7792.39,8257.64)	8573.16(8318.47,8834.03)	8277.66(8106.57,8451.64)	6936.22(6698.76,7180.10)	8091.72(7825.65,8364.72)	7492.89(7314.76,7674.35)
	Canada	NCD predominant	11990.44(11951.44, 12029.55)	10373.22(10336.22, 10410.31)	11193.61(11166.70, 11220.57)	9720.93(9687.61,9754.33)	9838.28(9804.04,9872.62)	9777.37(9753.49,9801.30)	9835.56(9802.20,9868.99)	10419.93(10384.44, 10455.51)	10119.15(10094.82, 10143.51)	9017.09(8987.30,9046.95)	9660.98(9629.17,9692.87)	9329.87(9308.11,9351.67)
	Chile	NCD predominant	12869.77(12818.93, 12920.77)	11214.97(11167.35, 11262.74)	12036.10(12001.28, 12070.99)	10954.66(10910.59, 10998.86)	10577.96(10533.83, 10622.24)	10766.88(10735.69, 10798.14)	10149.07(10105.54, 10192.75)	11025.30(10978.55, 11072.21)	10575.87(10543.97, 10607.85)	9023.99(8981.61,9066.54)	9828.62(9783.17,9874.25)	9418.20(9387.16,9449.34)
	Cyprus	NCD predominant	11023.17(10817.90, 11231.40)	9976.35(9773.11,10182.79)	10520.18(10375.37, 10666.51)	9395.62(9230.75,9562.97)	9520.35(9344.76,9698.63)	9462.40(9341.95,9584.15)	8566.61(8398.01,8737.83)	9505.56(9319.99,9693.99)	9017.83(8892.66,9144.36)	7653.86(7503.38,7806.68)	8694.84(8527.98,8864.23)	8157.03(8044.85,8270.43)
	Denmark	NCD pred	10369.84(10286.55,	10323.82(10238.38,	10348.02(10288.34,	8689.28(8609	9651.03(9565	9158.60(9100	8357.30(8280	9753.66(9668	9037.96(8980	7473.44(7400	8780.67(8699	8109.56(8055.04,8

Super region	Country/territory	Class	ASDR(95%UI)											
			1990			2010			2021			2035		
			Male	Female	Both	Male	Female	Both	Male	Female	Both	Male	Female	Both
		ominant	10453.67)	10409.83)	10407.97)	.78,8769.35)	.26,9737.38)	.18,9217.31)	.81,8434.33)	.78,9839.11)	.93,9095.27)	.47,7546.96)	.35,8862.58)	164.38)
	Finland	NCD predominant	13231.07(13130.89,13331.83)	10662.29(10570.20,10755.00)	11973.58(11905.37,12042.09)	10570.34(10480.53,10660.77)	10095.28(10005.26,10185.94)	10338.31(10274.68,10402.26)	10155.18(10064.70,10246.28)	10188.42(10095.39,10282.11)	10171.71(10106.81,10236.94)	9136.98(9048.22,9226.44)	9367.75(9275.45,9460.79)	9249.47(9185.44,9313.86)
	France	NCD predominant	12186.75(12160.14,12213.40)	11290.86(11264.62,11317.14)	11741.57(11722.89,11760.28)	9448.35(9423.93,9472.81)	10211.88(10186.01,10237.80)	9820.93(9803.16,9838.72)	8736.29(8713.16,8759.45)	10566.40(10540.42,10592.42)	9631.42(9614.07,9648.80)	7643.55(7620.93,7666.22)	9286.59(9261.06,9312.17)	8447.36(8430.34,8464.40)
	Germany	NCD predominant	11717.81(11693.64,11742.01)	10982.71(10958.53,11006.93)	11359.87(11342.77,11376.99)	9185.07(9162.31,9207.87)	10409.27(10384.44,10434.16)	9782.72(9765.91,9799.56)	8599.84(8577.65,8622.08)	10636.68(10610.67,10662.74)	9563.11(9546.13,9580.11)	7480.44(7460.06,7500.86)	9397.13(9373.64,9420.66)	8412.05(8396.55,8427.56)
	Greece	NCD predominant	11676.79(11616.07,11737.76)	10476.36(10417.60,10535.39)	11083.47(11041.19,11125.88)	10295.99(10231.51,10360.79)	10452.20(10384.48,10520.27)	10374.23(10327.49,10421.13)	9604.51(9536.39,9673.01)	10676.27(10603.08,10749.84)	10129.46(10079.51,10179.61)	8510.80(8439.35,8582.72)	9449.55(9372.35,9527.25)	8968.63(8916.09,9021.42)
	Iceland	NCD predominant	12245.27(11868.83,12630.90)	10313.48(9960.48,10676.07)	11296.58(11037.61,11560.25)	9044.99(8734.78,9363.76)	10096.05(9761.29,10439.73)	9560.24(9331.78,9793.06)	8928.59(8617.93,9247.75)	9941.86(9607.10,10285.44)	9419.60(9191.11,9652.41)	8097.45(7822.97,8379.24)	9339.34(9036.42,9650.00)	8701.33(8496.93,8909.48)
	Ireland	NCD predominant	11582.55(11487.07,11678.64)	10820.37(10726.26,10915.13)	11208.97(11141.87,11276.39)	10729.96(10635.51,10825.04)	10644.19(10549.04,10740.00)	10678.18(10611.17,10745.51)	9082.34(8998.67,9166.59)	10575.28(10483.39,10667.77)	9814.30(9752.23,9876.67)	8070.52(7994.99,8146.61)	9363.08(9279.41,9447.35)	8700.48(8644.22,8757.04)

Super region	Country/territory	Class	ASDR(95%UI)											
			1990			2010			2021			2035		
			Male	Female	Both	Male	Female	Both	Male	Female	Both	Male	Female	Both
	Israel	NCD predominant	10294.69(10218.79, 10371.03)	9807.22(9731.52,9883.37)	10056.37(10002.73, 10110.23)	8849.90(8790.45,8909.65)	9102.92(9041.36,9164.80)	8973.26(8930.47,9016.20)	8992.44(8937.59,9047.54)	9376.28(9319.00,9433.83)	9179.80(9140.16,9219.57)	7724.87(7678.91,7771.04)	8505.89(8456.39,8555.62)	8105.22(8071.49,8139.06)
	Italy	NCD predominant	11801.77(11775.50, 11828.09)	10669.84(10644.18, 10695.54)	11245.23(11226.85, 11263.63)	8885.31(8858.19,8912.51)	9950.11(9920.60,9979.69)	9403.46(9383.45,9423.50)	8247.36(8221.00,8273.78)	10265.81(10235.30, 10296.39)	9219.87(9199.80,9239.97)	7221.18(7193.58,7248.86)	9087.23(9055.38,9119.18)	8127.93(8106.93,8148.97)
	Japan	NCD predominant	9237.48(9221.86,9253.11)	8606.26(8590.81,8621.73)	8928.95(8917.96,8939.95)	7839.15(7821.69,7856.64)	8205.27(8186.96,8223.61)	8017.59(8004.95,8030.24)	7503.99(7486.22,7521.79)	8347.12(8327.91,8366.37)	7915.05(7901.98,7928.13)	6925.86(6907.41,6944.35)	7775.10(7755.05,7795.20)	7339.03(7325.43,7352.66)
	Luxembourg	NCD predominant	14449.09(14068.39, 14838.13)	10857.57(10518.29, 11205.65)	12684.67(12428.56, 12945.05)	9002.82(8732.00,9280.01)	9644.08(9357.59,9937.24)	9315.29(9117.96,9515.87)	8426.67(8187.70,8671.06)	10097.84(9827.23,10374.24)	9234.19(9053.97,9417.21)	7680.88(7480.69,7885.28)	9017.24(8793.59,9245.34)	8329.61(8179.67,8481.71)
	Malta	NCD predominant	9611.36(9315.39,9914.49)	9956.77(9647.11,10273.97)	9777.11(9562.68,9995.20)	9144.37(8859.54,9436.60)	9854.10(9548.47,10167.57)	9489.53(9280.49,9702.37)	9514.81(9183.86,9854.90)	9778.24(9433.71,10132.50)	9645.65(9406.28,9889.73)	8745.24(8465.03,9032.64)	9155.66(8859.56,9459.44)	8942.12(8738.10,9149.84)
	Monaco	NCD predominant	10623.77(9321.47,12071.93)	10434.93(9101.74,11927.39)	10523.54(9583.74,11538.66)	9786.42(8615.68,11073.24)	10442.65(9183.80,11826.83)	10101.91(9236.05,11027.66)	9799.20(8663.19,11048.73)	11030.55(9792.20,12387.51)	10400.74(9554.47,11304.61)	8772.33(7643.56,10027.71)	9816.48(8599.32,11168.21)	9282.07(8445.41,10183.35)
	Netherlands	NCD pred	9522.08(9475	10025.02(9976.21,1	9767.83(9734	8243.51(8198	9373.56(9324	8798.14(8764	8343.89(8298	10068.44(10017.56,	9187.10(9153	7377.04(7333	8820.73(8771	8080.76(8048.05,8

Super region	Country/territory	Class	ASDR(95%UI)											
			1990			2010			2021			2035		
			Male	Female	Both	Male	Female	Both	Male	Female	Both	Male	Female	Both
		ominant	.70,9568.65)	0074.04)	.18,9801.58)	.39,8288.82)	.54,9422.77)	.86,8831.51)	.60,8389.38)	10119.53)	.10,9221.19)	.41,7420.88)	.80,8869.87)	113.57)
	New Zealand	NCD predominant	16251.92(16132.42,16372.12)	12873.35(12765.30,12982.13)	14579.81(14499.13,14660.85)	11483.55(11388.15,11579.57)	11454.35(11357.59,11551.76)	11468.88(11400.91,11537.16)	9922.82(9838.38,10007.82)	11466.80(11372.53,11561.66)	10667.28(10604.17,10730.68)	9057.95(8981.59,9134.83)	10781.63(10695.79,10868.03)	9895.22(9837.91,9952.80)
	Norway	NCD predominant	11487.21(11391.52,11583.57)	10428.35(10334.54,10522.85)	10969.99(10902.89,11037.42)	9844.43(9756.62,9932.85)	10315.85(10223.63,10408.71)	10072.76(10009.12,10136.72)	8655.14(8574.90,8735.95)	10305.21(10214.83,10396.22)	9455.52(9395.24,9516.10)	7937.64(7861.01,8014.87)	9540.42(9453.70,9627.78)	8716.52(8658.80,8774.55)
	Portugal	NCD predominant	16115.05(16045.40,16184.92)	12281.91(12220.18,12343.88)	14220.75(14174.14,14267.49)	9618.74(9555.18,9682.62)	10865.05(10796.34,10934.11)	10231.01(10184.25,10277.95)	9256.59(9191.92,9321.63)	10931.17(10859.45,11003.27)	10077.05(10028.83,10125.46)	7671.01(7608.00,7734.42)	9263.60(9192.74,9334.89)	8448.96(8401.64,8496.49)
	Republic of Korea	NCD predominant	13320.16(13292.92,13347.45)	10820.67(10795.27,10846.11)	12106.89(12088.22,12125.58)	8179.12(8154.83,8203.47)	8410.81(8384.75,8436.93)	8288.45(8270.68,8306.26)	7408.35(7381.68,7435.11)	8175.86(8146.99,8204.83)	7778.58(7758.96,7798.24)	6671.03(6641.43,6700.74)	7542.61(7510.52,7574.82)	7096.74(7074.95,7118.60)
	San Marino	NCD predominant	10073.95(8940.08,11320.95)	9852.30(8725.63,11094.20)	9961.23(9155.12,10823.94)	8632.26(7506.49,9879.37)	9680.81(8474.69,11012.29)	9147.86(8315.64,10041.15)	8610.02(7560.32,9768.98)	10202.71(9024.39,11497.88)	9383.26(8589.35,10233.22)	7677.04(6537.63,8968.77)	9097.67(7797.41,10564.41)	8356.38(7486.11,9305.42)
	Singapore	NCD predominant	9224.20(9134.33,9314.79)	8986.48(8893.22,9080.57)	9107.82(9043.14,9172.88)	7272.19(7199.37,7345.64)	7636.55(7562.53,7711.21)	7453.02(7401.09,7505.26)	7366.32(7279.48,7453.98)	8099.25(8006.02,8193.32)	7716.75(7653.20,7780.72)	6785.87(6716.74,6855.56)	7452.51(7378.54,7527.08)	7111.52(7060.92,7162.39)

Super region	Country/territory	Class	ASDR(95%UI)											
			1990			2010			2021			2035		
			Male	Female	Both	Male	Female	Both	Male	Female	Both	Male	Female	Both
	Spain	NCD predominant	12846.94(12815.71,12878.23)	10697.17(10667.93,10726.47)	11791.21(11769.79,11812.66)	8283.13(8253.82,8312.53)	9611.42(9579.02,9643.90)	8930.91(8909.11,8952.76)	8229.45(8199.80,8259.18)	10351.59(10317.49,10385.78)	9263.78(9241.25,9286.35)	7117.31(7090.19,7144.52)	9172.69(9140.97,9204.49)	8114.96(8094.18,8135.79)
	Sweden	NCD predominant	9995.29(9928.83,10062.12)	10103.97(10035.18,10173.13)	10048.08(10000.25,10096.08)	8711.62(8651.10,8772.49)	9683.56(9617.84,9749.64)	9184.19(9139.59,9228.97)	8591.71(8531.94,8651.79)	10015.13(9948.06,10082.54)	9277.18(9232.40,9322.12)	7953.75(7901.29,8006.48)	9069.16(9011.31,9127.30)	8495.10(8456.13,8534.21)
	Switzerland	NCD predominant	13762.36(13676.14,13849.03)	11560.38(11478.17,11643.08)	12696.49(12636.78,12756.44)	9264.91(9194.35,9335.89)	10118.18(10042.75,10194.06)	9682.32(9630.72,9734.15)	8566.59(8498.70,8634.90)	10127.29(10050.75,10204.28)	9319.42(9268.42,9370.64)	7544.27(7483.70,7605.22)	9012.93(8944.77,9081.50)	8257.90(8212.42,8303.59)
	United Kingdom	NCD predominant	11627.44(11600.54,11654.38)	10755.93(10729.47,10782.44)	11197.35(11178.48,11216.25)	9350.56(9326.57,9374.60)	10179.50(10153.95,10205.11)	9756.32(9738.81,9773.86)	9371.33(9347.25,9395.46)	10535.23(10509.28,10561.23)	9943.19(9925.50,9960.89)	8049.83(8027.78,8071.92)	9248.48(9224.23,9272.78)	8634.68(8618.32,8651.06)
	United States of America	NCD predominant	14257.24(14243.50,14270.99)	11333.00(11320.42,11345.59)	12827.09(12817.76,12836.43)	12144.59(12132.86,12156.33)	11314.19(11302.57,11325.82)	11738.22(11729.96,11746.49)	14227.87(14215.17,14240.58)	13062.03(13049.56,13074.50)	13656.02(13647.12,13664.93)	11895.54(11883.63,11907.47)	11503.86(11491.81,11515.93)	11703.88(11695.41,11712.36)
	Uruguay	Injury excess	12115.29(12005.48,12225.87)	10749.35(10645.10,10854.37)	11436.62(11360.84,11512.79)	12421.78(12312.58,12531.71)	10191.51(10091.61,10292.18)	11311.70(11237.62,11386.15)	13255.24(13139.01,13372.28)	10968.39(10860.50,11077.13)	12120.84(12041.45,12200.64)	12292.58(12172.43,12413.72)	9703.97(9592.95,9816.04)	11026.07(10944.06,11108.59)
Latin America and	Antigua and Barbuda	NCD pred	11215.67(10523.04,	10785.26(10109.22,	10995.70(10509.74,	12197.99(11542.96,	10114.55(9527.43,1	11126.10(10685.79,	10671.39(10046.80,	10390.09(9760.33,1	10533.52(10088.04,	9549.95(8898.33,10	9500.05(8836.26,10	9524.44(9057.05,1

Super region	Country/territory	Class	ASDR(95%UI)											
			1990			2010			2021			2035		
			Male	Female	Both	Male	Female	Both	Male	Female	Both	Male	Female	Both
Caribbean		ominant	11942.28)	11495.21)	11498.56)	12880.81)	0728.58)	11579.93)	11326.27)	1051.40)	10994.40)	238.31)	202.18)	0010.51)
	Bahamas	Injury excess	14354.49(13989.56,14726.67)	12028.22(11693.74,12370.01)	13183.84(12935.99,13435.33)	16682.76(16309.03,17062.94)	12389.25(12069.57,12715.30)	14516.51(14270.53,14765.69)	15444.16(15099.30,15794.97)	12393.25(12086.68,12705.69)	13907.38(13676.47,14141.24)	14037.93(13669.16,14414.94)	11142.39(10803.58,11489.92)	12592.97(12342.04,12848.12)
	Barbados	NCD predominant	12760.32(12386.45,13142.89)	11756.03(11392.98,12128.02)	12259.40(11998.25,12524.96)	11317.45(10932.96,11712.19)	11255.12(10869.87,11650.82)	11286.63(11013.80,11564.61)	10241.06(9879.01,10613.40)	11173.86(10788.62,11569.84)	10699.00(10434.26,10969.00)	9116.39(8725.99,9520.69)	10200.71(9777.05,10638.96)	9644.78(9356.32,9940.33)
	Belize	Injury excess	13571.77(13159.93,13993.60)	12563.68(12167.69,12969.63)	13066.85(12780.56,13358.12)	16646.22(16296.30,17001.87)	12204.52(11908.17,12506.47)	14402.23(14172.99,14634.30)	14309.76(14022.76,14601.24)	11157.83(10906.05,11414.07)	12710.34(12519.52,12903.40)	13083.92(12809.89,13362.55)	10173.44(9926.35,10425.31)	11637.24(11452.34,11824.49)
	Bolivia (Plurinational State of)	Multi-burden	17651.12(17567.40,17735.15)	18872.12(18786.23,18958.32)	18277.94(18217.90,18338.14)	12484.18(12429.47,12539.07)	13666.25(13608.38,13724.30)	13070.03(13030.22,13109.93)	13050.84(12995.65,13106.21)	13467.59(13410.95,13524.42)	13257.77(13218.22,13297.40)	9949.25(9903.19,9995.47)	10233.76(10186.18,10281.50)	10088.93(10055.82,10122.12)
	Brazil	Injury excess	18118.46(18100.97,18135.96)	13533.76(13518.81,13548.73)	15800.06(15788.57,15811.55)	18080.26(18064.26,18096.28)	12944.63(12930.92,12958.36)	15522.00(15511.45,15532.55)	16340.75(16325.00,16356.51)	13013.06(12998.71,13027.43)	14690.39(14679.73,14701.06)	15381.74(15366.44,15397.05)	11518.69(11505.12,11532.27)	13484.54(13474.29,13494.79)
	Colombia	Injury excess	25033.05(24989.16,25077.00)	12606.50(12576.05,12637.02)	18638.18(18611.73,18664.66)	17230.48(17198.03,17262.97)	10674.13(10648.44,10699.86)	13958.37(13937.67,13979.09)	14744.75(14714.84,14774.71)	10599.22(10572.99,10625.49)	12722.55(12702.58,12742.53)	11937.47(11908.34,11966.66)	9437.91(9411.28,9464.61)	10714.14(10694.36,10733.95)

Super region	Country/territory	Class	ASDR(95%UI)											
			1990			2010			2021			2035		
			Male	Female	Both	Male	Female	Both	Male	Female	Both	Male	Female	Both
	Costa Rica	Injury excess	11061.12(10965.05, 11157.82)	9824.43(9733.71,9915.79)	10440.26(10374.17, 10506.67)	11671.59(11587.75, 11755.89)	9730.86(9654.05,9808.15)	10695.90(10639.04, 10752.99)	12608.46(12515.45, 12702.01)	10213.36(10129.28, 10297.98)	11398.68(11336.02, 11461.62)	11790.66(11695.80, 11886.15)	9172.08(9085.78,9259.05)	10502.22(10437.95,10566.81)
	Cuba	NCD predominant	13303.11(13246.87, 13359.55)	12910.70(12853.95, 12967.66)	13110.32(13070.36, 13150.38)	9189.53(9136.12,9243.18)	9524.62(9468.47,9581.03)	9351.70(9312.98,9390.55)	9692.14(9631.83,9752.74)	10127.04(10063.27, 10191.12)	9902.36(9858.51,9946.36)	7741.79(7682.63,7801.32)	8411.52(8347.75,8475.68)	8065.65(8022.22,8109.27)
	Dominica	Injury excess	13077.36(12431.08, 13749.08)	11249.94(10630.66, 11896.36)	12192.75(11742.65, 12655.94)	12675.93(11951.72, 13433.23)	11438.75(10744.94, 12166.04)	12061.01(11557.23, 12581.37)	13122.73(12365.23, 13915.42)	12264.61(11509.84, 13056.54)	12708.13(12170.72, 13263.55)	11950.46(11079.99, 12876.90)	11547.57(10676.58, 12476.53)	11751.91(11132.69,12399.43)
	Dominican Republic	Injury excess	14402.48(14333.13, 14472.09)	13006.43(12942.77, 13070.32)	13676.02(13629.06, 13723.10)	13849.20(13788.90, 13909.70)	11941.21(11884.91, 11997.71)	12899.36(12858.08, 12940.74)	14553.73(14492.01, 14615.66)	11489.15(11433.72, 11544.79)	13029.12(12987.61, 13070.74)	12637.67(12580.07, 12695.47)	10396.80(10343.69, 10450.12)	11533.59(11494.35,11572.92)
	Ecuador	Injury excess	16569.03(16505.53, 16632.71)	13695.52(13638.68, 13752.54)	15107.58(15065.05, 15150.22)	17746.78(17690.76, 17802.93)	12301.10(12254.52, 12347.80)	15014.88(14978.47, 15051.35)	13317.53(13272.15, 13363.04)	11416.99(11374.35, 11459.74)	12376.92(12345.76, 12408.15)	11309.12(11267.99, 11350.37)	9692.96(9654.02,9732.03)	10514.17(10485.81,10542.58)
	El Salvador	Injury excess	32014.07(31891.34, 32137.18)	15645.95(15563.89, 15728.35)	23491.10(23418.44, 23563.94)	18689.48(18600.23, 18779.06)	12216.51(12146.32, 12287.02)	15350.37(15294.03, 15406.88)	18778.85(18688.74, 18869.29)	11743.41(11671.86, 11815.30)	15212.94(15155.57, 15270.48)	15667.32(15580.90, 15754.12)	10273.88(10201.65, 10346.52)	13022.39(12965.85,13079.12)
	Grenada	NCD pred	14204.31(13560.10,	13414.64(12782.98,	13814.52(13361.70,	12256.56(11707.15,	11816.84(11273.90,	12037.98(11650.44,	10949.39(10397.13,	12019.20(11411.54,	11451.23(11040.82,	9965.39(9360.23,10	10785.10(10142.44,	10366.67(9923.77,

Super region	Country/territory	Class	ASDR(95%UI)											
			1990			2010			2021			2035		
			Male	Female	Both	Male	Female	Both	Male	Female	Both	Male	Female	Both
		ominant	14871.90)	14070.19)	14279.11)	12825.90)	12380.01)	12435.54)	11524.76)	12652.10)	11873.67)	600.40)	11458.93)	10824.77)
	Guatemala	Multi-burden	28321.51(28224.80,28418.48)	20388.49(20310.59,20466.63)	24183.38(24121.80,24245.07)	20722.73(20661.73,20783.88)	14264.45(14215.73,14313.31)	17372.59(17333.86,17411.40)	16716.31(16665.39,16767.35)	13627.73(13582.19,13673.38)	15134.99(15100.91,15169.12)	13914.16(13867.07,13961.37)	11352.86(11309.78,11396.07)	12638.97(12607.04,12670.96)
	Guyana	Multi-burden	20253.35(20006.57,20502.44)	16490.77(16271.80,16711.98)	18338.69(18173.90,18504.61)	20016.52(19751.33,20284.53)	15649.31(15416.79,15884.58)	17819.15(17642.82,17996.87)	18988.25(18729.28,19250.01)	15774.80(15536.03,16016.44)	17391.93(17215.53,17569.75)	16976.28(16699.12,17256.97)	13389.50(13143.00,13639.53)	15172.03(14986.52,15359.29)
	Haiti	Multi-burden	23659.04(23559.02,23759.40)	29646.60(29538.40,29755.10)	26802.01(26727.94,26876.24)	79294.96(79157.87,79432.22)	204411.44(204194.03,204629.03)	142710.06(142580.81,142839.39)	19969.61(19905.43,20033.94)	24744.63(24673.35,24816.08)	22366.93(22318.93,22415.00)	20223.84(20163.64,20284.18)	21725.22(21662.44,21788.14)	20966.32(20922.84,21009.87)
	Honduras	Injury excess	18830.83(18728.47,18933.64)	17751.03(17654.99,17847.49)	18252.03(18182.04,18322.23)	14376.04(14311.01,14441.30)	13724.71(13662.58,13787.07)	14032.85(13987.94,14077.86)	13182.07(13125.70,13238.63)	12779.60(12724.46,12834.93)	12976.32(12936.90,13015.83)	11426.81(11375.42,11478.38)	11070.69(11019.08,11122.49)	11251.84(11215.41,11288.36)
	Jamaica	NCD predominant	9874.84(9774.66,9975.79)	10717.84(10614.64,10821.80)	10301.90(10229.91,10374.28)	9471.76(9378.06,9566.17)	10546.84(10447.01,10647.39)	10002.62(9934.17,10071.43)	10644.94(10539.66,10751.05)	10998.15(10889.07,11108.09)	10818.41(10742.60,10894.66)	8533.00(8420.08,8647.12)	9750.85(9628.54,9874.39)	9134.12(9050.89,9217.96)
	Mexico	Injury excess	15711.95(15691.16,15732.77)	11548.31(11530.88,11565.77)	13579.68(13566.17,13593.21)	14225.44(14207.11,14243.78)	10706.70(10690.91,10722.50)	12441.05(12428.98,12453.12)	14643.28(14625.00,14661.58)	11188.41(11172.34,11204.49)	12912.90(12900.74,12925.08)	12744.50(12727.01,12762.01)	9757.80(9742.17,9773.44)	11266.79(11255.05,11278.55)

Super region	Country/territory	Class	ASDR(95%UI)											
			1990			2010			2021			2035		
			Male	Female	Both	Male	Female	Both	Male	Female	Both	Male	Female	Both
	Nicaragua	NCD predominant	15944.20(15843.18, 16045.74)	12706.52(12619.85, 12793.66)	14257.86(14191.68, 14324.28)	11438.08(11370.33, 11506.15)	10593.46(10527.79, 10659.45)	11013.06(10965.88, 11060.40)	10914.64(10848.75, 10980.82)	10701.50(10634.97, 10768.35)	10807.26(10760.43, 10854.23)	9315.52(9254.54,9376.81)	9107.29(9045.33,9169.58)	9213.19(9169.72,9256.83)
	Panama	Injury excess	13548.02(13431.26, 13665.56)	11463.68(11355.39, 11572.75)	12510.41(12430.71, 12590.50)	15594.56(15481.99, 15707.76)	10760.87(10666.47, 10855.90)	13201.94(13128.31, 13275.88)	12416.62(12325.23, 12508.52)	10358.02(10272.36, 10444.21)	11411.28(11348.51, 11474.32)	11194.15(11110.84, 11277.96)	9400.21(9321.34,9479.60)	10319.36(10261.87,10377.11)
	Paraguay	Injury excess	11735.20(11648.68, 11822.23)	12457.85(12368.77, 12547.42)	12096.04(12033.92, 12158.41)	14269.11(14194.56, 14343.96)	12302.20(12231.81, 12372.88)	13300.45(13249.12, 13351.93)	13764.34(13692.53, 13836.44)	12939.11(12867.97, 13010.54)	13358.78(13308.21, 13409.51)	12581.26(12510.54, 12652.29)	11177.82(11109.31, 11246.65)	11892.84(11843.56,11942.29)
	Peru	NCD predominant	19560.66(19513.95, 19607.46)	14899.90(14859.54, 14940.35)	17203.81(17172.99, 17234.68)	11371.57(11339.48, 11403.72)	10776.68(10745.03, 10808.40)	11074.21(11051.68, 11096.78)	11385.86(11355.87, 11415.91)	11480.51(11448.87, 11512.23)	11428.03(11406.27, 11449.83)	9074.76(9047.79,9101.79)	9203.18(9175.14,9231.28)	9138.52(9119.08,9158.00)
	Saint Kitts and Nevis	Injury excess	12545.74(11688.82, 13449.50)	12612.40(11748.64, 13523.53)	12578.98(11967.50, 13213.93)	12811.13(11985.42, 13679.90)	10817.66(10061.47, 11616.83)	11810.08(11247.64, 12393.95)	12887.46(12009.67, 13816.59)	10917.82(10107.44, 11780.15)	11900.01(11299.59, 12526.11)	13273.78(12248.85, 14365.91)	10152.53(9242.98,11131.85)	11726.03(11035.85,12450.22)
	Saint Lucia	Injury excess	14201.01(13708.57, 14706.89)	12321.89(11869.26, 12787.57)	13247.81(12912.89, 13589.34)	14010.10(13532.33, 14500.81)	12017.46(11569.90, 12478.25)	13019.15(12690.88, 13353.96)	13465.02(12949.25, 13997.20)	11702.54(11206.04, 12216.46)	12605.08(12245.62, 12972.96)	12029.02(11480.52, 12598.54)	10570.30(10039.88, 11122.98)	11314.76(10931.55,11708.75)
	Saint Vincent and the	Injury	12606.62(12102.65,	13371.79(12845.06,	12984.96(12619.55,	13726.64(13142.07,	12356.44(11791.76,	13052.23(12644.25,	13552.23(12944.83,	12915.14(12303.75,	13247.86(12815.22,	12695.04(12025.98,	11831.08(11170.03,	12264.02(11791.90

Super region	Country/territory	Class	ASDR(95%UI)											
			1990			2010			2021			2035		
			Male	Female	Both	Male	Female	Both	Male	Female	Both	Male	Female	Both
	Grenadines	excess	13126.65)	13915.06)	13358.49)	14330.81)	12941.45)	13470.15)	14181.23)	13549.34)	13691.56)	13394.50)	12523.90)	,12751.60)
	Suriname	Multi-burden	16185.70(15875.13,16500.96)	16397.74(16073.95,16726.47)	16306.43(16081.74,16533.52)	15141.74(14859.69,15427.84)	14324.88(14048.36,14605.51)	14716.59(14519.07,14916.15)	14456.47(14181.53,14735.40)	15238.11(14949.19,15531.23)	14825.34(14626.02,15026.71)	12759.65(12500.82,13022.67)	13394.15(13120.01,13672.75)	13071.36(12882.75,13262.13)
	Trinidad and Tobago	Injury excess	15337.40(15152.24,15524.29)	13163.73(12991.07,13338.13)	14256.62(14129.87,14384.23)	17523.11(17324.02,17724.09)	11893.67(11725.18,12064.13)	14727.43(14596.75,14859.07)	17227.54(17009.98,17447.19)	12493.35(12305.18,12683.69)	14898.27(14753.96,15043.65)	15270.03(15044.96,15497.87)	10580.73(10387.44,10776.98)	12938.98(12790.35,13089.05)
	Venezuela (Bolivarian Republic of)	Injury excess	16611.45(16564.99,16658.01)	11852.55(11813.36,11891.83)	14229.04(14198.65,14259.48)	23418.74(23371.75,23465.81)	11073.55(11040.74,11106.45)	17278.94(17250.22,17307.69)	27074.98(27012.74,27137.33)	12431.27(12390.98,12471.66)	19456.24(19419.81,19492.73)	27463.03(27403.91,27522.24)	12810.50(12769.36,12851.74)	20176.59(20140.51,20212.72)
North Africa and Middle East	Afghanistan	Multi-burden	31853.43(31763.25,31943.81)	30016.20(29932.98,30099.61)	30893.84(30832.72,30955.05)	30674.49(30615.54,30733.54)	24892.81(24838.11,24947.61)	27873.19(27832.86,27913.57)	49400.05(49340.33,49459.83)	26443.29(26398.51,26488.13)	38224.65(38187.04,38262.28)	16834.10(16805.43,16862.81)	17533.82(17503.49,17564.18)	17169.41(17148.57,17190.27)
	Algeria	NCD predominant	15518.23(15480.89,15555.64)	14079.28(14043.16,14115.47)	14812.97(14786.97,14839.01)	11314.16(11285.94,11342.45)	11163.49(11134.99,11192.05)	11239.49(11219.43,11259.58)	10743.82(10715.36,10772.34)	11061.62(11032.15,11091.15)	10899.04(10878.56,10919.55)	9171.66(9149.11,9194.26)	9782.22(9758.24,9806.24)	9467.84(9451.40,9484.30)
	Bahrain	NCD predominant	12452.87(12184.11,12726.35)	13016.53(12733.66,13304.26)	12710.70(12515.86,12907.93)	11304.83(11130.04,11481.86)	10563.54(10381.85,10747.74)	10963.62(10837.78,11090.63)	10133.20(9991.72,10276.35)	10989.90(10819.71,11162.13)	10494.81(10386.17,10604.36)	9104.40(8963.50,9247.17)	9647.33(9492.14,9804.57)	9362.25(9257.79,9467.69)

Super region	Country/territory	Class	ASDR(95%UI)											
			1990			2010			2021			2035		
			Male	Female	Both	Male	Female	Both	Male	Female	Both	Male	Female	Both
	Egypt	NCD predominant	15724.30(15698.16, 15750.48)	15856.41(15829.18, 15883.67)	15789.80(15770.94, 15808.68)	12898.49(12879.11, 12917.90)	11592.09(11573.27, 11610.93)	12261.78(12248.25, 12275.31)	12164.05(12146.53, 12181.58)	11645.55(11627.81, 11663.31)	11914.04(11901.57, 11926.52)	10426.63(10412.36, 10440.91)	9779.78(9765.56,9794.01)	10112.84(10102.76,10122.92)
	Iran (Islamic Republic of)	Injury excess	20362.97(20333.66, 20392.32)	20248.65(20219.14, 20278.19)	20302.01(20281.22, 20322.82)	15104.71(15081.99, 15127.46)	12707.44(12686.27, 12728.65)	13917.00(13901.45, 13932.55)	13525.42(13501.33, 13549.54)	12444.99(12421.36, 12468.66)	12997.50(12980.61, 13014.40)	12054.55(12033.19, 12075.93)	10951.67(10930.58, 10972.80)	11516.78(11501.76,11531.81)
	Iraq	Injury excess	22157.04(22104.56, 22209.62)	14780.46(14736.02, 14825.01)	18629.63(18594.96, 18664.35)	20403.73(20363.77, 20443.74)	14122.55(14088.46, 14156.71)	17352.84(17326.45, 17379.27)	13595.61(13567.10, 13624.16)	11985.96(11958.31, 12013.66)	12818.18(12798.29, 12838.10)	15108.57(15079.93, 15137.24)	11827.10(11800.91, 11853.32)	13510.35(13490.90,13529.83)
	Jordan	NCD predominant	11685.80(11606.69, 11765.31)	12957.74(12868.93, 13047.03)	12275.37(12216.24, 12334.72)	9760.94(9705.83,9816.29)	10228.78(10169.18, 10288.65)	9978.07(9937.60,10018.66)	9405.86(9363.34,9448.53)	10508.27(10460.81, 10555.90)	9923.13(9891.42,9954.91)	8122.49(8083.36,8161.78)	9134.57(9091.93,9177.36)	8614.31(8585.43,8643.28)
	Kuwait	NCD predominant	31337.08(31106.86, 31568.64)	13379.36(13228.71, 13531.35)	22466.46(22328.55, 22605.03)	9634.30(9528.78,9740.76)	9290.12(9182.76,9398.50)	9468.30(9392.97,9544.12)	9191.78(9101.24,9283.02)	9114.78(9022.16,9208.14)	9154.44(9089.65,9219.59)	8450.33(8372.34,8528.90)	8467.51(8387.15,8548.50)	8458.62(8402.61,8514.92)
	Lebanon	NCD predominant	29138.37(28977.79, 29299.64)	15019.10(14902.69, 15136.20)	22127.23(22027.87, 22226.93)	10279.38(10197.07, 10362.20)	10388.31(10301.34, 10475.85)	10330.42(10270.62, 10390.50)	11164.74(11081.98, 11247.97)	11491.89(11402.88, 11581.43)	11318.86(11258.21, 11379.75)	9399.85(9324.22,9475.96)	9536.73(9457.15,9616.85)	9465.75(9410.89,9520.86)
	Libya	Injury	14062.95(13976.51,	12552.18(12469.37,	13323.53(13263.59,	14135.30(14059.56,	12296.30(12223.23,	13247.48(13194.75,	14794.53(14715.20,	12842.55(12767.10,	13835.37(13780.55,	15407.53(15314.73,	12452.25(12366.28,	13958.18(13894.80

Super region	Country/territory	Class	ASDR(95%UI)											
			1990			2010			2021			2035		
			Male	Female	Both	Male	Female	Both	Male	Female	Both	Male	Female	Both
		excess	14149.80)	12635.44)	13383.69)	14211.36)	12369.71)	13300.38)	14874.19)	12918.34)	13890.35)	15500.79)	12538.71)	,14021.80)
	Morocco	NCD predominant	14572.38(14534.92,14609.91)	15674.24(15635.59,15712.96)	15129.14(15102.21,15156.10)	11901.66(11870.86,11932.53)	12794.74(12762.75,12826.79)	12349.14(12326.94,12371.38)	11170.97(11140.98,11201.03)	11711.61(11680.36,11742.92)	11436.90(11415.26,11458.58)	9336.06(9308.77,9363.40)	10071.21(10042.19,10100.29)	9695.10(9675.21,9715.03)
	Oman	NCD predominant	16894.33(16745.67,17044.02)	12323.90(12178.92,12470.27)	14896.37(14791.56,15001.75)	13762.81(13655.29,13871.03)	10557.43(10455.25,10660.40)	12316.60(12241.97,12391.60)	11021.08(10928.12,11114.68)	10666.84(10568.55,10765.83)	10865.55(10798.06,10933.36)	10030.16(9956.64,10104.12)	9731.91(9655.96,9808.33)	9892.01(9839.15,9945.10)
	Palestine	Injury excess	19890.34(19740.25,20041.31)	12745.84(12623.34,12869.27)	16395.35(16297.98,16493.17)	15123.45(15032.91,15214.40)	10792.63(10714.72,10870.97)	12995.04(12935.14,13055.16)	12758.79(12682.37,12835.56)	11004.08(10931.40,11077.13)	11903.18(11850.34,11956.20)	11230.45(11162.03,11299.19)	9899.57(9833.69,9965.80)	10578.92(10531.36,10626.64)
	Qatar	NCD predominant	15047.37(14729.07,15371.16)	11246.16(10910.47,11589.84)	13449.07(13219.63,13681.64)	14478.38(14279.40,14679.76)	9858.10(9662.56,10056.80)	12623.70(12485.80,12762.88)	10309.18(10176.77,10443.08)	9319.42(9171.95,9468.71)	9797.29(9702.09,9893.26)	9265.28(9174.86,9356.47)	8611.39(8513.93,8709.73)	8951.92(8886.07,9018.19)
	Saudi Arabia	Injury excess	15236.86(15190.45,15283.37)	15190.14(15139.24,15241.17)	15209.94(15175.68,15244.25)	12911.95(12879.16,12944.81)	11713.86(11680.02,11747.78)	12368.99(12345.40,12392.61)	11474.21(11443.64,11504.85)	11097.59(11063.78,11131.48)	11319.04(11296.34,11341.77)	11126.91(11095.60,11158.30)	10436.81(10404.18,10469.53)	10825.26(10802.63,10847.94)
	Sudan	Multi-burden	26413.53(26356.42,26470.75)	21921.33(21869.76,21972.99)	24154.55(24116.12,24193.04)	15913.51(15880.11,15946.97)	16289.76(16255.74,16323.82)	16105.93(16082.09,16129.79)	14129.38(14102.11,14156.69)	14524.67(14496.16,14553.23)	14321.51(14301.80,14341.25)	12713.26(12689.45,12737.12)	12931.15(12906.41,12955.92)	12813.49(12796.34,12830.67)

Super region	Country/territory	Class	ASDR(95%UI)											
			1990			2010			2021			2035		
			Male	Female	Both	Male	Female	Both	Male	Female	Both	Male	Female	Both
	Syrian Arab Republic	Injury excess	14656.32(14605.10, 14707.69)	14393.57(14341.67, 14445.61)	14528.67(14492.20, 14565.21)	11331.61(11297.54, 11365.76)	11210.51(11175.30, 11245.79)	11271.95(11247.46, 11296.47)	16654.62(16599.33, 16710.05)	12848.80(12803.58, 12894.15)	14585.09(14550.03, 14620.23)	27279.13(27199.87, 27358.56)	16204.87(16141.64, 16268.30)	21805.76(21754.92,21856.70)
	Tunisia	NCD predominant	12268.03(12209.00, 12327.28)	11697.44(11638.92, 11756.17)	11989.10(11947.51, 12030.80)	10607.56(10554.65, 10660.69)	10404.00(10350.92, 10457.29)	10504.55(10467.08, 10542.13)	11273.15(11214.94, 11331.60)	11483.50(11423.51, 11543.72)	11373.02(11331.24, 11414.91)	9323.70(9272.47,9375.16)	9521.70(9467.75,9575.90)	9418.78(9381.62,9456.07)
	Turkey	NCD predominant	15207.81(15182.70, 15232.95)	12835.10(12811.58, 12858.65)	14044.24(14027.02, 14061.49)	10859.06(10838.57, 10879.57)	9844.37(9824.37,9864.39)	10364.31(10349.99, 10378.66)	10416.74(10396.68, 10436.83)	10564.11(10543.39, 10584.87)	10488.56(10474.15, 10502.99)	9367.61(9347.24,9388.02)	9206.25(9185.48,9227.05)	9288.51(9273.96,9303.07)
	United Arab Emirates	NCD predominant	15490.93(15329.28, 15653.93)	12489.64(12326.41, 12654.56)	14130.68(14015.94, 14246.15)	11627.32(11550.07, 11705.02)	10750.94(10656.81, 10845.76)	11286.78(11228.49, 11345.32)	11865.90(11776.82, 11955.50)	10245.21(10159.34, 10331.63)	11097.91(11035.82, 11160.26)	10847.20(10788.63, 10906.06)	9328.35(9264.70,9392.38)	10317.58(10274.20,10361.13)
	Yemen	Multi-burden	20462.00(20398.97, 20525.19)	22502.13(22434.31, 22570.11)	21476.70(21430.44, 21523.05)	15546.71(15508.23, 15585.27)	17692.73(17650.84, 17734.70)	16600.29(16571.88, 16628.74)	30620.10(30572.79, 30667.47)	19229.65(19191.94, 19267.42)	24986.72(24956.40, 25017.07)	15467.20(15438.42, 15496.02)	15860.43(15830.52, 15890.38)	15654.73(15633.99,15675.48)
South Asia	Bangladesh	Multi-burden	20234.46(20213.07, 20255.86)	21639.08(21617.24, 21660.94)	20982.16(20966.84, 20997.48)	14294.85(14279.08, 14310.64)	13889.90(13874.57, 13905.24)	14113.79(14102.78, 14124.81)	11555.00(11540.86, 11569.16)	12448.66(12434.46, 12462.87)	12031.19(12021.16, 12041.24)	10213.40(10199.80, 10227.01)	10707.97(10693.80, 10722.15)	10458.07(10448.25,10467.89)
	Bhutan	Multi-	16917.26(16688.22,	21025.66(20746.27,	18776.52(18598.32,	11736.03(11542.99,	13275.73(13062.09,	12471.05(12327.49,	10406.12(10212.31,	10928.77(10725.76,	10661.14(10520.76,	9782.53(9580	9967.56(9761.48,10	9873.88(9729.46,1

Super region	Country/territory	Class	ASDR(95%UI)											
			1990			2010			2021			2035		
			Male	Female	Both	Male	Female	Both	Male	Female	Both	Male	Female	Both
		burden	17148.69)	21307.95)	18956.03)	11931.59)	13492.02)	12615.91)	10602.84)	11134.76)	10802.98)	.69,9987.64)	176.98)	0019.94)
	India	Multi-burden	19495.71(19488.20, 19503.22)	24032.85(24024.16, 24041.54)	21676.23(21670.51, 21681.94)	15273.14(15267.64, 15278.64)	17166.45(17160.35, 17172.56)	16174.36(16170.27, 16178.45)	12207.45(12202.71, 12212.20)	14014.07(14008.78, 14019.36)	13074.19(13070.65, 13077.73)	10196.65(10192.00, 10201.31)	11462.13(11456.94, 11467.32)	10799.36(10795.89,10802.84)
	Nepal	Multi-burden	19829.17(19777.38, 19881.06)	23785.88(23730.21, 23841.65)	21869.17(21831.06, 21907.33)	13694.68(13659.31, 13730.13)	15191.13(15155.53, 15226.79)	14468.23(14443.13, 14493.36)	12921.09(12888.50, 12953.74)	13089.37(13057.25, 13121.55)	13002.65(12979.79, 13025.55)	10609.27(10578.88, 10639.71)	10719.78(10688.41, 10751.22)	10661.71(10639.88,10683.57)
	Pakistan	Multi-burden	16104.07(16085.62, 16122.54)	20032.66(20010.97, 20054.36)	17974.15(17960.00, 17988.30)	17109.52(17094.55, 17124.50)	19453.53(19437.22, 19469.85)	18258.38(18247.33, 18269.44)	14378.42(14366.24, 14390.60)	17434.20(17420.31, 17448.08)	15855.62(15846.42, 15864.82)	11835.92(11825.53, 11846.33)	14299.11(14287.32, 14310.90)	13027.72(13019.89,13035.56)
Southeast Asia, East Asia, and Oceania	Cambodia	Multi-burden	24026.47(23947.87, 24105.28)	22293.66(22220.69, 22366.83)	23130.36(23076.84, 23183.98)	15769.37(15719.44, 15819.42)	14590.40(14541.72, 14639.20)	15184.39(15149.52, 15219.33)	14227.14(14179.05, 14275.34)	12445.16(12399.24, 12491.22)	13353.11(13319.83, 13386.47)	11687.27(11645.85, 11728.80)	10200.08(10160.54, 10239.74)	10959.28(10930.60,10988.01)
	China	NCD predominant	12493.53(12488.44, 12498.62)	10588.28(10583.48, 10593.08)	11567.68(11564.18, 11571.19)	9249.01(9244.22,9253.80)	7809.51(7804.91,7814.11)	8554.15(8550.83,8557.48)	7940.92(7935.96,7945.88)	7030.75(7025.76,7035.74)	7515.84(7512.31,7519.36)	7523.33(7518.46,7528.20)	6629.64(6624.70,6634.57)	7105.33(7101.86,7108.80)
	Democratic People's Republic of Korea	Injury excess	13204.49(13160.00, 13249.09)	10400.34(10363.94, 10436.85)	11669.28(11640.96, 11697.65)	12454.37(12415.65, 12493.18)	9715.11(9679.99,9750.33)	11119.48(11093.27, 11145.75)	11070.11(11031.56, 11108.77)	8676.83(8641.79,8711.98)	9898.03(9871.91,9924.19)	10001.61(9961.25,10042.11)	8130.08(8092.75,8167.55)	9085.55(9057.99,9113.16)

Super region	Country/territory	Class	ASDR(95%UI)											
			1990			2010			2021			2035		
			Male	Female	Both	Male	Female	Both	Male	Female	Both	Male	Female	Both
	Fiji	Multi-burden	16153.59(15925.71, 16383.97)	14488.39(14269.59, 14709.74)	15329.78(15171.58, 15489.24)	13498.24(13295.15, 13703.71)	12335.37(12135.96, 12537.29)	12933.50(12790.90, 13077.33)	14137.81(13926.82, 14351.22)	12996.43(12789.87, 13205.52)	13579.25(13431.35, 13728.39)	12318.66(12121.67, 12518.17)	11016.28(10825.54, 11209.70)	11681.03(11543.64,11819.72)
	Indonesia	Multi-burden	15929.21(15914.68, 15943.74)	16621.49(16606.81, 16636.17)	16286.92(16276.59, 16297.25)	12932.30(12920.01, 12944.59)	12266.52(12254.41, 12278.65)	12602.61(12593.98, 12611.24)	12151.36(12139.98, 12162.75)	10909.13(10898.01, 10920.26)	11548.61(11540.64, 11556.58)	10161.27(10150.53, 10172.02)	9208.55(9198.07,9219.03)	9695.49(9687.98,9703.01)
	Kiribati	Multi-burden	23917.88(23022.81, 24839.12)	19064.84(18262.18, 19894.14)	21495.70(20892.78, 22111.79)	20745.33(20065.35, 21442.69)	16367.93(15756.40, 16997.23)	18581.53(18122.55, 19049.27)	19095.50(18447.10, 19761.20)	15405.33(14828.27, 15999.28)	17229.53(16795.00, 17672.59)	16866.67(16309.93, 17437.84)	13733.95(13216.32, 14267.05)	15339.86(14957.97,15729.20)
	Lao People's Democratic Republic	Multi-burden	26754.83(26623.62, 26886.56)	24780.84(24659.14, 24903.02)	25746.39(25657.06, 25835.97)	16106.69(16030.52, 16183.13)	16154.25(16077.59, 16231.19)	16129.86(16075.81, 16184.05)	13701.41(13631.01, 13772.09)	13229.33(13159.53, 13299.42)	13466.47(13416.88, 13516.20)	11301.00(11240.54, 11361.70)	11060.28(10999.47, 11121.35)	11182.07(11139.18,11225.09)
	Malaysia	NCD predominant	14258.44(14213.15, 14303.83)	10931.31(10891.53, 10971.19)	12598.25(12568.11, 12628.46)	11523.09(11491.30, 11554.95)	9698.33(9668.20,9728.53)	10638.89(10616.94, 10660.88)	11694.00(11661.43, 11726.64)	9646.27(9615.72,9676.89)	10700.41(10678.03, 10722.83)	10484.25(10452.51, 10516.06)	8826.33(8796.21,8856.53)	9679.33(9657.41,9701.29)
	Maldives	NCD predominant	13118.33(12741.84, 13503.49)	16431.59(16010.90, 16860.75)	14796.38(14513.13, 15083.91)	8516.58(8289.18,8749.12)	9393.55(9137.90,9654.97)	8922.20(8751.82,9095.28)	8142.74(7905.51,8385.53)	9292.79(9012.41,9579.80)	8657.92(8476.42,8842.42)	7257.19(7046.56,7472.85)	8263.64(8032.07,8500.54)	7745.42(7588.95,7904.47)
	Marshall Islands	Multi-	24557.51(23378.32,	15121.06(14219.74,	19834.82(19090.13,	18450.38(17555.03,	16153.15(15293.49,	17337.99(16713.95,	16925.07(16053.25,	14948.31(14117.72,	15952.85(15347.82,	15071.20(14278.61,	14108.52(13320.06,	14601.67(14040.19

Super region	Country/territory	Class	ASDR(95%UI)											
			1990			2010			2021			2035		
			Male	Female	Both	Male	Female	Both	Male	Female	Both	Male	Female	Both
		burden	25782.46)	16066.92)	20602.06)	19379.81)	17049.03)	17979.55)	17832.31)	15815.49)	16575.82)	15897.19)	14932.64)	,15180.33)
	Mauritius	NCD predominant	10860.50(10701.73, 11021.07)	11943.69(11774.55, 12114.69)	11392.62(11276.62, 11509.54)	10825.60(10659.95, 10993.23)	9637.37(9479.56,9797.20)	10236.94(10122.36, 10352.53)	11984.97(11800.41, 12171.87)	10487.12(10311.81, 10664.83)	11241.79(11114.31, 11370.45)	11118.81(10911.01, 11329.71)	9363.70(9169.74,9560.86)	10248.46(10106.05,10392.45)
	Micronesia (Federated States of)	Injury excess	19663.06(18974.07, 20372.07)	15593.56(14984.37, 16222.34)	17622.35(17161.72, 18092.84)	16726.81(16119.64, 17351.69)	13717.13(13152.82, 14300.20)	15270.32(14853.50, 15696.22)	15776.62(15168.18, 16403.39)	12866.04(12301.76, 13449.75)	14363.09(13945.86, 14789.74)	14732.43(14091.48, 15395.98)	12310.14(11707.04, 12937.41)	13546.94(13104.74,14000.78)
	Myanmar	Multi-burden	25132.15(25093.32, 25171.02)	22132.54(22096.60, 22168.52)	23616.43(23589.99, 23642.88)	16714.21(16683.91, 16744.55)	15782.17(15753.16, 15811.22)	16240.09(16219.13, 16261.07)	16207.20(16178.66, 16235.78)	13152.69(13127.05, 13178.37)	14670.65(14651.48, 14689.84)	12921.19(12895.70, 12946.73)	10879.08(10855.27, 10902.93)	11914.62(11897.16,11932.10)
	Nauru	Multi-burden	21490.97(19193.87, 23991.43)	16031.47(14048.23, 18220.28)	18776.60(17244.58, 20410.19)	24526.38(22180.27, 27054.57)	17876.47(15838.27, 20104.99)	21273.27(19702.03, 22937.05)	20527.32(18446.03, 22782.16)	15567.26(13719.02, 17597.58)	18106.02(16698.37, 19601.97)	18737.36(16942.77, 20670.79)	14173.43(12554.17, 15944.79)	16546.06(15322.01,17842.28)
	Palau	Injury excess	15763.21(14238.42, 17410.91)	15819.11(14220.81, 17550.30)	15794.79(14682.93, 16970.13)	13207.19(11754.39, 14796.00)	20449.49(18548.79, 22497.32)	16649.71(15458.55, 17911.07)	12193.71(10679.89, 13866.98)	20874.61(18692.00, 23246.62)	16124.88(14826.49, 17506.98)	11479.27(9878.36,13281.82)	19687.69(17497.89, 22094.48)	15412.35(14059.54,16868.57)
	Papua New Guinea	Multi-burden	19599.22(19493.18, 19705.70)	19633.12(19523.07, 19743.63)	19621.81(19545.40, 19698.44)	16062.56(15992.03, 16133.33)	17376.13(17299.62, 17452.90)	16697.98(16646.06, 16750.03)	15958.27(15896.31, 16020.41)	16857.64(16790.67, 16924.82)	16386.11(16340.60, 16431.72)	13020.74(12974.21, 13067.39)	13974.21(13923.78, 14024.78)	13459.80(13425.62,13494.06)

Super region	Country/territory	Class	ASDR(95%UI)											
			1990			2010			2021			2035		
			Male	Female	Both	Male	Female	Both	Male	Female	Both	Male	Female	Both
	Philippines	Multi-burden	17839.99(17813.91, 17866.10)	13891.28(13868.30, 13914.28)	15861.49(15844.11, 15878.88)	13362.27(13343.63, 13380.93)	11604.45(11586.79, 11622.14)	12497.10(12484.25, 12509.97)	13312.12(13294.61, 13329.65)	11857.69(11840.75, 11874.64)	12600.14(12587.94, 12612.34)	11059.18(11043.61, 11074.77)	10019.24(10003.83, 10034.67)	10556.17(10545.21,10567.15)
	Samoa	Injury excess	15200.46(14767.73, 15643.05)	12240.97(11820.82, 12672.82)	13847.94(13543.80, 14157.42)	12502.45(12097.89, 12917.51)	11503.99(11100.15, 11919.23)	12024.28(11737.49, 12316.53)	12613.26(12221.96, 13014.18)	11617.09(11231.23, 12013.15)	12131.25(11855.56, 12411.88)	12311.56(11969.69, 12660.86)	11609.28(11266.33, 11960.13)	11969.14(11726.47,12215.63)
	Seychelles	NCD predominant	12988.21(12327.38, 13675.36)	10826.83(10225.64, 11454.42)	11895.93(11447.94, 12357.04)	10840.54(10260.43, 11446.46)	8943.45(8384.81,9530.42)	9959.09(9552.87,10378.76)	6971.83(6495.88,7473.80)	9907.00(9316.49,10525.21)	8380.41(8002.42,8771.75)	7014.91(6569.00,7483.83)	8419.04(7920.02,8942.08)	7703.01(7367.55,8050.18)
	Solomon Islands	Multi-burden	20519.18(20144.48, 20899.34)	16664.92(16323.42, 17011.98)	18615.23(18361.16, 18872.05)	17793.06(17507.47, 18082.24)	14837.12(14573.95, 15103.89)	16316.12(16121.80, 16512.24)	16751.40(16505.05, 17000.59)	14208.32(13975.96, 14443.62)	15505.20(15335.48, 15676.36)	15623.66(15413.85, 15835.65)	13342.46(13141.18, 13546.09)	14518.37(14372.60,14665.27)
	Sri Lanka	Injury excess	29413.77(29347.60, 29480.05)	16589.34(16539.54, 16639.26)	23016.11(22974.67, 23057.60)	24600.02(24538.20, 24661.97)	12339.36(12295.94, 12382.90)	18352.65(18315.12, 18390.25)	11266.67(11225.99, 11307.47)	10092.78(10054.29, 10131.37)	10674.87(10646.88, 10702.93)	10865.74(10823.05, 10908.56)	9176.77(9136.77,9216.91)	10024.38(9995.11, 10053.71)
	Thailand	Injury excess	19131.02(19102.73, 19159.35)	13035.18(13011.42, 13058.97)	16124.45(16105.94, 16142.99)	15622.61(15594.56, 15650.70)	10465.37(10442.08, 10488.69)	13065.76(13047.50, 13084.04)	15045.30(15014.49, 15076.16)	9965.91(9940.42,9991.44)	12512.67(12492.67, 12532.70)	12633.47(12601.26, 12665.74)	8778.50(8750.71,8806.37)	10753.72(10732.36,10775.11)
	Timor-Leste	Multi-	25955.05(25667.97,	20761.73(20492.55,	23480.02(23282.32,	13131.03(12962.78,	13254.51(13083.25,	13192.16(13072.03,	13006.40(12862.23,	12663.01(12518.60,	12836.16(12734.06,	11137.44(11009.96,	10266.54(10140.70,	10714.36(10624.65

Super region	Country/territory	Class	ASDR(95%UI)											
			1990			2010			2021			2035		
			Male	Female	Both	Male	Female	Both	Male	Female	Both	Male	Female	Both
		burden	26244.54)	21033.59)	23678.99)	13300.98)	13427.51)	13313.15)	13151.81)	12808.68)	12938.88)	11266.04)	10393.56)	,10804.65)
	Tonga	NCD predominant	11000.43(10501.33,11518.10)	10596.49(10091.32,11121.32)	10804.69(10448.37,11170.52)	11061.11(10548.30,11593.20)	10192.61(9689.71,10715.48)	10633.99(10273.48,11004.27)	10978.00(10461.89,11513.65)	9989.03(9488.01,10510.11)	10487.47(10126.51,10858.30)	9881.72(9435.19,10344.33)	9252.05(8801.95,9719.62)	9578.81(9260.58,9905.40)
	Tuvalu	Injury excess	21030.14(18539.34,23763.87)	17555.82(15295.06,20059.43)	19285.65(17588.76,21102.91)	16092.78(14182.17,18192.15)	13107.05(11280.28,15149.85)	14699.02(13357.30,16140.78)	14743.61(13046.70,16601.50)	12374.30(10729.63,14201.32)	13630.79(12433.47,14913.05)	13279.46(11737.71,14967.65)	11041.34(9570.41,12674.58)	12216.81(11137.20,13372.92)
	Vanuatu	Multi-burden	19484.12(18915.04,20066.45)	14455.57(13965.25,14959.01)	16971.12(16594.84,17353.97)	17189.47(16772.55,17614.34)	13509.44(13140.71,13885.96)	15331.65(15053.29,15613.94)	16600.55(16232.73,16974.72)	13056.76(12727.53,13392.38)	14831.28(14584.08,15081.65)	14513.57(14217.71,14814.05)	11607.31(11334.10,11885.47)	13108.90(12906.58,13313.61)
	Viet Nam	Injury excess	14416.99(14394.01,14440.00)	11572.70(11552.40,11593.03)	12970.91(12955.61,12986.22)	12134.02(12115.08,12152.99)	8876.98(8860.37,8893.62)	10532.48(10519.85,10545.12)	11202.79(11182.85,11222.76)	8680.88(8662.79,8698.99)	9979.08(9965.58,9992.60)	10443.61(10425.99,10461.25)	7965.41(7949.31,7981.54)	9255.11(9243.12,9267.11)
Sub-Saharan Africa	Angola	Multi-burden	36562.17(36467.38,36657.14)	27135.92(27053.78,27218.26)	31884.68(31821.89,31947.58)	19908.94(19859.56,19958.42)	20560.44(20512.35,20608.61)	20264.87(20230.39,20299.40)	18727.50(18689.00,18766.06)	18821.30(18783.57,18859.09)	18780.48(18753.53,18807.47)	14371.89(14345.95,14397.87)	14399.03(14373.04,14425.05)	14387.97(14369.60,14406.35)
	Benin	Multi-burden	21533.72(21421.72,21646.19)	21236.73(21130.85,21343.00)	21416.49(21339.57,21493.63)	18151.97(18081.93,18222.24)	18489.57(18420.70,18558.63)	18336.13(18287.00,18385.36)	16507.35(16452.20,16562.64)	16787.53(16733.29,16841.89)	16653.48(16614.81,16692.23)	13839.12(13799.17,13879.15)	13888.51(13847.93,13929.17)	13859.26(13830.80,13887.76)

Super region	Country/territory	Class	ASDR(95%UI)											
			1990			2010			2021			2035		
			Male	Female	Both	Male	Female	Both	Male	Female	Both	Male	Female	Both
	Botswana	Multi-burden	21696.18(21493.71, 21900.15)	20414.76(20228.00, 20602.86)	21033.36(20895.90, 21171.52)	22342.81(22178.62, 22507.92)	24420.57(24251.62, 24590.43)	23427.06(23309.00, 23545.57)	17522.02(17379.05, 17665.88)	18052.09(17906.60, 18198.48)	17792.21(17690.13, 17894.73)	12281.99(12167.14, 12397.67)	12153.51(12037.94, 12269.93)	12218.95(12137.43,12300.90)
	Burkina Faso	Multi-burden	24017.50(23934.17, 24101.06)	26460.93(26376.21, 26545.87)	25374.49(25314.81, 25434.28)	21106.44(21048.90, 21164.11)	20886.55(20831.94, 20941.28)	21004.86(20965.25, 21044.53)	19987.99(19939.91, 20036.15)	18779.96(18735.88, 18824.13)	19351.80(19319.31, 19384.33)	14388.69(14357.35, 14420.08)	14979.94(14947.79, 15012.15)	14670.97(14648.54,14693.43)
	Burundi	Multi-burden	29646.87(29526.94, 29767.18)	29486.39(29369.43, 29603.71)	29594.92(29511.09, 29678.94)	21758.55(21684.09, 21833.22)	21675.52(21603.64, 21747.59)	21715.96(21664.22, 21767.78)	17969.84(17911.29, 18028.54)	17875.10(17818.33, 17932.02)	17922.73(17881.96, 17963.58)	14206.77(14164.37, 14249.27)	13777.42(13735.81, 13819.13)	13989.21(13959.50,14018.96)
	Cabo Verde	Injury excess	13089.99(12790.06, 13395.34)	13432.36(13131.07, 13738.92)	13264.24(13051.29, 13479.86)	12111.53(11881.75, 12344.73)	11027.52(10804.35, 11254.20)	11579.71(11419.21, 11741.93)	12854.66(12601.97, 13111.22)	10220.02(9991.64,10452.36)	11555.98(11385.17, 11728.75)	11009.49(10762.19, 11261.30)	9003.86(8776.12,9236.29)	10017.50(9848.95, 10188.35)
	Cameroon	Multi-burden	20341.53(20269.58, 20413.69)	21988.88(21916.69, 22061.25)	21227.68(21176.63, 21278.83)	20217.07(20169.72, 20264.51)	22713.79(22664.45, 22763.21)	21521.57(21487.30, 21555.89)	18624.74(18586.97, 18662.57)	18712.73(18675.06, 18750.47)	18673.42(18646.74, 18700.13)	13364.93(13338.07, 13391.83)	13757.01(13729.23, 13784.84)	13554.15(13534.84,13573.48)
	Central African Republic	Multi-burden	37078.17(36891.11, 37265.97)	31957.04(31786.19, 32128.58)	34512.73(34386.10, 34639.72)	36535.79(36396.10, 36675.89)	30355.12(30230.30, 30480.34)	33394.14(33300.62, 33487.85)	36645.91(36518.22, 36773.95)	26186.47(26081.02, 26292.24)	31303.62(31221.15, 31386.25)	24269.36(24176.24, 24362.74)	18043.52(17962.93, 18124.38)	21165.68(21104.07,21227.42)
	Chad	Multi-	25204.57(25097.70,	23523.86(23424.17,	24341.85(24268.88,	21120.09(21050.75,	22876.28(22806.67,	22055.44(22006.22,	20638.60(20584.63,	21197.21(21143.55,	20930.84(20892.76,	15169.31(15135.44,	16470.77(16434.90,	15811.47(15786.82

Super region	Country/territory	Class	ASDR(95%UI)											
			1990			2010			2021			2035		
			Male	Female	Both	Male	Female	Both	Male	Female	Both	Male	Female	Both
		burden	25311.79)	23623.90)	24414.99)	21189.61)	22946.05)	22104.75)	20692.68)	21250.98)	20968.96)	15203.23)	16506.71)	,15836.16)
	Comoros	Multi-burden	20189.80(19867.72,20516.02)	20864.84(20537.80,21195.95)	20537.48(20307.61,20769.41)	14784.77(14545.89,15026.66)	16266.50(16016.24,16519.75)	15529.39(15356.16,15704.11)	14093.34(13872.90,14316.42)	15187.51(14957.22,15420.46)	14638.39(14478.84,14799.26)	11907.22(11710.15,12106.80)	12956.08(12746.45,13168.34)	12421.49(12277.60,12566.65)
	Congo	Multi-burden	27415.06(27251.03,27579.85)	25814.67(25656.89,25973.21)	26606.95(26493.14,26721.14)	20419.24(20305.34,20533.64)	23713.46(23594.38,23833.00)	22136.96(22054.32,22219.83)	18515.31(18421.21,18609.79)	19727.76(19631.72,19824.17)	19133.96(19066.67,19201.43)	14860.87(14784.45,14937.59)	15283.81(15205.62,15362.31)	15069.58(15014.91,15124.41)
	Côte d'Ivoire	Multi-burden	22670.59(22602.23,22739.11)	24078.07(24008.05,24148.24)	23417.46(23368.44,23466.56)	22284.29(22233.84,22334.83)	22372.59(22322.01,22423.25)	22347.65(22311.91,22383.44)	17148.72(17109.76,17187.75)	17420.98(17380.51,17461.52)	17283.57(17255.49,17311.68)	13175.55(13147.00,13204.16)	13467.40(13437.82,13497.04)	13315.51(13294.96,13336.08)
	Democratic Republic of the Congo	Multi-burden	25664.36(25623.54,25705.24)	25968.10(25926.65,26009.62)	25817.74(25788.64,25846.86)	23085.45(23056.62,23114.30)	23535.30(23505.66,23564.97)	23305.85(23285.18,23326.53)	20527.80(20504.62,20551.00)	19338.32(19315.46,19361.20)	19942.13(19925.85,19958.42)	15502.59(15485.28,15519.91)	14969.97(14952.71,14987.24)	15239.66(15227.44,15251.89)
	Djibouti	Multi-burden	22131.39(21799.58,22467.09)	18745.19(18423.11,19071.56)	20527.62(20295.47,20761.82)	16576.03(16370.07,16783.94)	17108.61(16883.25,17336.31)	16850.06(16697.76,17003.42)	15695.82(15521.25,15871.88)	15567.64(15373.47,15763.66)	15638.69(15508.76,15769.44)	12092.79(11952.65,12234.24)	11636.82(11490.90,11784.19)	11878.02(11776.86,11979.87)
	Equatorial Guinea	Multi-burden	31827.27(31379.62,32279.98)	26728.62(26334.76,27127.03)	29195.86(28898.72,29495.38)	16178.20(16001.18,16356.75)	21379.72(21153.92,21607.35)	18576.05(18434.82,18718.10)	18562.07(18412.28,18712.79)	20732.18(20544.70,20920.96)	19466.02(19348.75,19583.84)	14479.33(14352.62,14606.93)	15139.56(15000.60,15279.51)	14776.12(14682.50,14870.20)

Super region	Country/territory	Class	ASDR(95%UI)											
			1990			2010			2021			2035		
			Male	Female	Both	Male	Female	Both	Male	Female	Both	Male	Female	Both
	Eritrea	Multi-burden	119967.22(119674.74,120260.26)	40327.71(40157.02,40498.96)	80741.70(80571.29,80912.40)	23608.32(23509.16,23707.80)	18440.61(18349.29,18532.28)	21132.34(21064.62,21200.22)	20175.48(20090.48,20260.75)	16638.47(16558.38,16718.85)	18473.08(18414.50,18531.80)	16178.43(16108.82,16248.28)	13042.73(12978.55,13107.15)	14653.62(14606.13,14701.22)
	Eswatini	Multi-burden	21826.21(21565.14,22089.81)	17070.22(16853.97,17288.64)	19300.69(19132.91,19469.63)	34072.14(33800.64,34345.33)	38325.74(38052.54,38600.42)	36438.85(36245.21,36633.27)	30258.64(30007.20,30511.69)	30274.84(30021.68,30529.60)	30277.58(30099.06,30456.91)	19172.92(18980.14,19367.20)	17093.33(16907.06,17281.16)	18154.97(18020.71,18289.99)
	Ethiopia	Multi-burden	60441.63(60385.56,60497.74)	34615.16(34573.68,34656.68)	47337.46(47302.77,47372.16)	16902.00(16880.84,16923.18)	17460.85(17439.22,17482.51)	17190.11(17174.98,17205.26)	17908.00(17888.89,17927.12)	15354.75(15336.83,15372.69)	16647.71(16634.60,16660.82)	11927.32(11913.51,11941.14)	11587.82(11573.89,11601.76)	11761.27(11751.46,11771.08)
	Gabon	Multi-burden	23138.75(22893.11,23386.42)	19985.96(19763.67,20210.16)	21520.57(21355.18,21686.94)	19493.58(19308.07,19680.47)	19411.81(19235.48,19589.38)	19449.52(19321.64,19578.06)	18280.08(18116.56,18444.73)	16452.09(16306.38,16598.79)	17309.55(17200.55,17419.08)	14722.57(14591.82,14854.22)	12906.97(12784.87,13029.97)	13807.06(13717.62,13896.96)
	Gambia	Multi-burden	19275.84(19053.26,19500.45)	19700.99(19482.19,19921.67)	19507.85(19351.61,19665.07)	17108.67(16954.99,17263.43)	19045.89(18888.57,19204.21)	18114.81(18004.60,18225.53)	16586.70(16459.11,16715.05)	17668.65(17540.03,17798.01)	17144.75(17054.06,17235.82)	13251.36(13150.82,13352.50)	14236.95(14131.55,14342.95)	13739.07(13666.23,13812.20)
	Ghana	Multi-burden	19362.77(19305.55,19420.12)	21202.31(21143.02,21261.73)	20315.35(20274.09,20356.67)	18879.50(18836.71,18922.36)	19409.14(19366.46,19451.89)	19159.63(19129.39,19189.89)	16164.71(16130.07,16199.41)	15759.96(15725.95,15794.04)	15963.70(15939.43,15988.01)	13410.55(13383.09,13438.05)	12933.03(12905.53,12960.58)	13174.37(13154.94,13193.83)
	Guinea	Multi-	20220.89(201	24388.01(242	22433.29(223	19542.11(194	22122.20(220	20957.67(209	17438.94(173	19861.40(198	18713.53(186	13933.22(138	15713.70(156	14805.95(14

Super region	Country/territory	Class	ASDR(95%UI)											
			1990			2010			2021			2035		
			Male	Female	Both	Male	Female	Both	Male	Female	Both	Male	Female	Both
		burden	21.47, 20320.70)	82.34, 24494.04)	60.39, 22506.38)	70.04, 19614.41)	50.42, 22194.17)	06.62, 21008.81)	81.23, 17496.81)	02.00, 19920.93)	72.01, 18755.12)	92.49, 13974.04)	69.65, 15757.84)	775.99,14835.96)
	Guinea-Bissau	Multi-burden	30938.73(30658.89, 31220.61)	27092.01(26837.09, 27348.82)	28988.06(28798.98, 29178.12)	24236.46(24045.50, 24428.58)	23346.00(23163.55, 23529.55)	23798.96(23666.87, 23931.62)	21997.64(21836.02, 22160.18)	20926.85(20770.76, 21083.84)	21462.94(21350.56, 21575.79)	17106.15(16989.56, 17223.35)	15953.24(15839.06, 16068.05)	16537.56(16455.90,16619.54)
	Kenya	Multi-burden	16616.41(16574.96, 16657.95)	19641.70(19597.39, 19686.09)	18192.14(18161.70, 18222.63)	19358.94(19325.59, 19392.33)	18913.07(18880.44, 18945.75)	19154.47(19131.12, 19177.83)	16668.43(16640.79, 16696.11)	14867.17(14841.29, 14893.09)	15767.06(15748.13, 15786.01)	12031.81(12009.35, 12054.31)	10809.54(10787.97, 10831.15)	11428.61(11413.02,11444.21)
	Lesotho	Multi-burden	21451.86(21249.23, 21656.06)	17996.33(17826.52, 18167.42)	19594.25(19463.59, 19725.61)	34552.10(34338.27, 34766.95)	31708.18(31508.65, 31908.67)	33136.03(32989.79, 33282.76)	34764.85(34555.88, 34974.78)	30575.17(30379.74, 30771.56)	32679.93(32536.77, 32823.56)	22289.06(22120.77, 22458.35)	16465.43(16320.53, 16611.32)	19379.61(19268.49,19491.22)
	Liberia	Multi-burden	71198.55(70912.70, 71485.29)	33131.63(32944.77, 33319.30)	51418.32(51250.18, 51586.89)	18672.08(18564.96, 18779.69)	24145.72(24024.47, 24267.43)	21438.74(21357.70, 21520.02)	17433.18(17345.91, 17520.79)	21898.19(21800.30, 21996.41)	19672.12(19606.51, 19737.91)	14142.28(14073.12, 14211.70)	17618.10(17539.34, 17697.13)	15831.04(15778.79,15883.42)
	Madagascar	Multi-burden	22816.02(22746.95, 22885.25)	23459.37(23390.07, 23528.83)	23152.49(23103.54, 23201.53)	18229.78(18183.67, 18275.97)	20024.39(19976.79, 20072.08)	19146.15(19112.99, 19179.37)	17435.21(17397.15, 17473.33)	18946.67(18907.64, 18985.76)	18206.95(18179.67, 18234.26)	13951.99(13922.39, 13981.65)	15285.88(15254.55, 15317.25)	14612.51(14590.97,14634.08)
	Malawi	Multi-burden	25037.06(24957.58, 25116.75)	31494.43(31407.03, 31582.01)	28385.84(28326.53, 28445.26)	23418.69(23354.55, 23482.96)	25066.86(25003.09, 25130.75)	24308.37(24263.06, 24353.74)	20439.35(20390.99, 20487.79)	19901.01(19854.47, 19947.63)	20167.20(20133.66, 20200.78)	14341.74(14304.89, 14378.66)	13257.46(13222.02, 13292.97)	13796.59(13771.03,13822.19)

Super region	Country/territory	Class	ASDR(95%UI)											
			1990			2010			2021			2035		
			Male	Female	Both	Male	Female	Both	Male	Female	Both	Male	Female	Both
	Mali	Multi-burden	24664.37(24575.51, 24753.48)	26670.50(26580.53, 26760.70)	25751.15(25687.76, 25814.66)	18184.28(18130.23, 18238.46)	21465.34(21408.14, 21522.65)	19913.07(19873.56, 19952.64)	18354.87(18312.06, 18397.77)	20275.45(20230.73, 20320.26)	19333.15(19302.16, 19364.18)	13373.18(13344.75, 13401.66)	15540.40(15509.22, 15571.63)	14440.22(14419.15,14461.32)
	Mauritania	Multi-burden	18621.32(18468.49, 18775.14)	21508.96(21347.11, 21671.76)	20102.87(19991.31, 20214.92)	13095.84(12995.53, 13196.76)	16002.58(15894.30, 16111.43)	14592.35(14518.31, 14666.68)	12342.08(12258.55, 12426.07)	14030.63(13943.89, 14117.79)	13212.12(13151.79, 13272.68)	11077.68(11011.41, 11144.26)	12206.29(12135.65, 12277.25)	11633.08(11584.67,11681.64)
	Mozambique	Multi-burden	30489.34(30408.36, 30570.50)	25451.81(25383.55, 25520.22)	27776.86(27724.51, 27829.29)	26116.01(26060.34, 26171.77)	27805.64(27751.01, 27860.36)	27070.69(27031.59, 27109.83)	25519.73(25474.57, 25564.95)	28491.61(28446.27, 28537.01)	27129.02(27096.93, 27161.15)	16238.34(16209.37, 16267.35)	16972.26(16942.61, 17001.94)	16605.76(16585.04,16626.51)
	Namibia	Multi-burden	20514.13(20328.20, 20701.38)	17530.82(17362.39, 17700.51)	18993.43(18868.13, 19119.36)	21295.60(21142.35, 21449.70)	18910.03(18767.48, 19053.40)	20087.11(19982.49, 20192.14)	20859.00(20711.32, 21007.47)	17423.11(17289.47, 17557.52)	19124.42(19024.88, 19224.35)	14512.04(14396.25, 14628.55)	10917.67(10817.02, 11019.04)	12713.64(12636.89,12790.75)
	Niger	Multi-burden	23898.25(23808.21, 23988.57)	24328.19(24241.14, 24415.50)	24142.62(24079.97, 24205.39)	17373.32(17319.76, 17427.01)	19390.07(19334.87, 19445.41)	18421.47(18382.94, 18460.07)	17237.27(17196.26, 17278.36)	18390.55(18349.00, 18432.18)	17827.57(17798.36, 17856.82)	13180.93(13154.30, 13207.60)	15224.73(15195.60, 15253.92)	14182.24(14162.54,14201.97)
	Nigeria	Multi-burden	20600.13(20575.55, 20624.74)	21788.77(21764.93, 21812.63)	21229.63(21212.50, 21246.76)	17414.14(17397.91, 17430.38)	18935.61(18918.94, 18952.30)	18195.93(18184.28, 18207.58)	15447.24(15434.37, 15460.11)	16688.72(16676.03, 16701.41)	16105.89(16096.85, 16114.93)	12587.24(12577.67, 12596.81)	13505.59(13495.65, 13515.54)	13036.86(13029.96,13043.75)
	Rwanda	Multi-	39974.00(39853.48,	29933.52(29831.83,	34824.39(34745.85,	18500.50(18435.39,	18389.67(18326.09,	18445.54(18400.03,	15474.04(15421.04,	14845.86(14794.49,	15157.38(15120.48,	13987.98(13943.83,	12158.66(12117.08,	13071.47(13041.14

Super region	Country/territory	Class	ASDR(95%UI)											
			1990			2010			2021			2035		
			Male	Female	Both	Male	Female	Both	Male	Female	Both	Male	Female	Both
		burden	40094.81)	30035.49)	34903.07)	18565.80)	18453.42)	18491.13)	15527.17)	14897.37)	15194.36)	14032.24)	12200.34)	,13101.84)
	Sao Tome and Principe	Multi-burden	15214.32(14680.89,15763.42)	16743.09(16167.53,17334.84)	15981.82(15588.40,16383.19)	13417.08(12991.87,13852.91)	14187.84(13747.61,14638.80)	13800.24(13493.57,14112.24)	12329.69(11961.26,12706.86)	12105.22(11741.84,12477.28)	12216.43(11957.14,12480.06)	10244.89(9911.74,10586.64)	10206.69(9870.14,10552.12)	10223.82(9986.55,10465.45)
	Senegal	Multi-burden	20611.17(20527.29,20695.31)	21081.56(21000.10,21163.27)	20865.73(20807.25,20924.34)	15989.94(15934.19,16045.83)	17473.82(17415.90,17531.89)	16744.70(16704.48,16785.00)	14892.17(14845.77,14938.68)	15748.09(15698.75,15797.54)	15307.07(15273.25,15340.95)	11655.81(11619.28,11692.41)	12545.19(12506.30,12584.17)	12088.83(12062.19,12115.52)
	Sierra Leone	Multi-burden	23615.12(23490.22,23740.54)	21650.88(21535.88,21766.36)	22624.19(22539.45,22709.17)	20592.38(20502.51,20682.56)	23627.14(23533.56,23721.00)	22183.23(22118.16,22248.44)	17631.51(17562.36,17700.86)	20207.80(20135.42,20280.38)	18956.72(18906.57,19006.97)	13235.27(13183.26,13287.43)	15741.68(15684.63,15798.89)	14471.03(14432.47,14509.67)
	Somalia	Multi-burden	40464.75(40353.27,40576.48)	29558.72(29456.29,29661.44)	35423.02(35346.64,35499.54)	38340.00(38261.06,38419.05)	28736.37(28664.66,28808.23)	33783.62(33729.97,33837.35)	28602.72(28548.10,28657.42)	23627.37(23575.46,23679.36)	26224.51(26186.71,26262.35)	22248.55(22209.06,22288.09)	18122.53(18085.82,18159.30)	20250.81(20223.77,20277.87)
	South Africa	Multi-burden	18375.23(18340.04,18410.47)	17553.98(17520.48,17587.52)	17953.43(17929.16,17977.72)	22443.09(22409.88,22476.34)	26437.20(26401.22,26473.22)	24439.05(24414.57,24463.55)	21323.48(21289.96,21357.05)	18543.38(18511.62,18575.19)	19956.86(19933.74,19980.00)	16402.12(16373.40,16430.88)	12757.35(12731.66,12783.07)	14592.59(14573.30,14611.89)
	South Sudan	Multi-burden	24706.28(24610.01,24802.84)	23391.35(23293.12,23489.91)	24083.81(24015.00,24152.77)	22525.29(22449.87,22600.91)	20992.74(20917.78,21067.90)	21801.66(21748.43,21855.00)	25050.95(24973.60,25128.50)	22847.17(22773.13,22921.40)	23967.30(23913.77,24020.92)	19008.97(18952.65,19065.42)	17634.65(17578.53,17690.91)	18345.44(18305.65,18385.30)

Super region	Country/territory	Class	ASDR(95%UI)											
			1990			2010			2021			2035		
			Male	Female	Both	Male	Female	Both	Male	Female	Both	Male	Female	Both
	Togo	Multi-burden	20220.32(20103.85, 20337.33)	21972.31(21852.37, 22092.76)	21127.28(21043.56, 21211.27)	21050.41(20958.92, 21142.22)	21576.80(21485.65, 21668.26)	21350.63(21286.06, 21415.36)	17495.37(17423.74, 17567.24)	18169.92(18095.35, 18244.72)	17825.26(17773.59, 17877.06)	14096.53(14040.43, 14152.80)	14633.69(14575.57, 14691.98)	14358.69(14318.32,14399.15)
	Uganda	Multi-burden	28003.41(27939.39, 28067.55)	33221.75(33153.98, 33289.63)	30812.85(30765.95, 30859.81)	21762.21(21722.12, 21802.36)	21934.13(21895.01, 21973.30)	21884.20(21856.16, 21912.26)	18879.71(18848.19, 18911.28)	17676.81(17646.38, 17707.27)	18290.21(18268.30, 18312.15)	13334.83(13313.04, 13356.65)	12139.90(12118.68, 12161.15)	12748.18(12732.96,12763.42)
	United Republic of Tanzania	Multi-burden	23658.18(23609.94, 23706.51)	26949.21(26899.98, 26998.51)	25446.76(25412.15, 25481.42)	19265.83(19232.45, 19299.26)	21023.98(20990.47, 21057.53)	20204.07(20180.38, 20227.79)	16307.03(16280.75, 16333.34)	17402.74(17376.53, 17428.99)	16884.26(16865.68, 16902.85)	12174.98(12155.87, 12194.11)	12696.95(12677.33, 12716.60)	12434.83(12421.14,12448.54)
	Zambia	Multi-burden	26445.56(26356.97, 26534.39)	33441.60(33345.00, 33538.41)	30136.77(30070.83, 30202.83)	25275.43(25208.85, 25342.14)	27012.02(26945.14, 27079.03)	26212.12(26164.82, 26259.48)	21769.07(21717.51, 21820.73)	21336.43(21286.48, 21386.47)	21564.29(21528.39, 21600.25)	15287.11(15250.52, 15323.76)	14235.69(14200.28, 14271.16)	14765.21(14739.74,14790.71)
	Zimbabwe	Multi-burden	18339.33(18273.91, 18404.93)	24132.53(24059.07, 24206.17)	21349.64(21300.19, 21399.18)	29087.83(29014.43, 29161.37)	28593.51(28523.35, 28663.80)	28872.00(28821.22, 28922.85)	24945.59(24882.56, 25008.75)	22308.75(22250.62, 22367.00)	23600.52(23557.71, 23643.39)	17260.16(17213.47, 17306.95)	14608.89(14565.98, 14651.90)	15931.74(15900.03,15963.49)

Table S3 Beyond GDP indicators (HDI, GII, and ANS) in 193 countries and territories in 1990, 2010, 2021, and 2035

Super region	Country/territory	Beyond GDP Indicators											
		1990			2010			2021			2035		
		HDI	GII	ANS	HDI	GII	ANS	HDI	GII	ANS	HDI	GII	ANS
Central Europe, Eastern Europe, and Central Asia	Albania	0.65	0.22	-3.14	0.77	0.19	8.56	0.79	0.12	3.07	0.79	0.10	0.70
	Armenia	0.66	0.46	-10.75	0.74	0.35	0.15	0.77	0.20	0.91	0.81	0.13	0.93
	Azerbaijan	0.67	0.36	-8.24	0.73	0.34	22.40	0.74	0.33	9.28	0.77	0.33	8.03
	Belarus	0.71	0.29	-0.79	0.79	0.15	13.81	0.80	0.10	13.17	0.77	0.09	13.47
	Bosnia and Herzegovina	0.72	0.27	0.55	0.72	0.22	-3.44	0.78	0.13	5.71	0.80	0.14	-2.69
	Bulgaria	0.70	0.36	-0.74	0.79	0.23	6.50	0.80	0.21	8.53	0.85	0.19	8.31
	Croatia	0.63	0.32	13.22	0.82	0.14	3.61	0.87	0.09	8.27	0.94	0.06	2.50
	Czechia	0.75	0.28	1.20	0.87	0.14	3.71	0.89	0.12	9.68	0.92	0.07	7.35
	Estonia	0.74	0.39	5.96	0.86	0.16	8.41	0.89	0.10	16.51	0.92	0.05	21.27
	Georgia	0.65	0.43	12.71	0.76	0.43	-3.47	0.81	0.29	-5.60	0.82	0.28	-2.43
	Hungary	0.72	0.34	8.23	0.83	0.25	5.88	0.85	0.24	11.53	0.89	0.24	11.69
	Kazakhstan	0.67	0.53	0.44	0.77	0.27	7.12	0.80	0.18	-0.20	0.81	0.16	-0.64
	Kyrgyzstan	0.64	0.52	3.20	0.66	0.36	4.23	0.70	0.35	-2.65	0.76	0.38	4.98
	Latvia	0.73	0.40	-2.34	0.83	0.23	-0.02	0.87	0.15	3.07	0.88	0.08	4.30
	Lithuania	0.74	0.31	-4.67	0.85	0.15	7.69	0.88	0.11	12.10	0.88	0.06	12.59
	Mongolia	0.58	0.50	-4.55	0.70	0.41	3.07	0.73	0.30	-8.08	0.79	0.24	6.89
	Montenegro	0.75	0.22	-5.73	0.81	0.21	-8.25	0.84	0.12	7.80	0.85	0.10	3.39
	North Macedonia	0.61	0.43	3.69	0.75	0.17	2.95	0.76	0.14	14.35	0.80	0.08	11.58
	Poland	0.71	0.29	-21.52	0.85	0.17	5.63	0.88	0.11	10.83	0.93	0.07	36.32

Super region	Country/territory	Beyond GDP Indicators											
		1990			2010			2021			2035		
		HDI	GII	ANS	HDI	GII	ANS	HDI	GII	ANS	HDI	GII	ANS
	Republic of Moldova	0.69	0.48	13.62	0.72	0.24	9.01	0.77	0.16	6.71	0.80	0.12	8.83
	Romania	0.71	0.52	-10.83	0.81	0.34	4.98	0.83	0.24	4.95	0.85	0.20	5.12
	Russian Federation	0.74	0.46	-34.39	0.80	0.27	9.84	0.82	0.19	6.37	0.82	0.14	0.68
	Serbia	0.62	0.24	9.44	0.77	0.21	-0.02	0.80	0.12	5.66	0.87	0.11	1.89
	Slovakia	0.75	0.27	13.63	0.84	0.20	5.57	0.85	0.18	4.45	0.87	0.20	4.46
	Slovenia	0.74	0.29	6.03	0.89	0.13	6.09	0.92	0.07	11.22	0.95	0.05	9.71
	Tajikistan	0.62	0.60	13.66	0.63	0.37	-4.23	0.68	0.28	2.57	0.73	0.27	-8.17
	Turkmenistan	0.72	0.39	1.82	0.70	0.24	-9.84	0.74	0.20	-4.11	0.80	0.22	-7.42
	Ukraine	0.73	0.47	-16.94	0.77	0.30	-2.19	0.75	0.19	-2.03	0.70	0.15	-1.72
	Uzbekistan	0.61	0.44	15.27	0.67	0.27	11.48	0.72	0.24	2.72	0.79	0.22	2.64
High-income	Andorra	0.75	0.47	8.98	0.86	0.44	5.57	0.85	0.39	5.38	0.94	0.36	1.28
	Argentina	0.72	0.49	5.84	0.83	0.37	7.61	0.84	0.30	11.61	0.88	0.24	6.65
	Australia	0.86	0.19	9.23	0.92	0.14	7.47	0.95	0.07	6.42	0.97	0.03	4.96
	Austria	0.82	0.20	14.01	0.90	0.11	12.95	0.92	0.05	12.74	0.96	0.02	11.09
	Belgium	0.81	0.18	18.56	0.91	0.09	11.34	0.94	0.05	12.08	0.98	0.02	9.90
	Brunei Darussalam	0.78	0.39	4.87	0.82	0.32	32.90	0.82	0.28	23.99	0.82	0.25	23.11
	Canada	0.86	0.19	6.17	0.91	0.14	6.66	0.93	0.07	7.58	0.95	0.03	6.12
	Chile	0.71	0.51	4.78	0.81	0.35	11.31	0.86	0.20	-3.74	0.90	0.08	-0.12
	Cyprus	0.73	0.44	11.37	0.86	0.19	6.42	0.90	0.25	1.92	0.96	0.30	1.52
	Denmark	0.84	0.10	12.50	0.91	0.05	13.24	0.95	0.01	20.43	0.98	0.00	20.39

Super region	Country/territory	Beyond GDP Indicators											
		1990			2010			2021			2035		
		HDI	GII	ANS	HDI	GII	ANS	HDI	GII	ANS	HDI	GII	ANS
	Finland	0.81	0.11	11.80	0.91	0.07	10.22	0.94	0.03	11.22	0.95	0.01	12.35
	France	0.79	0.23	11.94	0.88	0.13	7.93	0.91	0.08	9.21	0.93	0.06	6.45
	Germany	0.83	0.18	11.77	0.93	0.10	11.49	0.95	0.07	14.52	0.97	0.04	12.97
	Greece	0.76	0.30	1.77	0.87	0.15	-7.52	0.89	0.12	-2.16	0.93	0.09	-1.48
	Iceland	0.83	0.19	4.01	0.93	0.09	-10.01	0.96	0.04	5.70	0.98	0.01	7.22
	Ireland	0.74	0.23	21.45	0.91	0.16	9.31	0.95	0.07	20.12	0.97	0.04	13.88
	Israel	0.78	0.34	11.37	0.89	0.14	13.68	0.91	0.08	19.20	0.96	0.05	15.60
	Italy	0.78	0.24	9.46	0.88	0.12	3.17	0.90	0.06	7.69	0.92	0.02	4.49
	Japan	0.85	0.17	16.16	0.90	0.12	3.82	0.92	0.08	3.55	0.93	0.04	2.56
	Luxembourg	0.79	0.21	18.62	0.91	0.11	13.70	0.93	0.04	20.36	0.95	0.02	19.49
	Malta	0.73	0.34	16.38	0.86	0.26	9.74	0.91	0.17	19.41	0.99	0.10	15.56
	Monaco	0.81	0.26	1.60	0.81	0.29	-4.25	0.78	0.30	-7.98	0.69	0.32	-12.38
	Netherlands	0.85	0.12	13.37	0.92	0.05	12.55	0.94	0.02	15.49	0.98	0.01	15.49
	New Zealand	0.81	0.24	12.96	0.92	0.17	8.18	0.94	0.09	9.86	0.96	0.07	9.60
	Norway	0.85	0.13	7.75	0.94	0.07	20.20	0.96	0.02	20.21	0.98	0.00	21.75
	Portugal	0.70	0.27	15.78	0.83	0.13	-2.00	0.87	0.08	3.06	0.92	0.04	6.27
	Republic of Korea	0.73	0.30	27.97	0.89	0.09	19.38	0.93	0.06	17.71	0.98	0.03	14.00
	San Marino	0.84	0.29	22.27	0.90	0.28	5.01	0.85	0.22	-3.66	0.87	0.20	-15.68
	Singapore	0.78	0.26	31.14	0.92	0.09	39.71	0.94	0.04	31.55	0.97	0.00	28.58
	Spain	0.76	0.19	12.58	0.87	0.10	7.43	0.90	0.06	7.98	0.93	0.05	5.56
	Sweden	0.81	0.10	15.37	0.91	0.05	17.40	0.95	0.02	19.75	0.98	0.01	19.25
	Switzerland	0.85	0.14	16.45	0.94	0.06	19.78	0.96	0.02	16.15	0.98	0.00	16.20
	United Kingdom	0.80	0.25	3.32	0.91	0.18	2.76	0.93	0.10	4.90	0.98	0.04	4.81

Super region	Country/territory	Beyond GDP Indicators											
		1990			2010			2021			2035		
		HDI	GII	ANS	HDI	GII	ANS	HDI	GII	ANS	HDI	GII	ANS
	United States of America	0.88	0.29	6.37	0.92	0.24	2.76	0.92	0.19	4.30	0.93	0.13	5.46
	Uruguay	0.70	0.43	14.46	0.78	0.32	17.90	0.81	0.24	13.91	0.87	0.16	10.88
Latin America and Caribbean	Antigua and Barbuda	0.69	0.46	26.47	0.81	0.35	2.77	0.82	0.30	3.80	0.84	0.33	4.16
	Bahamas	0.76	0.39	16.76	0.80	0.37	19.60	0.80	0.34	4.82	0.83	0.30	5.09
	Barbados	0.73	0.38	11.79	0.79	0.35	0.20	0.80	0.30	7.41	0.83	0.29	5.95
	Belize	0.61	0.51	59.73	0.72	0.41	9.28	0.70	0.45	11.99	0.69	0.37	12.56
	Bolivia (Plurinational State of)	0.55	0.60	-2.35	0.66	0.48	16.28	0.69	0.42	-2.04	0.62	0.38	0.12
	Brazil	0.62	0.58	7.40	0.72	0.45	3.35	0.76	0.40	-0.25	0.80	0.35	-0.37
	Colombia	0.61	0.52	6.24	0.73	0.45	5.03	0.75	0.42	1.60	0.81	0.36	5.08
	Costa Rica	0.66	0.41	17.63	0.77	0.32	16.13	0.80	0.24	16.92	0.86	0.17	15.91
	Cuba	0.68	0.41	17.17	0.78	0.33	17.04	0.74	0.30	15.32	0.75	0.27	11.95
	Dominica	0.58	0.61	11.80	0.73	0.52	-9.29	0.74	0.44	-2.97	0.73	0.39	-7.49
	Dominican Republic	0.58	0.57	10.33	0.71	0.49	15.07	0.76	0.43	21.58	0.79	0.36	16.71
	Ecuador	0.64	0.65	2.60	0.74	0.43	4.63	0.75	0.37	2.36	0.79	0.29	-4.15
	El Salvador	0.52	0.54	7.57	0.66	0.40	6.33	0.67	0.37	5.59	0.70	0.36	-1.32
	Grenada	0.74	0.41	5.96	0.78	0.29	-13.65	0.79	0.25	-2.80	0.82	0.19	-3.08
	Guatemala	0.49	0.58	1.18	0.61	0.54	-0.03	0.63	0.47	8.60	0.64	0.43	7.71
	Guyana	0.50	0.56	-7.41	0.65	0.46	0.37	0.72	0.42	-27.37	0.82	0.36	6.31
	Haiti	0.44	0.57	-2.26	0.45	0.61	18.80	0.55	0.62	7.98	0.56	0.60	7.22
	Honduras	0.51	0.64	6.57	0.60	0.47	13.30	0.62	0.41	19.36	0.68	0.41	19.53

Super region	Country/territory	Beyond GDP Indicators											
		1990			2010			2021			2035		
		HDI	GII	ANS	HDI	GII	ANS	HDI	GII	ANS	HDI	GII	ANS
	Jamaica	0.66	0.43	12.01	0.71	0.43	9.50	0.70	0.35	30.81	0.71	0.31	18.95
	Mexico	0.67	0.55	1.98	0.75	0.42	7.65	0.76	0.35	3.45	0.80	0.33	2.26
	Nicaragua	0.49	0.62	5.52	0.61	0.47	9.19	0.67	0.40	12.06	0.70	0.33	13.03
	Panama	0.67	0.54	12.22	0.78	0.48	20.01	0.81	0.40	19.42	0.85	0.37	21.82
	Paraguay	0.60	0.61	1.92	0.70	0.48	16.58	0.73	0.43	17.64	0.76	0.36	16.98
	Peru	0.62	0.57	0.80	0.72	0.40	14.10	0.76	0.36	6.47	0.82	0.34	9.09
	Saint Kitts and Nevis	0.65	0.54	24.02	0.79	0.50	21.16	0.83	0.37	17.05	0.87	0.30	10.37
	Saint Lucia	0.67	0.46	29.03	0.73	0.36	15.60	0.72	0.35	13.07	0.72	0.33	6.52
	Saint Vincent and the Grenadines	0.66	0.46	24.74	0.76	0.40	11.97	0.77	0.41	8.55	0.84	0.40	7.25
	Suriname	0.60	0.58	-0.70	0.70	0.51	24.11	0.69	0.41	0.48	0.70	0.34	-33.74
	Trinidad and Tobago	0.66	0.47	-18.94	0.78	0.34	-7.95	0.80	0.27	9.29	0.82	0.21	6.89
	Venezuela (Bolivarian Republic of)	0.66	0.57	0.87	0.76	0.49	17.57	0.69	0.52	8.82	0.69	0.62	1.03
North Africa and Middle East	Afghanistan	0.28	0.71	2.61	0.45	0.71	3.35	0.47	0.65	3.69	0.51	0.61	3.17
	Algeria	0.59	0.57	8.57	0.72	0.52	29.70	0.74	0.46	15.40	0.79	0.45	14.22
	Bahrain	0.73	0.37	-9.56	0.81	0.26	5.10	0.88	0.19	8.19	0.92	0.10	-3.76
	Egypt	0.57	0.65	9.87	0.67	0.55	6.13	0.73	0.39	-1.13	0.77	0.35	-11.99
	Iran (Islamic Republic of)	0.61	0.69	-3.37	0.76	0.55	22.83	0.78	0.49	8.15	0.82	0.45	13.93
	Iraq	0.50	0.70	17.87	0.63	0.60	16.03	0.67	0.56	9.44	0.67	0.53	5.92
	Jordan	0.62	0.57	16.41	0.73	0.49	21.81	0.74	0.46	4.17	0.74	0.38	2.75

Super region	Country/territory	Beyond GDP Indicators											
		1990			2010			2021			2035		
		HDI	GII	ANS	HDI	GII	ANS	HDI	GII	ANS	HDI	GII	ANS
	Kuwait	0.70	0.70	8.27	0.81	0.21	32.05	0.84	0.31	4.01	0.88	0.14	14.84
	Lebanon	0.67	0.50	11.71	0.75	0.43	-11.56	0.73	0.39	-27.91	0.67	0.31	-44.85
	Libya	0.72	0.34	-13.51	0.77	0.29	24.72	0.75	0.27	-30.07	0.74	0.31	-26.69
	Morocco	0.45	0.75	24.62	0.60	0.55	22.50	0.69	0.44	21.48	0.78	0.34	21.19
	Oman	0.74	0.32	-24.07	0.80	0.34	2.67	0.81	0.27	-10.32	0.82	0.23	-16.12
	Palestine	0.64	0.55	2.96	0.69	0.54	-1.44	0.71	0.44	9.70	0.73	0.26	0.68
	Qatar	0.76	0.27	4.14	0.83	0.67	32.47	0.86	0.22	25.47	0.92	0.13	38.09
	Saudi Arabia	0.70	0.56	-3.86	0.81	0.75	27.94	0.87	0.23	7.98	0.96	0.35	-0.31
	Sudan	0.32	0.75	-10.90	0.49	0.61	8.27	0.52	0.55	-8.50	0.55	0.45	-30.43
	Syrian Arab Republic	0.56	0.55	13.87	0.66	0.49	9.34	0.56	0.49	5.66	0.57	0.47	-5.73
	Tunisia	0.57	0.46	12.92	0.71	0.29	5.98	0.73	0.24	-1.36	0.73	0.20	-11.58
	Turkey	0.60	0.59	5.93	0.75	0.42	8.56	0.84	0.27	16.47	0.93	0.12	19.17
	United Arab Emirates	0.72	0.74	-3.00	0.83	0.18	4.84	0.93	0.04	4.26	0.99	0.01	6.49
	Yemen	0.36	0.63	7.40	0.50	0.79	8.06	0.42	0.82	6.73	0.37	0.89	-27.45
South Asia	Bangladesh	0.40	0.70	11.24	0.56	0.60	26.77	0.66	0.50	32.18	0.83	0.40	33.03
	Bhutan	0.41	0.72	27.40	0.58	0.48	24.88	0.68	0.32	13.35	0.73	0.20	15.35
	India	0.43	0.69	7.11	0.57	0.56	22.89	0.63	0.45	15.41	0.74	0.28	17.37
	Nepal	0.40	0.74	3.50	0.54	0.53	32.45	0.59	0.50	24.42	0.70	0.41	21.92
	Pakistan	0.39	0.81	3.48	0.50	0.59	5.59	0.54	0.52	6.13	0.59	0.41	10.94

Super region	Country/territory	Beyond GDP Indicators											
		1990			2010			2021			2035		
		HDI	GII	ANS	HDI	GII	ANS	HDI	GII	ANS	HDI	GII	ANS
Southeast Asia, East Asia, and Oceania	Cambodia	0.38	0.66	16.44	0.54	0.51	10.73	0.60	0.48	17.34	0.67	0.45	23.14
	China	0.48	0.35	14.54	0.70	0.25	25.81	0.79	0.19	16.33	0.84	0.16	12.01
	Democratic People's Republic of Korea	0.64	0.38	32.00	0.70	0.23	29.39	0.73	0.22	27.95	0.76	0.24	26.12
	Fiji	0.63	0.49	-3.38	0.70	0.41	8.09	0.72	0.33	8.39	0.74	0.27	8.77
	Indonesia	0.53	0.61	6.08	0.67	0.50	10.89	0.71	0.44	10.78	0.77	0.39	13.11
	Kiribati	0.53	0.82	45.85	0.58	0.52	8.80	0.63	0.57	32.14	0.65	0.54	31.71
	Lao People's Democratic Republic	0.41	0.65	0.34	0.56	0.53	-17.01	0.62	0.47	-5.56	0.67	0.40	-24.84
	Malaysia	0.65	0.38	-5.15	0.77	0.26	11.43	0.80	0.20	0.36	0.88	0.13	1.04
	Maldives	0.60	0.67	19.03	0.69	0.40	17.63	0.75	0.33	23.02	0.79	0.22	15.89
	Marshall Islands	0.67	0.39	20.48	0.65	0.66	15.82	0.73	0.62	7.09	0.75	0.58	4.46
	Mauritius	0.62	0.53	23.54	0.75	0.37	2.90	0.79	0.37	0.00	0.83	0.39	0.61
	Micronesia (Federated States of)	0.65	0.80	28.14	0.64	0.76	37.50	0.63	0.45	35.05	0.62	0.75	32.84
	Myanmar	0.33	0.49	-21.54	0.51	0.56	12.07	0.60	0.48	17.85	0.70	0.42	6.80
	Nauru	0.62	0.46	1.58	0.56	0.87	-0.73	0.69	0.66	-3.13	0.76	0.77	-8.14
	Palau	0.58	0.61	5.89	0.77	0.46	4.39	0.80	0.45	5.63	0.74	0.54	9.09
	Papua New Guinea	0.39	0.69	-4.76	0.50	0.68	7.34	0.56	0.81	1.20	0.62	0.58	9.98
	Philippines	0.60	0.53	14.51	0.67	0.44	24.76	0.69	0.39	7.68	0.75	0.34	-33.50
	Samoa	0.71	0.53	2.20	0.70	0.47	20.57	0.71	0.44	22.16	0.73	0.41	23.00
	Seychelles	0.74	0.35	20.67	0.77	0.24	6.07	0.79	0.25	-6.12	0.80	0.37	-8.90

Super region	Country/territory	Beyond GDP Indicators											
		1990			2010			2021			2035		
		HDI	GII	ANS	HDI	GII	ANS	HDI	GII	ANS	HDI	GII	ANS
	Solomon Islands	0.57	0.47	-41.53	0.55	0.80	12.04	0.56	0.49	15.24	0.60	0.56	17.37
	Sri Lanka	0.64	0.45	20.46	0.74	0.41	19.71	0.78	0.38	25.38	0.78	0.35	23.92
	Thailand	0.58	0.46	21.80	0.74	0.37	14.65	0.80	0.31	9.22	0.90	0.28	9.77
	Timor-Leste	0.51	0.60	11.11	0.64	0.53	47.96	0.57	0.42	-81.62	0.65	0.29	-198.31
	Tonga	0.64	0.78	21.31	0.71	0.76	3.92	0.74	0.76	-3.35	0.77	0.45	8.56
	Tuvalu	0.56	0.43	2.86	0.62	0.83	-6.62	0.65	0.58	-16.73	0.66	0.63	-28.85
	Vanuatu	0.60	0.47	19.30	0.58	0.52	10.66	0.61	0.80	35.01	0.63	0.55	35.36
	Viet Nam	0.49	0.43	6.39	0.68	0.36	17.32	0.72	0.38	19.04	0.77	0.38	19.54
Sub-Saharan Africa	Angola	0.45	0.68	7.18	0.52	0.55	1.76	0.59	0.53	13.40	0.65	0.47	7.75
	Benin	0.35	0.70	-9.23	0.48	0.64	-0.88	0.50	0.64	10.55	0.51	0.65	11.50
	Botswana	0.59	0.59	31.54	0.65	0.50	19.47	0.68	0.49	8.50	0.73	0.45	10.37
	Burkina Faso	0.40	0.63	11.16	0.37	0.60	4.72	0.45	0.63	-8.33	0.43	0.63	-5.13
	Burundi	0.28	0.63	-19.59	0.40	0.53	-30.95	0.42	0.50	-16.74	0.47	0.48	-26.09
	Cabo Verde	0.52	0.58	2.08	0.65	0.41	26.66	0.65	0.33	23.75	0.69	0.27	31.45
	Cameroon	0.44	0.69	-2.99	0.52	0.62	-3.97	0.58	0.56	-1.61	0.70	0.51	-1.05
	Central African Republic	0.33	0.75	-0.03	0.36	0.70	8.92	0.39	0.67	-0.24	0.40	0.68	-2.22
	Chad	0.30	0.76	-14.13	0.36	0.75	-11.58	0.39	0.66	-11.86	0.42	0.61	-5.89
	Comoros	0.51	0.87	2.66	0.52	0.65	5.67	0.58	0.54	5.41	0.61	0.52	3.19

Super region	Country/territory	Beyond GDP Indicators											
		1990			2010			2021			2035		
		HDI	GII	ANS	HDI	GII	ANS	HDI	GII	ANS	HDI	GII	ANS
	Congo	0.54	0.65	-30.94	0.58	0.62	-3.55	0.60	0.58	-30.22	0.61	0.47	-31.92
	Côte d'Ivoire	0.42	0.68	6.63	0.47	0.64	-21.52	0.53	0.61	15.18	0.59	0.59	19.36
	Democratic Republic of the Congo	0.38	0.67	-4.79	0.42	0.66	-9.20	0.48	0.61	-11.19	0.52	0.56	-22.95
	Djibouti	0.48	0.50	21.02	0.42	0.49	9.39	0.51	0.47	9.65	0.60	0.62	2.89
	Equatorial Guinea	0.52	0.69	-4.67	0.61	0.60	-33.74	0.65	0.55	6.44	0.64	0.50	37.35
	Eritrea	0.45	0.61	20.07	0.46	0.54	7.02	0.49	0.51	1.60	0.48	0.45	2.04
	Eswatini	0.55	0.59	8.69	0.50	0.57	-6.03	0.61	0.50	4.67	0.58	0.47	3.39
	Ethiopia	0.31	0.68	2.59	0.41	0.60	6.76	0.49	0.50	13.44	0.56	0.42	11.11
	Gabon	0.60	0.59	-24.11	0.66	0.52	-0.09	0.69	0.52	-13.17	0.69	0.50	3.34
	Gambia	0.32	0.77	6.98	0.45	0.66	-19.91	0.49	0.59	17.84	0.50	0.52	18.58
	Ghana	0.44	0.65	-4.51	0.57	0.57	-9.70	0.60	0.51	3.78	0.68	0.47	1.12
	Guinea	0.27	0.74	5.47	0.42	0.68	-9.66	0.47	0.64	-16.28	0.49	0.57	-24.89
	Guinea-Bissau	0.36	0.69	-12.52	0.44	0.67	-26.23	0.48	0.63	-1.85	0.52	0.59	-6.90
	Kenya	0.48	0.71	5.20	0.54	0.60	-5.05	0.60	0.54	7.15	0.60	0.46	7.71
	Lesotho	0.48	0.64	-4.40	0.47	0.59	11.26	0.52	0.56	5.00	0.51	0.55	2.60
	Liberia	0.35	0.68	1.58	0.46	0.66	-12.38	0.48	0.66	-15.39	0.49	0.63	-10.07
	Madagascar	0.49	0.67	24.56	0.49	0.61	-0.37	0.48	0.58	-3.26	0.47	0.52	8.63
	Malawi	0.30	0.72	-7.13	0.46	0.61	6.06	0.51	0.58	24.84	0.55	0.55	31.27
	Mali	0.24	0.68	3.43	0.41	0.67	1.07	0.41	0.61	2.17	0.45	0.61	-1.09

Super region	Country/territory	Beyond GDP Indicators											
		1990			2010			2021			2035		
		HDI	GII	ANS	HDI	GII	ANS	HDI	GII	ANS	HDI	GII	ANS
	Mauritania	0.40	0.79	-2.13	0.51	0.64	16.46	0.54	0.60	24.26	0.56	0.53	22.63
	Mozambique	0.24	0.67	11.06	0.41	0.54	1.67	0.46	0.48	-6.98	0.55	0.41	-9.44
	Namibia	0.59	0.55	24.75	0.58	0.54	12.51	0.62	0.45	-1.83	0.48	0.42	1.07
	Niger	0.21	0.81	-7.09	0.34	0.69	21.52	0.39	0.61	34.37	0.46	0.63	37.10
	Nigeria	0.35	0.65	33.27	0.49	0.67	6.36	0.54	0.68	18.46	0.60	0.65	15.45
	Rwanda	0.32	0.62	11.61	0.49	0.45	-2.64	0.54	0.40	-1.50	0.63	0.32	-10.01
	Sao Tome and Principe	0.48	0.57	-3.43	0.55	0.57	12.07	0.61	0.58	-5.22	0.67	0.72	-8.23
	Senegal	0.37	0.68	-0.44	0.47	0.58	8.17	0.51	0.51	11.08	0.56	0.46	20.14
	Sierra Leone	0.31	0.66	-23.51	0.42	0.65	5.96	0.46	0.61	11.98	0.49	0.57	-7.38
	Somalia	0.39	0.65	-14.51	0.38	0.69	-17.10	0.38	0.66	-10.18	0.35	0.76	-13.74
	South Africa	0.63	0.50	-3.57	0.68	0.46	2.11	0.72	0.40	-1.30	0.75	0.40	-2.31
	South Sudan	0.44	0.60	11.56	0.41	0.63	-39.77	0.38	0.62	6.57	0.32	0.61	13.77
	Togo	0.40	0.75	10.49	0.47	0.63	-0.10	0.55	0.58	9.27	0.62	0.51	8.12
	Uganda	0.33	0.66	-18.26	0.50	0.55	-0.14	0.55	0.53	-4.41	0.64	0.47	3.70
	United Republic of Tanzania	0.37	0.67	-17.51	0.49	0.56	8.69	0.53	0.51	23.16	0.56	0.46	35.96
	Zambia	0.42	0.69	-5.14	0.53	0.58	6.20	0.57	0.53	6.06	0.61	0.47	3.29
	Zimbabwe	0.48	0.59	0.04	0.48	0.59	-19.38	0.55	0.52	0.35	0.51	0.53	-3.19

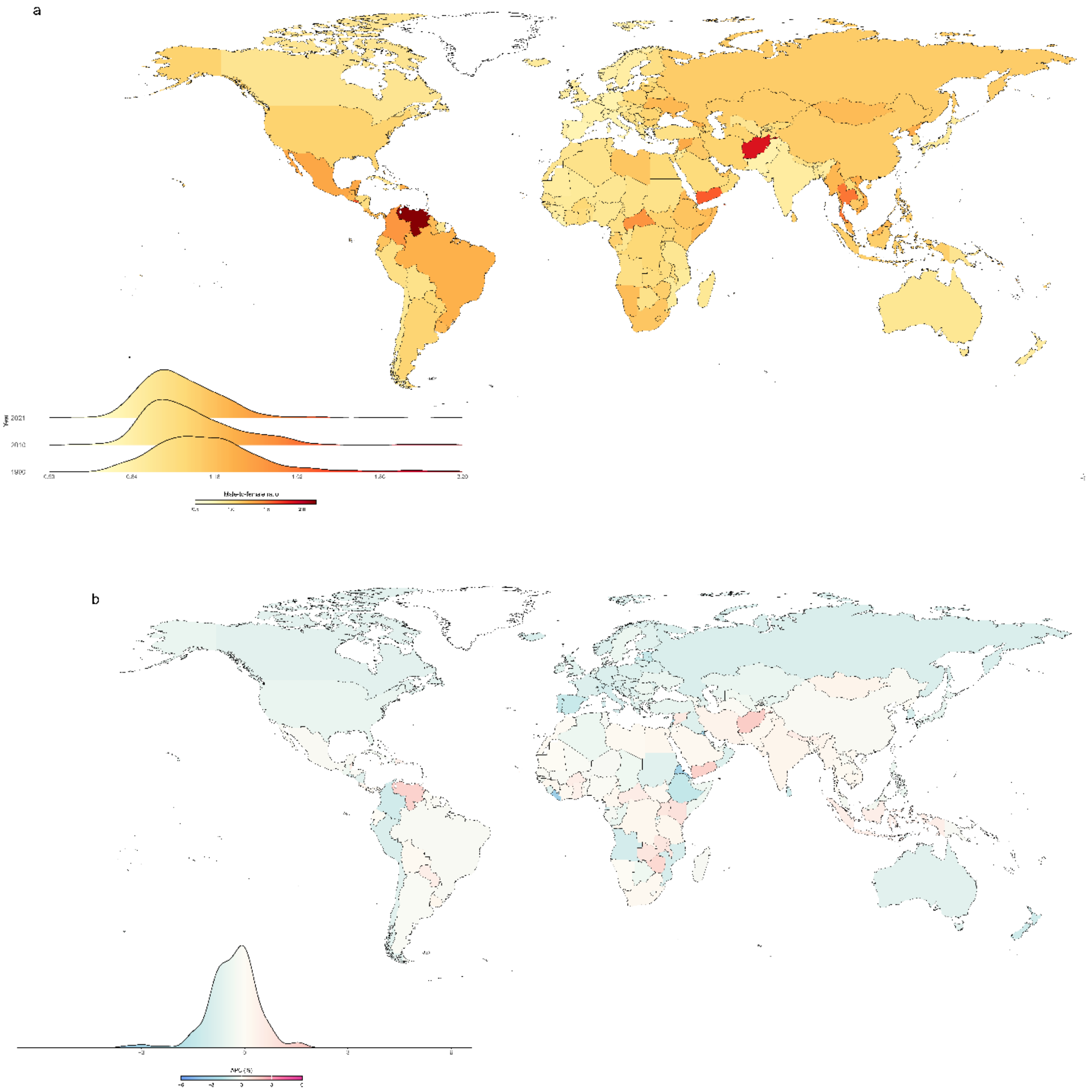

Figure S1 Regional patterns of sex disparities in all-cause ASDR in 193 countries (2010–2021)

(a) Regional distribution of male-to-female ratio of all-cause ASDR in 2021 and (b) APC of male-to-female ratio from 2010 to 2021.

Table S4 Association of all-cause ASDR with HDI, GII, and ANS in different country groups in 1990, 2010, 2021, 2035

Class	RR(95%CI)											
	1990			2010			2021			2035		
	HDI	GII	ANS	HDI	GII	ANS	HDI	GII	ANS	HDI	GII	ANS
All	0.78(0.73,0.82)	1.06(1.00,1.13)	0.97(0.94,1.01)	0.79(0.74,0.84)	1.09(1.02,1.15)	0.97(0.93,1.01)	0.84(0.79,0.89)	1.11(1.06,1.17)	0.97(0.95,1.00)	0.92(0.88,0.96)	1.15(1.11,1.20)	1.00(0.98,1.01)
Injury excess	0.85(0.74,0.99)	1.10(0.97,1.25)	0.97(0.93,1.03)	0.99(0.85,1.14)	0.93(0.87,1.01)	0.98(0.92,1.04)	0.99(0.89,1.10)	0.87(0.80,0.96)	0.96(0.92,1.00)	0.94(0.84,1.04)	0.87(0.80,0.94)	0.94(0.90,0.99)
Multi-burden	0.80(0.71,0.90)	0.94(0.80,1.11)	1.01(0.95,1.07)	0.79(0.67,0.93)	0.98(0.80,1.20)	0.96(0.89,1.04)	0.84(0.76,0.94)	0.89(0.79,0.99)	0.97(0.93,1.01)	0.91(0.86,0.96)	0.90(0.85,0.96)	1.00(0.98,1.02)
NCD predominant	1.03(0.95,1.12)	1.13(1.07,1.19)	0.97(0.93,1.01)	0.99(0.93,1.06)	0.93(0.89,0.97)	0.97(0.94,1.00)	0.99(0.93,1.06)	0.95(0.90,1.00)	0.96(0.92,0.99)	1.07(1.00,1.14)	0.92(0.87,0.97)	0.96(0.94,0.99)
Global North	0.97 (0.87,1.07)	1.08 (1.00,1.17)	1.00 (0.95,1.05)	1.01 (0.92,1.11)	1.15 (1.07,1.22)	1.05 (0.98,1.12)	1.07 (0.98,1.18)	1.19 (1.10,1.28)	0.99 (0.93,1.06)	1.06 (0.97,1.15)	1.16 (1.08,1.24)	1.01 (0.95,1.07)
Global South	0.78 (0.72,0.83)	1.07 (0.98,1.17)	0.96 (0.92,1.00)	0.78 (0.72,0.85)	1.10 (1.01,1.20)	0.96 (0.92,1.01)	0.82 (0.76,0.87)	1.12 (1.05,1.20)	0.96 (0.93,0.99)	0.91 (0.87,0.96)	1.14 (1.09,1.20)	0.99 (0.98,1.01)

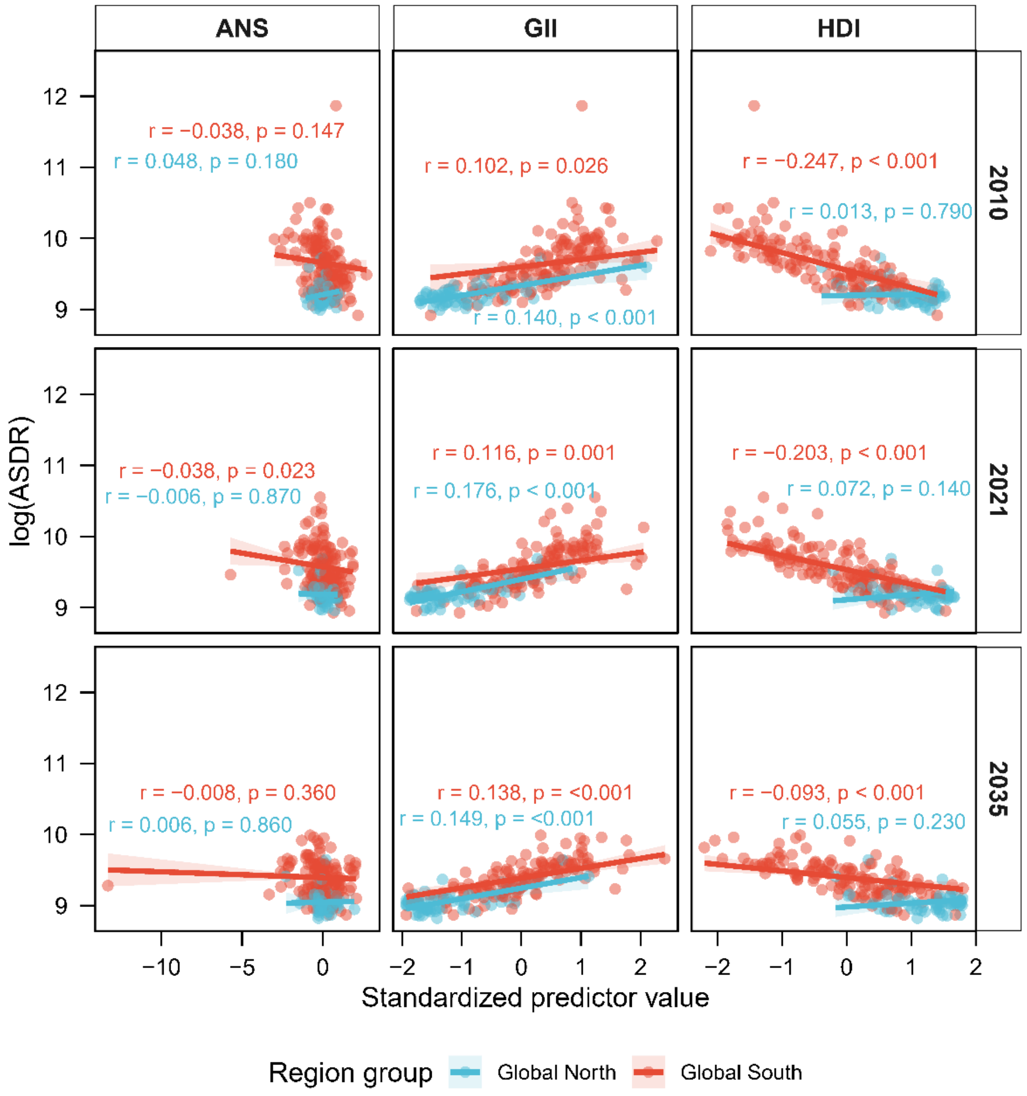

Figure S2 Association of all-cause ASDR with Human Development Index (HDI), Gender Inequality Index (GII), and Adjusted Net Savings (ANS) in Global North and South Countries in 2010, 2021,2035

Table S5 Association of ASDR Caused by Level 2 Causes with HDI, GII, and ANS in 2010, 2021, 2035

Cause	RR(95%CI)											
	1990			2010			2021			2035		
	HDI	GII	ANS	HDI	GII	ANS	HDI	GII	ANS	HDI	GII	ANS
**Communicable, maternal, neonatal, and nutritional diseases**	0.45(0.42,0.49)	1.30(1.21,1.41)	0.95(0.91,1.00)	0.40(0.36,0.45)	1.17(1.05,1.29)	0.95(0.89,1.01)	0.47(0.42,0.53)	1.18(1.06,1.32)	0.96(0.90,1.02)	0.60(0.54,0.66)	1.35(1.22,1.48)	1.03(0.98,1.08)
HIV/AIDS and sexually transmitted infections	0.47(0.33,0.69)	1.09(0.76,1.58)	0.91(0.73,1.13)	0.26(0.18,0.38)	1.00(0.70,1.44)	0.79(0.64,0.97)	0.35(0.24,0.52)	1.32(0.90,1.95)	0.90(0.75,1.09)	0.59(0.43,0.82)	1.74(1.27,2.40)	1.03(0.86,1.22)
Respiratory infections and tuberculosis	0.55(0.48,0.64)	1.34(1.17,1.55)	1.00(0.92,1.08)	0.49(0.43,0.56)	1.19(1.05,1.34)	0.99(0.92,1.06)	0.59(0.52,0.67)	1.11(0.98,1.25)	0.92(0.86,0.98)	0.68(0.60,0.76)	1.37(1.22,1.53)	0.99(0.93,1.05)
Enteric infections	0.39(0.30,0.50)	1.76(1.38,2.26)	1.10(0.95,1.28)	0.36(0.28,0.47)	1.43(1.12,1.83)	1.06(0.92,1.22)	0.46(0.35,0.60)	1.79(1.36,2.36)	1.08(0.95,1.24)	0.49(0.37,0.64)	1.75(1.33,2.29)	1.06(0.92,1.22)
Neglected tropical diseases and malaria	0.24(0.19,0.30)	1.80(1.42,2.28)	0.89(0.78,1.03)	0.21(0.17,0.26)	1.52(1.22,1.91)	1.03(0.90,1.17)	0.28(0.22,0.36)	1.78(1.41,2.24)	0.95(0.85,1.06)	0.40(0.31,0.52)	2.23(1.74,2.85)	1.06(0.93,1.21)
Other infectious diseases	0.41(0.36,0.47)	1.34(1.18,1.52)	0.94(0.87,1.02)	0.38(0.34,0.43)	1.18(1.05,1.32)	0.96(0.90,1.02)	0.43(0.37,0.49)	1.20(1.05,1.37)	1.01(0.95,1.08)	0.51(0.45,0.58)	1.38(1.20,1.57)	1.05(0.98,1.13)

Cause	RR(95%CI)											
	1990			2010			2021			2035		
	HDI	GII	ANS	HDI	GII	ANS	HDI	GII	ANS	HDI	GII	ANS
Maternal and neonatal disorders	0.56(0.51,0.61)	1.15(1.05,1.26)	0.97(0.92,1.03)	0.59(0.54,0.63)	1.08(1.00,1.17)	0.97(0.93,1.01)	0.64(0.59,0.69)	1.09(1.01,1.18)	1.01(0.97,1.05)	0.73(0.67,0.78)	1.14(1.06,1.23)	1.03(0.99,1.07)
Nutritional deficiencies	0.62(0.54,0.72)	1.48(1.29,1.70)	0.94(0.86,1.02)	0.52(0.46,0.60)	1.24(1.08,1.42)	0.99(0.92,1.07)	0.55(0.48,0.64)	1.25(1.07,1.45)	0.98(0.91,1.06)	0.62(0.53,0.72)	1.32(1.14,1.53)	1.02(0.95,1.11)
**Non-communicable diseases**	0.99(0.97,1.00)	1.02(1.00,1.04)	1.00(0.99,1.01)	1.02(1.00,1.05)	1.06(1.03,1.08)	0.97(0.96,0.99)	1.04(1.00,1.07)	1.06(1.03,1.09)	0.98(0.97,1.00)	1.03(1.00,1.06)	1.06(1.03,1.10)	0.99(0.97,1.00)
Neoplasms	1.07(0.99,1.15)	1.01(0.94,1.09)	0.99(0.94,1.03)	1.01(0.93,1.10)	1.06(0.97,1.14)	0.99(0.95,1.04)	0.95(0.87,1.04)	1.06(0.96,1.16)	0.97(0.93,1.02)	0.96(0.87,1.05)	1.10(1.01,1.21)	0.97(0.92,1.02)
Cardiovascular diseases	0.90(0.81,1.00)	1.31(1.19,1.45)	0.99(0.93,1.05)	0.84(0.75,0.94)	1.46(1.31,1.62)	1.04(0.98,1.11)	0.88(0.78,1.01)	1.59(1.40,1.81)	0.95(0.90,1.02)	0.90(0.79,1.02)	1.61(1.43,1.82)	0.94(0.88,1.01)
Chronic respiratory diseases	0.93(0.85,1.02)	1.01(0.93,1.11)	1.05(1.00,1.11)	1.04(0.95,1.15)	1.12(1.02,1.23)	0.95(0.90,1.00)	1.07(0.96,1.19)	1.17(1.05,1.30)	1.00(0.95,1.05)	1.02(0.93,1.12)	1.21(1.10,1.33)	1.00(0.95,1.05)
Digestive diseases	0.72(0.66,0.79)	1.28(1.17,1.40)	0.99(0.94,1.04)	0.64(0.58,0.69)	1.12(1.03,1.22)	0.98(0.93,1.03)	0.65(0.59,0.72)	1.14(1.04,1.26)	1.00(0.96,1.05)	0.74(0.67,0.81)	1.26(1.15,1.38)	1.03(0.98,1.08)
Neurological disorders	1.02(0.99,1.06)	1.05(1.01,1.08)	1.00(0.98,1.02)	1.02(0.98,1.05)	1.04(1.01,1.08)	0.97(0.95,0.99)	1.02(0.98,1.06)	1.05(1.01,1.09)	0.99(0.97,1.01)	1.04(1.00,1.07)	1.07(1.04,1.11)	0.99(0.97,1.01)
Mental disorders	1.08(1.03,1.13)	0.99(0.94,1.03)	1.00(0.97,1.03)	1.15(1.09,1.21)	1.04(0.99,1.10)	0.97(0.95,1.00)	1.13(1.07,1.20)	1.01(0.95,1.07)	0.97(0.94,0.99)	1.07(1.01,1.13)	0.99(0.93,1.04)	0.98(0.95,1.01)
Substance use disorders	1.37(1.23,1.52)	0.86(0.77,0.95)	1.01(0.95,1.07)	1.39(1.25,1.54)	0.83(0.75,0.92)	0.97(0.91,1.03)	1.38(1.22,1.55)	0.85(0.75,0.95)	0.98(0.93,1.04)	1.30(1.16,1.46)	0.83(0.74,0.93)	0.99(0.93,1.05)

Cause	RR(95%CI)											
	1990			2010			2021			2035		
	HDI	GII	ANS	HDI	GII	ANS	HDI	GII	ANS	HDI	GII	ANS
Diabetes and kidney diseases	0.76(0.69,0.85)	1.31(1.18,1.46)	0.97(0.91,1.03)	0.79(0.70,0.89)	1.42(1.27,1.60)	0.98(0.92,1.05)	0.87(0.77,0.99)	1.55(1.36,1.76)	0.97(0.92,1.04)	0.97(0.84,1.11)	1.80(1.57,2.06)	0.99(0.92,1.06)
Skin and subcutaneous diseases	1.00(0.96,1.05)	0.97(0.93,1.01)	1.04(1.02,1.07)	1.05(1.00,1.10)	1.02(0.98,1.07)	1.01(0.99,1.04)	1.09(1.03,1.14)	1.07(1.01,1.12)	1.01(0.99,1.04)	1.08(1.03,1.13)	1.07(1.02,1.12)	1.00(0.98,1.03)
Sense organ diseases	0.96(0.92,1.00)	1.12(1.08,1.17)	0.98(0.96,1.00)	0.94(0.89,0.98)	1.10(1.05,1.14)	1.03(1.00,1.05)	0.94(0.89,0.99)	1.08(1.03,1.13)	0.98(0.96,1.01)	0.96(0.91,1.01)	1.09(1.03,1.14)	0.98(0.95,1.00)
Musculoskeletal disorders	1.14(1.09,1.20)	0.93(0.89,0.98)	0.98(0.95,1.01)	1.19(1.13,1.26)	0.96(0.91,1.01)	0.98(0.95,1.01)	1.14(1.07,1.20)	0.92(0.87,0.97)	1.00(0.97,1.03)	1.10(1.05,1.16)	0.90(0.86,0.95)	1.00(0.97,1.03)
Other non-communicable diseases	0.85(0.81,0.89)	1.03(0.98,1.09)	0.98(0.95,1.01)	0.87(0.83,0.92)	1.07(1.01,1.12)	0.98(0.95,1.01)	0.88(0.83,0.93)	1.08(1.02,1.14)	0.99(0.96,1.01)	0.93(0.88,0.98)	1.13(1.07,1.19)	0.99(0.96,1.02)
**Injuries**	0.96(0.89,1.05)	1.10(1.01,1.20)	0.96(0.91,1.00)	0.96(0.84,1.09)	1.27(1.12,1.44)	1.02(0.94,1.10)	0.95(0.83,1.07)	1.36(1.21,1.53)	0.96(0.91,1.03)	0.99(0.88,1.11)	1.39(1.24,1.55)	0.97(0.91,1.03)
Transport injuries	1.07(0.95,1.21)	1.01(0.90,1.13)	0.90(0.84,0.96)	1.03(0.91,1.18)	1.33(1.17,1.51)	0.98(0.91,1.05)	1.05(0.92,1.21)	1.53(1.33,1.76)	0.89(0.83,0.95)	1.10(0.96,1.27)	1.59(1.38,1.82)	0.92(0.85,0.99)
Unintentional injuries	1.02(0.93,1.12)	1.15(1.05,1.26)	0.94(0.89,0.99)	0.87(0.76,0.98)	1.09(0.97,1.23)	1.05(0.98,1.12)	0.90(0.82,0.99)	1.13(1.03,1.23)	0.98(0.94,1.02)	0.95(0.86,1.05)	1.20(1.09,1.32)	1.01(0.96,1.06)
Self-harm and interpersonal violence	0.85(0.69,1.05)	1.11(0.90,1.37)	1.04(0.92,1.18)	1.02(0.84,1.23)	1.36(1.13,1.64)	0.98(0.88,1.09)	0.90(0.73,1.11)	1.41(1.14,1.73)	1.02(0.93,1.13)	0.91(0.76,1.10)	1.39(1.15,1.67)	0.97(0.88,1.07)

Table S6 Association of ASDRs in Different Age Groups with HDI, GII, and ANS in 1990, 2010, 2021, 2035

Age Group	Variable	RR(95%CI)			
		1990	2010	2021	2035
**10-14 years**	ANS	0.97 (0.95, 1.00)	0.99 (0.95, 1.03)	0.98 (0.96, 1.00)	1.00 (0.99, 1.02)
**10-14 years**	GII	1.08 (1.04, 1.13)	1.03 (0.97, 1.09)	1.06 (1.01, 1.10)	1.10 (1.06, 1.14)
**10-14 years**	HDI	0.75 (0.72, 0.78)	0.72 (0.67, 0.77)	0.79 (0.76, 0.83)	0.86 (0.83, 0.89)
**15-19 years**	ANS	0.97 (0.94, 1.01)	0.96 (0.93, 1.00)	0.98 (0.95, 1.01)	1.00 (0.98, 1.01)
**15-19 years**	GII	1.06 (0.98, 1.13)	1.10 (1.03, 1.17)	1.12 (1.06, 1.19)	1.15 (1.11, 1.20)
**15-19 years**	HDI	0.78 (0.73, 0.83)	0.81 (0.76, 0.86)	0.84 (0.79, 0.89)	0.92 (0.88, 0.96)
**20-24 years**	ANS	0.97 (0.93, 1.01)	0.96 (0.92, 1.00)	0.97 (0.94, 1.00)	0.99 (0.97, 1.01)
**20-24 years**	GII	1.06 (0.99, 1.13)	1.11 (1.04, 1.18)	1.14 (1.07, 1.21)	1.18 (1.13, 1.24)
**20-24 years**	HDI	0.79 (0.74, 0.84)	0.82 (0.76, 0.88)	0.86 (0.81, 0.92)	0.95 (0.90, 1.00)

Note: ANS, Adjusted Net Savings; GII, Gender Inequality Indey; HDI, Human Development Index; RR, Rate Ratio.

Table S7 Association of ASDRs with HDI, GII, and ANS in 1990, 2010, 2021, 2035, Adjusted for Regional Fixed Effect

Group	Variable	RR(95%CI)			
		1990	2010	2021	2035
Injury excess	ANS	0.97 (0.95, 0.99)	0.99 (0.95, 1.02)	0.95 (0.92, 0.97)	0.96 (0.92, 1.01)
Injury excess	GII	1.10 (1.01, 1.20)	1.06 (0.99, 1.14)	1.10 (1.02, 1.20)	1.14 (1.07, 1.23)
Injury excess	HDI	0.87 (0.74, 1.02)	1.00 (0.81, 1.23)	0.95 (0.87, 1.04)	0.91 (0.76, 1.08)
Multi-burden	ANS	1.02 (1.00, 1.04)	0.98 (0.92, 1.06)	0.99 (0.95, 1.03)	1.00 (0.99, 1.01)
Multi-burden	GII	0.93 (0.88, 0.98)	1.08 (0.99, 1.17)	1.11 (1.03, 1.20)	1.11 (0.98, 1.25)
Multi-burden	HDI	0.82 (0.79, 0.85)	0.79 (0.51, 1.24)	0.92 (0.88, 0.95)	0.91 (0.85, 0.98)
NCD predominant	ANS	0.98 (0.93, 1.03)	0.96 (0.93, 1.00)	0.95 (0.92, 0.98)	0.97 (0.95, 0.98)
NCD predominant	GII	1.08 (1.00, 1.18)	1.05 (1.02, 1.08)	1.03 (0.99, 1.06)	1.05 (1.00, 1.10)
NCD predominant	HDI	1.03 (0.94, 1.14)	0.97 (0.86, 1.09)	0.96 (0.86, 1.07)	1.04 (0.95, 1.13)
Global	ANS	0.97 (0.94, 1.00)	0.99 (0.94, 1.03)	0.97 (0.94, 1.01)	0.99 (0.98, 1.01)
Global	GII	1.04 (0.97, 1.11)	1.09 (1.05, 1.12)	1.11 (1.06, 1.17)	1.12 (1.03, 1.22)
Global	HDI	0.84 (0.80, 0.89)	0.81 (0.66, 0.98)	0.87 (0.81, 0.93)	0.92 (0.87, 0.97)

Note: ANS, Adjusted Net Savings; GII, Gender Inequality Indey; HDI, Human Development Index; RR, Rate Ratio.

Table S8 Association of ASDRs with HDI, GII, and ANS in 2010, 2021, 2035, with 1-, 2-, and 3-Year Temporal Lags

Variable	Lag Year	RR(95%CI)		
		2010	2021	2035
GII	1	1.11 (1.05, 1.18)	1.15 (1.09, 1.21)	1.16 (1.12, 1.22)
GII	2	1.11 (1.04, 1.18)	1.14 (1.08, 1.20)	1.15 (1.10, 1.21)
GII	3	1.11 (1.04, 1.18)	1.14 (1.09, 1.21)	1.19 (1.13, 1.24)
HDI	1	0.81 (0.76, 0.87)	0.86 (0.81, 0.91)	0.93 (0.89, 0.97)
HDI	2	0.81 (0.76, 0.87)	0.86 (0.81, 0.91)	0.92 (0.88, 0.97)
HDI	3	0.81 (0.76, 0.87)	0.86 (0.82, 0.92)	0.95 (0.90, 1.00)
ANS	1	0.95 (0.91, 0.99)	0.95 (0.92, 0.98)	0.99 (0.97, 1.01)
ANS	2	0.97 (0.94, 1.00)	0.94 (0.91, 0.97)	0.98 (0.95, 1.00)
ANS	3	0.97 (0.94, 1.01)	0.95 (0.92, 0.98)	0.96 (0.93, 0.98)

Note: ANS, Adjusted Net Savings; GII, Gender Inequality Indey; HDI, Human Development Index; RR, Rate Ratio.

Table S9 Annual Percentage Change (APC) in Associations of ASDRs and HDI, GII, and ANS, 2010–2021

Group	Variable	APC	P_{trend}
Global	ANS	0.10% (-0.13%, 0.33%)	0.433
Global	GII	0.14% (-0.16%, 0.44%)	0.388
Global	HDI	0.38% (0.05%, 0.71%)	0.046
Injury excess	ANS	-0.05% (-0.64%, 0.54%)	0.867
Injury excess	GII	0.45% (0.16%, 0.74%)	0.012
Injury excess	HDI	0.78% (-1.63%, 3.26%)	0.543
Multi-burden	ANS	0.31% (0.00%, 0.62%)	0.075
Multi-burden	GII	0.94% (0.50%, 1.38%)	0.002
Multi-burden	HDI	0.11% (-0.27%, 0.48%)	0.583
NCD predominant	ANS	-0.22% (-0.34%, -0.10%)	0.006
NCD predominant	GII	-0.15% (-0.44%, 0.14%)	0.339
NCD predominant	HDI	0.03% (-0.30%, 0.37%)	0.858
Global North	ANS	-0.77% (-1.04%, -0.50%)	0.000
Global North	GII	0.24% (0.03%, 0.45%)	0.050
Global North	HDI	0.62% (0.20%, 1.05%)	0.017
Global South	ANS	0.09% (-0.17%, 0.35%)	0.506
Global South	GII	0.11% (-0.19%, 0.40%)	0.505
Global South	HDI	0.26% (-0.11%, 0.62%)	0.195

Note: ANS, Adjusted Net Savings; GII, Gender Inequality Indey; HDI, Human Development Index; RR, Rate Ratio; APC, Annual Percentage Change.

Table S10 Sex-stratified Associations of ASDRs with HDI, GII, and ANS in 1990, 2010, 2021, and 2035, with Interaction Effects of Sex and HDI, GII, and ANS

Group	Variable	Sex	1990		2010		2021		2035	
			RR (95% CI)	P_{inter}	RR (95% CI)	P_{inter}	RR (95% CI)	P_{inter}	RR (95% CI)	P_{inter}
Injury excess	GII	Female	1.13 (1.02, 1.25)	0.794	1.09 (1.01, 1.18)	0.766	1.18 (1.07, 1.31)	0.838	1.17 (1.08, 1.27)	0.587
Injury excess	GII	Male	1.08 (0.90, 1.28)		1.05 (0.94, 1.17)		1.10 (0.97, 1.25)		1.13 (1.01, 1.26)	
Injury excess	HDI	Female	0.88 (0.78, 0.99)	0.909	0.99 (0.85, 1.14)	0.973	1.07 (0.94, 1.21)	0.716	0.98 (0.88, 1.09)	0.401
Injury excess	HDI	Male	0.84 (0.68, 1.03)		0.98 (0.80, 1.20)		0.91 (0.79, 1.06)		0.90 (0.78, 1.05)	
Injury excess	ANS	Female	0.99 (0.95, 1.03)	0.925	0.99 (0.93, 1.05)	0.981	0.96 (0.91, 1.01)	0.945	0.96 (0.91, 1.01)	0.572
Injury excess	ANS	Male	0.97 (0.90, 1.04)		0.98 (0.90, 1.07)		0.96 (0.90, 1.02)		0.93 (0.87, 1.00)	
Multi-burden	GII	Female	1.04 (0.93, 1.17)	0.480	1.00 (0.80, 1.25)	0.928	1.09 (0.98, 1.22)	0.954	1.10 (1.03, 1.17)	0.804
Multi-burden	GII	Male	0.87 (0.70, 1.08)		1.03 (0.86, 1.24)		1.16 (1.01, 1.33)		1.11 (1.03, 1.21)	
Multi-burden	HDI	Female	0.81 (0.75, 0.88)	0.875	0.76 (0.63, 0.92)	0.869	0.83 (0.75, 0.91)	0.992	0.89 (0.84, 0.94)	0.433
Multi-burden	HDI	Male	0.80 (0.68, 0.93)		0.83 (0.71, 0.96)		0.86 (0.76, 0.97)		0.92 (0.86, 0.99)	
Multi-burden	ANS	Female	0.99 (0.95, 1.03)	0.798	0.97 (0.89, 1.05)	0.722	0.97 (0.93, 1.01)	0.992	1.00 (0.99, 1.02)	0.782
Multi-burden	ANS	Male	1.02 (0.95, 1.10)		0.95 (0.89, 1.02)		0.97 (0.92, 1.02)		1.00 (0.98, 1.02)	
NCD predominant	GII	Female	1.11 (1.06, 1.16)	0.948	1.06 (1.02, 1.11)	0.867	1.05 (1.00, 1.11)	0.971	1.08 (1.02, 1.15)	0.908

Group	Variable	Sex	1990		2010		2021		2035	
			RR (95% CI)	P_{inter}	RR (95% CI)	P_{inter}	RR (95% CI)	P_{inter}	RR (95% CI)	P_{inter}
NCD predominant	GII	Male	1.13 (1.05, 1.22)		1.08 (1.03, 1.14)		1.05 (0.99, 1.12)		1.09 (1.02, 1.16)	
NCD predominant	HDI	Female	1.00 (0.94, 1.07)	0.807	1.02 (0.96, 1.09)	0.977	1.03 (0.96, 1.11)	0.880	1.10 (1.03, 1.19)	0.233
NCD predominant	HDI	Male	1.04 (0.93, 1.16)		0.96 (0.89, 1.04)		0.95 (0.88, 1.04)		1.03 (0.95, 1.12)	
NCD predominant	ANS	Female	1.02 (0.98, 1.05)	0.584	0.98 (0.94, 1.01)	0.871	0.96 (0.93, 1.00)	0.854	0.96 (0.93, 1.00)	0.931
NCD predominant	ANS	Male	0.93 (0.88, 0.99)		0.97 (0.93, 1.01)		0.95 (0.91, 0.99)		0.97 (0.93, 1.00)	

Note: ANS, Adjusted Net Savings; GII, Gender Inequality Indey; HDI, Human Development Index; RR, Rate Ratio.

Figure S3 Restricted cubic spline curves showing the associations of HDI, GII, and ANS with ASDR in 2010, 2021, and 2035

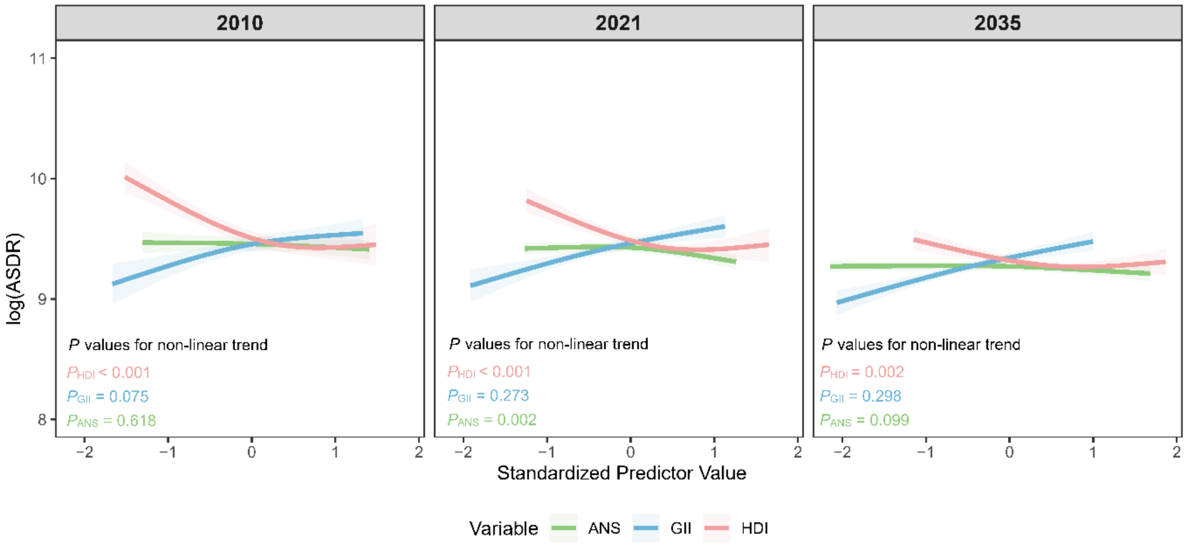

Note: ANS, Adjusted Net Savings; GII, Gender Inequality Indey; HDI, Human Development Index; ASDR, Age-Standardised DALY Rate.

Figure S4. Association of ASDR caused by level 2 causes with Human Development Index (HDI), Gender Inequality Index (GII), and Adjusted Net Savings (ANS) in 2010, 2021, 2035.

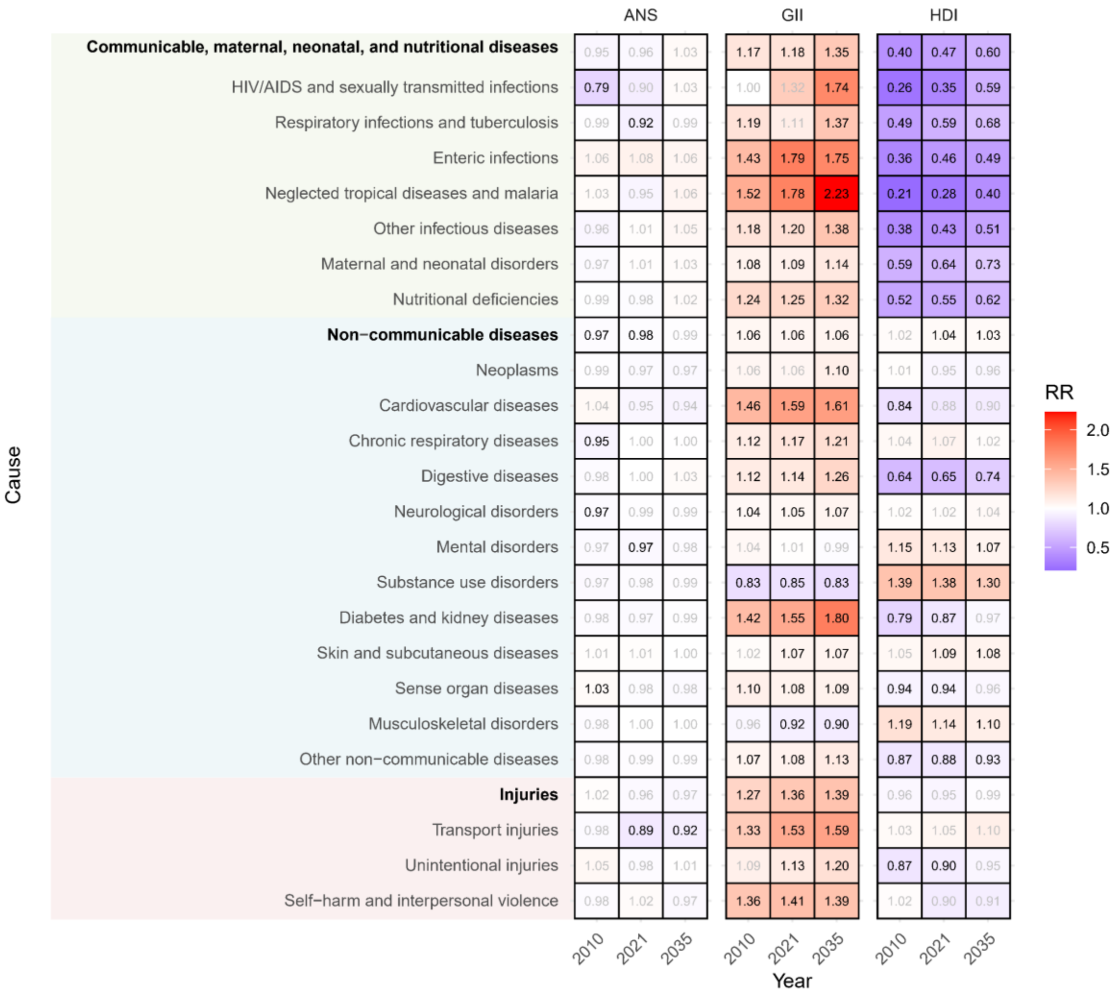

Supplement: Online Supplementary Document [file jogh-16-04188-s001.pdf]
